# DNA degradation in human teeth exposed to thermal stress

**DOI:** 10.1038/s41598-021-91505-8

**Published:** 2021-06-09

**Authors:** Diego Lozano-Peral, Leticia Rubio, Ignacio Santos, María Jesús Gaitán, Enrique Viguera, Stella Martín-de-las-Heras

**Affiliations:** 1 Department of Forensic Dentistry and Medicine, Instituto de Investigación Biomédica de Málaga-IBIMA (CE-18), School of Medicine, University of Malaga, 29071 Malaga, Spain; 2 Supercomputing and Bioinnovation Center, Servicios Centrales de Apoyo a la Investigación, University of Malaga, 29590 Malaga, Spain; 3 Department of Cellular Biology, Genetics and Physiology, University of Malaga, 29071 Malaga, Spain

**Keywords:** Genetics research, DNA, Dentistry

## Abstract

Human identification from burned remains poses a challenge to forensic laboratories, and DNA profiling is widely used for this purpose. Our aim was to evaluate the effect of temperature on DNA degradation in human teeth. Thirty teeth were exposed to temperatures of 100, 200, or 400 °C for 60 min. DNA was quantified by Real-Time qPCR (Quantifiler Human DNA Quantification Kit) and fluorescence spectroscopy (Qubit 3.0 Fluorometer). DNA degradation was evaluated by using STR markers (AmpFLSTR Identifiler Plus PCR Amplification Kit) to determine the allele and locus dropout, inter-locus balance, and degradation slope (observed (Oa) to expected (Ea) locus peak height ratio against the molecular weight). Most of the genomic DNA was degraded between 100 °C and 200 °C. At 100 °C, locus dropout ratios showed significant differences between the largest loci (FGA, D7S820, D18S51, D16S539, D2S1338 and CSF1PO) and amelogenin. Inter-locus balance values significantly differed between all dye channels except between NED and PET. The dropout ratio between D18S51 (NED) and amelogenin (PET) can be recommended for the evaluation of DNA degradation. The Oa/Ea regression model can predict locus peak heights in DNA degradation (R^2^ = 0.7881). These findings may be useful to assess the reliability of DNA typing for human identification in teeth subjected to prolonged incineration.

## Introduction

Human identification from burned remains poses a challenge to forensic laboratories. It is required in a wide range of situations, including major disasters, terrorist attacks, and car accidents, among others^1,2,3,4^, and DNA typing is the usual approach. However, DNA is highly degraded in extremely charred bodies, hampering short tandem repeat (STR) analysis^5^.

Researchers have focused mainly on morphological, color and structural changes in bones and teeth heated at different temperatures (up to 1000 °C) rather than on the feasibility of nuclear DNA analysis^5,6,7,8,9,10^. In cases of severe degradation, a full DNA profile is more readily obtained from hard (bone and teeth) than soft tissues^2^. Teeth are the hardest tissue in the human body, and their pulp is well protected by dentin, enamel, and cementum, explaining their frequent utilization to obtain DNA from compromised remains^9,11,12^.

The survival of dental DNA is highly dependent on the temperature and duration of the heat exposure^13–15^. It has generally been reported that dental DNA can withstand temperatures up to 400 °C for one hour but that its quantity and quality are superior after exposure to lower temperatures for shorter time periods (1–20 min), facilitating forensic identification^9^. Only a few studies have studied teeth exposed for more than 15 min at temperatures above 90 °C^16,17,18,19^. Most researchers have reported that it is very difficult to extract DNA from teeth after their exposure to temperatures of 200–400 °C^18,19,20^, and no consensus has been reached on the degree of cremation at which teeth will still yield nuclear DNA signals^7^.

Real-Time PCR is the preferred technique for forensic samples because of its wide dynamic range and high sensitivity, although fluorescence assays can also be used for the rapid quantification of single-source samples^9,21^. However, successful forensic identification by genotyping requires DNA of adequate quantity and quality*.* There is a need for in-depth investigation to determine the degree of DNA degradation in teeth burned at different temperatures and time of exposures. The objective of this study was to quantify the DNA in teeth exposed for 60 min to temperatures of 100, 200, or 400 °C and evaluate its degradation by examining locus dropout, peak height dropout ratio, inter-locus balance, and degradation slope.

## Material and methods

### Samples

Forty healthy human teeth (molars and premolars) were obtained from adult patients (25 females and 15 males) aged between 19–74 years (mean of 42.7 years) at dental clinics in Malaga and Cadiz (Spain). All patients provided their written informed consent to participation in this study, which was approved by the Human Research Ethics Committee of the University of Malaga (CEUMA 2013-0048-H) and conducted in accordance with the Declaration of Helsinki and with national data protection legislation (Organic Law 3/2018).

All studied teeth were extracted for valid clinical reasons (periodontal disease or orthodontic treatment) and were free of cavities, endodontics, or reconstruction. After extraction, they were washed with distilled water, and their external surfaces were cleaned with curettes to remove any extraneous material. The teeth were then stored under controlled conditions of 21 °C and 65% humidity until their dispatch to the laboratory. In order to remove the exogenous DNA from tooth surfaces^22–26^, teeth were immersed in 3% sodium hypochlorite solution for 1.5 min and rinsed with sterile water to remove any remaining bleach. Next, samples were irradiated with 256-nm UV light (Telstar Mini V/PCR. Telstar Industrial S.L., Terrassa, Spain) for 10 min. Samples were then randomly divided into four groups of 10 teeth each.

### Experimental conditions for furnace incineration

Previous studies reported that DNA profiling is difficult to obtain from teeth after their exposure to 400 °C^9,27^. The temperature of fire and time of exposure of corpses can vary in the different forensic scenarios. For example, a fire could be subjected to many different temperatures depending on the origin or contributing factors. Based on these studies, we have selected time and temperature of heat exposure to embrace multiple circumstances in a forensic context in the current research. One group of teeth (n = 10) was kept at room temperature and served as controls. The remaining groups were exposed to temperatures of 100, 200 or 400 °C for 60 min. Samples were individually placed in 99% alumina crucibles and heated in a muffle furnace (Nabertherm LT 40/12, Nabertherm GmbH, Germany) to the corresponding temperature at a heating rate of 10 °C/min and then maintained at this temperature for 60 min before being removed and left to cool at room temperature.

### DNA extraction

Samples were pulverized in liquid nitrogen with a 6770 Freezer/Mill (SPEX SamplePrep, LLC, Metuchen, USA). Powder teeth (0.5 g) were demineralized and lysed in a buffer containing 500 µL of 0.5 M EDTA, 35 µL SDS (10%) and 100 µL of proteinase K (20 mg/mL). Following 24-h incubation at 37 °C with continuous agitation (300 rpm), the tubes were centrifuged at 13,000 rpm for 3 min. The supernatants were transferred and mixed with 500 µL of phenol:chloroform:isoamyl alcohol (25:24:1) and centrifuged again at 13,000 rpm for 3 min. Then, the supernatants were taken and added to Centricon-100 concentrators (Centricon-100, Millipore, Bedford, MA) and centrifuged at 2500 rpm for 20 min. Finally, concentrators were placed into 1.5 mL microcentrifuge tubes and DNA was recovered in 30 µL of the elution buffer (10 mM Tris/HCl, pH 8.5) after centrifugation at 2500 rpm for 20 min. Blanks were included in DNA extraction procedures and PCR amplifications. Samples were stored at − 20 °C before DNA analysis.

### DNA quantification

Two methods were applied to quantify the DNA in samples: The Quantifiler Human DNA Quantification Kit (Thermo Fisher, Foster City, California, USA), using an Applied Biosystems 7500 Real-Time PCR system according to the manufacturer’s protocol^28^; and the Qubit 3.0 Fluorometer (Life Technologies, Carlsbad, California, USA), following the manufacturer’s instructions (Qubit dsDNA BR and Qubit dsDNA HS Assay Kits)^29,30^.

### DNA integrity

DNA integrity was examined by digital electrophoresis using a 2100 Bioanalyzer Instrument—Agilent (Agilent Technologies, Waldbronn, Germany) to visually confirm DNA degradation^31^. The size distribution of DNA molecules was visualized and analyzed using the Agilent High Sensitivity DNA Kit^32^.

### DNA degradation analysis

STR genotyping was performed with an AmpFLSTR Identifiler Plus PCR Amplification Kit. PCRs were performed using the C1000 Touch Thermal Cycler (Bio-RAD) with the following conditions: 95 °C for 11 min followed by 28 cycles of 94 °C for 20 s and 59 °C for 3 min, and a final extension at 60 °C for 10 min. PCR products were kept at 4 °C (Thermo Fisher)^33^. The amount of DNA template in all samples is shown in the Supplementary Dataset (Table S11). Amplification products were analyzed by capillary electrophoresis injection using an ABI PRISM 3130 Genetic Analyzer in accordance with the manufacturer's instructions. Samples were analyzed with GeneMapper Software v4.0 from Applied Biosystems, selecting a detection threshold of 50 relative fluorescence units (RFU)^20^. For heterozygous loci, the mean for the two alleles was calculated and used to correct for a possible imbalance between peaks. Peak heights of homozygous loci were also divided by two to normalize for diploidy^34,35^.

DNA degradation was studied by considering locus dropout, peak height dropout ratio, inter-locus balance, and degradation slope, as follows:

1. Locus dropout was determined as the percentage of amplified loci in each group of teeth. This value was compared with the sum of locus peak heights (in RFU) for each heat condition (Table S1).
2. Locus peak height dropout ratio was calculated as the percentage peak height dropout of the smallest locus to largest loci^23,35^. In the control and 100 °C groups, the smallest locus, amelogenin (106–112 bp), was compared with the six largest: FGA (196–348 bp), D7S820 (253–293 bp), D16S539 (248–296 bp), D18S51 (264–351), CSF1P0 (280–316 bp), and D2S1338 (291–359 bp) (Tables S2–S7, respectively).
3. The inter-locus balance was calculated by comparing the deviations of the peak height for each locus from the sample mean. Signal values were normalized by dividing the difference by the square root of the mean peak height per locus^36^ (Table S8).
  $$Interlocus\,\, balance = \frac{locus\,\, height - mean\,\, peak\, height\,\, from\,\, 16\,\, loci}{\sqrt{mean\,\, peak\,\, height\,\, from \,\,16\,\, loci}}$$
  The mean inter-locus balance (in arbitrary units) was analyzed by dye group (6-FAM, VIC, NED and PET, hereafter FAM, VIC, NED and PET, respectively) in the control and 100 °C groups (Table S9).
4. The sum of peak heights for each locus was used to evaluate the degradation slope of DNA produced by the burning. Values for the 100 °C and control groups were considered as observed allelic peak (Oa) and expected allelic peak (Ea), respectively (Table S10). The Oa/Ea ratio was calculated for each locus^37^. After studying different regression models, the curve with the highest R^2^ value was selected.

### Statistical analysis

R statistical software v.3.1.0 and RStudio version 1.1.442 (http://www.r-project.org)^38^ and Statgraphics Centurion 18 (Statgraphics Technologies, Inc., USA) were used for statistical analysis. Figure 3 and Figure S7 were created in R using the package ggplot2^38^. Significance was assessed by one-way analysis of variance (ANOVA), considering p < 0.05 as statistically significant. When F-values were significant, the Tukey honestly significant difference (HSD) post-hoc test was applied for multiple comparisons.

## Results

### DNA quantification

Table 1 exhibits the mean, standard error of mean (SEM), minimum, and maximum DNA concentrations for the control and incinerated groups. DNA values obtained with Quantifiler and Qubit significantly differed between the control group and all incinerated groups (p < 0.01) but not among the incinerated groups (Table 1).

**Table 1:** DNA concentration of control and incinerated group of teeth using Quantifiler and Qubit methods.

Teeth group	Quantifiler				Qubit			
	Average DNA value (ng/µL)	Min (ng/µL)	Max (ng/µL)	SEM	Average DNA value (ng/µL)	Min (ng/µL)	Max (ng/µL)	SEM
Control	133.95 (*)	65.34	226.96	19.13	101.76 (*)	53.3	180	12.45
100 °C	10.83	0.24	35.87	4.35	27.96	0	171	23.71
200 °C	1.3 × 10^–2^	0	4.3 × 10^–2^	5.2 × 10^–3^	3.4 × 10^–2^	0	0.15	4.3 × 10^–2^
400 °C	1.0 × 10^–4^	0	8,1 × 10^–4^	2.6 × 10^–4^	0	0	0	-

*Significant differences (p < 0.01) between control group and all incinerated groups with Quantifiler and between control group and incinerated group at 100 and 200 °C with Qubit.

Among the teeth incinerated at 100 °C, DNA was detected in 100% of the samples using Quantifiler method and in 60% using Qubit. Among those incinerated at 200 °C, DNA was detected in 80% with Quantifiler and in 30% with Qubit. In the group incinerated at 400 °C, DNA was detected in 20% with Quantifiler and in 0% with Qubit.

The minimum DNA concentration detected with Quantifiler Human DNA Quantification Kit was 0.215 pg/μL (400 °C group) and 0.053 ng/μL with Qubit (200 °C group). Quantifiler results were adopted for calculation of DNA template in STR PCR.

### DNA integrity

DNA bands were generally larger in the control teeth than in those burned at 100 °C for 60 min (Figs. S1 and S2). In almost all control teeth, DNA bands were accumulated closer to the largest molecular weight marker (10,380 bp) in comparison to the burned teeth (Fig. S1). DNA smearing was more evident in the teeth burned at 100 °C (Fig. S2). No DNA was detected by the Bioanalyzer Instrument in teeth incinerated at 200 or 400 °C.

### DNA degradation

#### Locus dropout and STR profiles

**Figure 1:**
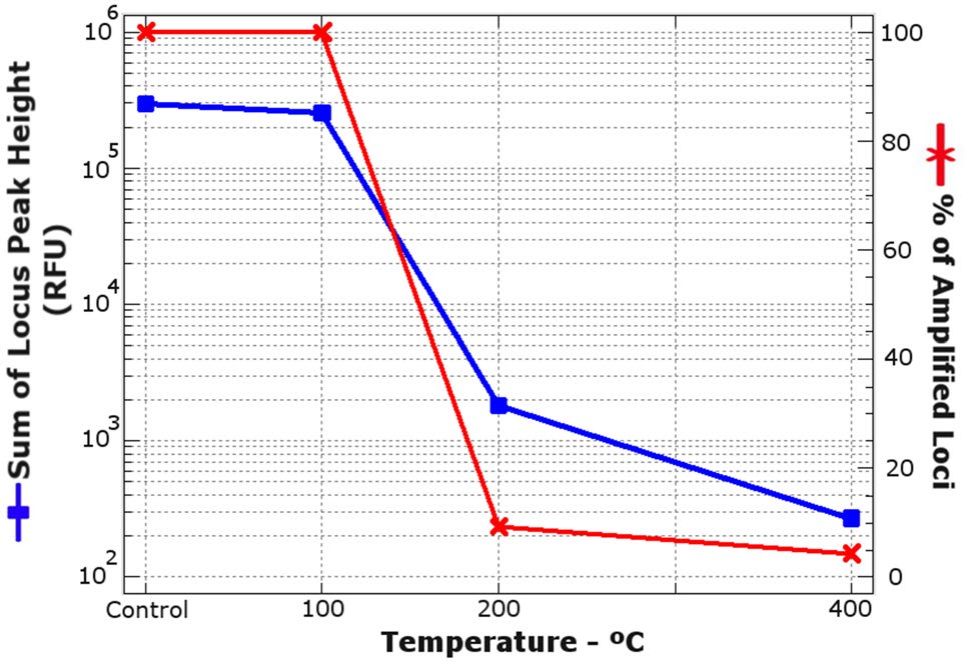
Effect of temperature on STR amplification. Sum of locus peak heights in relative fluorescence units (RFU) and percentage of amplified loci against temperature (without incineration—control, 21 °C- and with incineration at 100, 200 and 400 °C for 60 min). Note that the sum of peak heights is represented to the power of 10 to improve the visualization of the figure.

All STR markers were detected in all samples from the control group and the group incinerated at 100 °C (Fig. 1). The percentage of amplified loci was 9.38% at 200 °C and 4.38% at 400 °C (Table S1; Fig. 1). The sum of locus peak heights was 299,564.0 RFU in the control group, 255,796.5 RFU in the 100 °C group (14.6% reduction versus controls), 1809.5 in the 200 °C group (99.4% reduction versus controls), and 273 RFU in the 400 °C group (99.9% reduction versus controls).

Electropherograms analyzing the quality of DNA profiles showed an inverse correlation in peak height signals with increasing molecular weight of loci (degradation slope) at 100, 200, and 400 °C (Figs. S3–S6). The recovery of larger loci is much less probable at 200 and 400 °C (Table S1, Locus dropout by size).

#### Locus peak height dropout ratio

**Figure 2:**
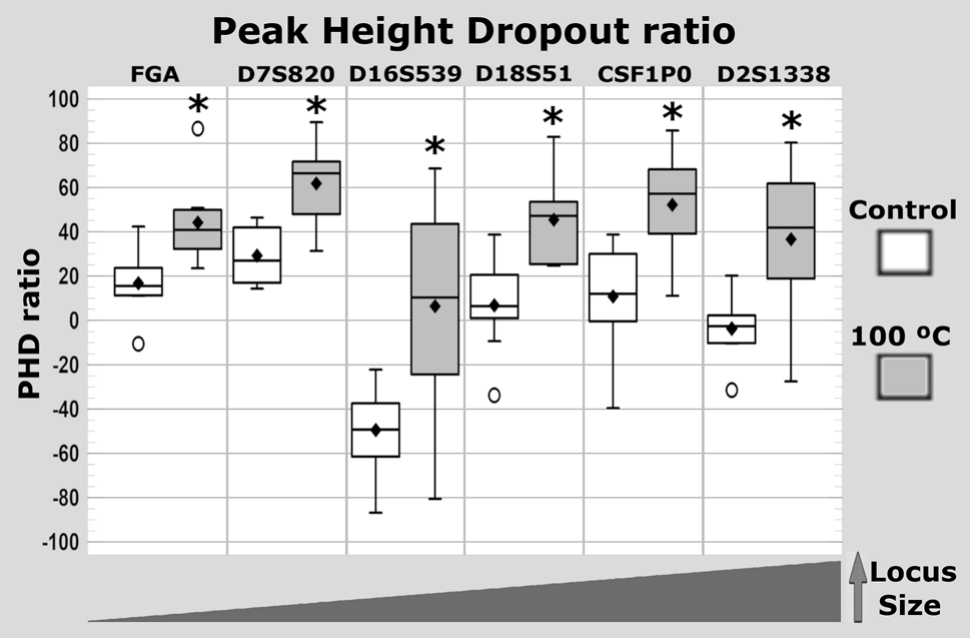
Box and whisker plot of locus peak height dropout (PHD) ratio in control and 100 °C groups. Dropout ratio was calculated as the percentage peak height dropout of the smallest locus (amelogenin) relative to the largest loci (FGA, D7S820, D16S539, D18S51, CSF1P0 and D2S1338). *Significant differences (p < 0.01) between control group teeth and teeth incinerated at 100 °C for 60 min.

In control samples, mean peak height dropout ratios for the amelogenin locus relative to FGA, D7S820, D18S51, and CSF1PO were 16.71%, 29.15%, 6.75%, and 10.76%, respectively, whereas the results for amelogenin relative to D16S539 and D2S1338 showed negative dropout ratios (− 49.43% and − 3.76%, respectively) due to the higher peak height for these loci than for amelogenin. At 100 °C, mean peak height dropout ratios for amelogenin relative to FGA, D7S820, D16S539, D18S51, CSF1P0, and D2S1338 were 44.06%, 61.80%, 6.33%, 45.36%, 52.22%, and 36.60%, respectively (Tables S2–S7). Locus peak height dropout ratios significantly differed between the control group and the 100 °C group (sum of squared deviations = 3739, 5330, 15,546, 7454, 8595, and 8147 for FGA, D7S820, D16S539, D18S51, CSF1P0, D2S1338, respectively; 1 degree of freedom; p < 0.01) (Fig. 2).

Locus peak height dropout ratio, inter-locus balance, and degradation slope (regression model) analysis were carried out between control samples and those exposed to 100 °C, as a low number of loci were amplified from samples exposed to 200 and 400 °C. In fact, 3 and 12 loci were detected in two samples of the 200 °C group, and 7 loci in one sample of the 400 °C group (Table S2).

#### Inter-locus balance by locus and dye color

**Figure 3:**
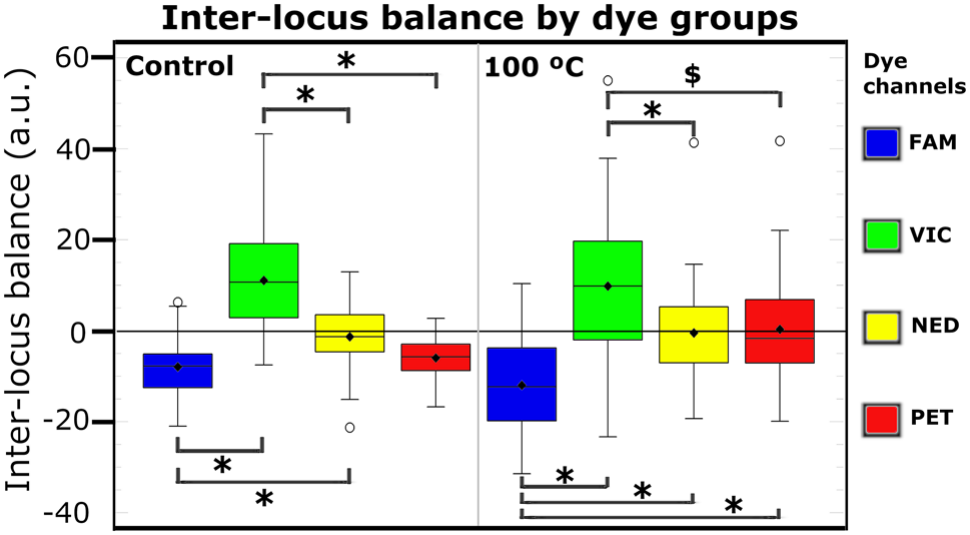
Inter-locus balance in arbitrary units (a.u.) is depicted by dye group (FAM, VIC, NED and PET) and temperature (control − 21 °C and 100 °C). Significant differences: *p < 0.01, ^$^p < 0.05 (Created using R Core Team, 2013 with package ggplot2)^38^.

Inter-locus balance values close to zero indicate a well-balanced profile, while negative and positive values indicate that the locus has peak heights below or above the mean, respectively (Fig. S7). Figure 3 shows inter-locus balance values for the control and 100 °C groups by dye group. Greater general dispersion of inter-locus values was observed in the 100 °C group than in controls. In the control group, significant differences were found between FAM and NED groups and between the VIC group and the remaining dye groups (p < 0.01). In the 100 °C group, significant differences were found between all dye groups (p < 0.01 or p < 0.05), except between NED and PET.

#### Degradation slope

**Figure 4:**
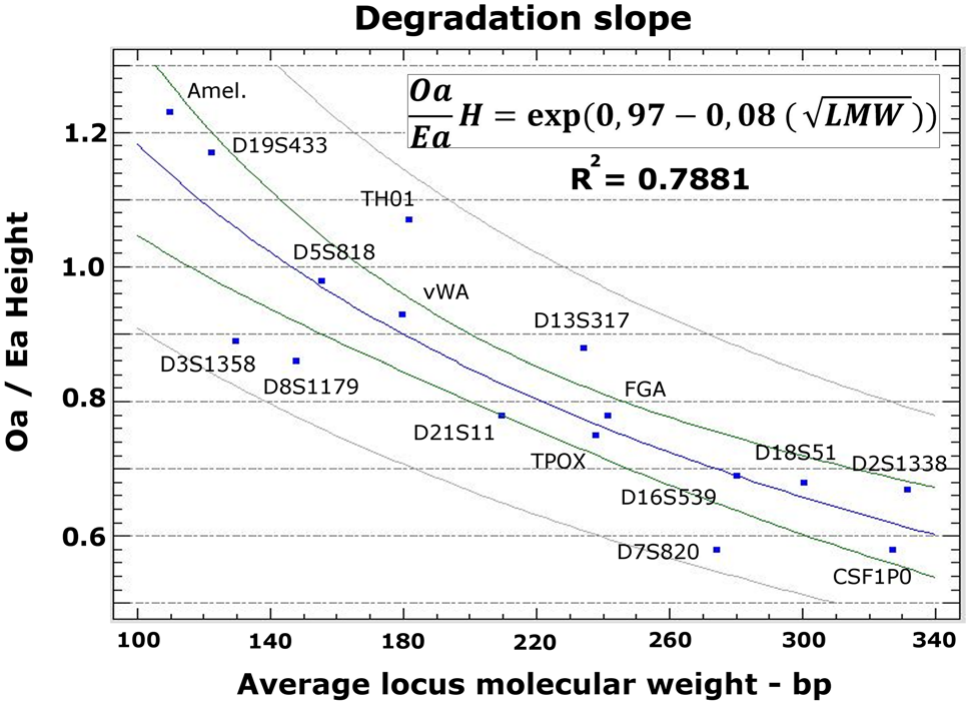
Degradation slope caused by temperature effect. Oa/Ea ratio for each locus is plotted against the molecular weight in base pairs for the control and 100 °C groups. Simple regression model of “Y-Logarithmic X-Square root” is represented by blue curve. Green and grey lines represent the confidence (95%) and prediction limits of the model, respectively. The equation derived from this model is shown (*Oa/Ea H* Oa/Ea height ratio, *LMW* locus molecular weight in base pairs).

Figure 4 plots the Oa/Ea ratio for each locus against the molecular weight in base pairs for the control and 100 °C groups. The “Y-logarithmic X-square root” gave the best R^2^ value (0.7881). The equation derived from the simple regression model is shown. Intermediate-small molecular weight locus (D5S818, vWA and TH01) had Oa/Ea ratios close to 1. The highest Oa/Ea ratio was for the amelogenin locus (1.23) (Fig. 4).

## Discussion

### DNA detection and integrity

Real-Time PCR methods (qPCR) have been developed for the accurate measurement of DNA quantity^39^, and a fluorescence spectroscopy technique (Qubit 3.0 Fluorometer) is also available for utilization in forensic samples^9^. In this study, we have exposed the teeth up to 400 °C as previous studies have reported that DNA profiling is difficult to obtain from teeth after that temperature of exposure^9,27,40^.

A decrease in the amount of DNA was observed from unheated control teeth to those heated at 100, 200, and 400 °C for 60 min. In previous studies, it has been observed that tooth DNA concentration is not influenced by age or sex^35,41^. Application of qPCR and Qubit quantitative methods showed significant differences in the amount of DNA between control and incinerated teeth in line with previous findings^42,43^ but not among the incinerated groups, although these showed a wide dispersion of values, especially with Qubit. Both quantification methods revealed a major reduction in DNA concentration after exposure to 100 °C for 60 min (Table 1).

Although DNA measurements have been reported to vary among different quantification methods^44^, we found no significant difference in mean concentrations between Quantifiler and Qubit in the control or 100 °C groups. However, DNA was detected in all teeth exposed to 100 °C with Quantifiler and in only 60% of these with Qubit. DNA was also more frequently detected in samples exposed to 200 or 400 °C with the Quantifiler Human DNA Quantification Kit than with the Qubit method. The LOD of the Quantifiler Human DNA Quantification Kit is < 23 pg/μL (< 13 target copies) according to the manufacturer, although LODs of 6 pg per reaction^45^ and < 5 pg/μL of DNA^39^ have also been described. In our study, the minimum DNA concentration detected with Quantifiler was 0.215 pg/μL. At this range, stochastic or statistical effects of low copy locus sampling may produce significant variability in assay results, and they may explain the unexpected allele typing after exposure to 400 °C of incineration, which has previously been observed^39^. According to the present results, a minimum Quantifiler Human DNA Quantification-measured DNA concentration of 0.24 ng/μL may allow full STR typing (100% amplification of expected locus) and a minimum Quantifiler Human DNA Quantification-measured DNA concentration of between 0.2 ng/μL and 0.2 pg/μL may allow a partial STR profile to be obtained. Regarding to Qubit quantitation, although the minimum DNA concentration detected in our study was 0.053 ng/μL, the minimum Qubit-measured DNA concentration was 0.15 ng/μL for a partial STR profile and 4.8 ng/μL for a complete STR profile. Surprisingly, full or partial STR typing was obtained in several samples in which DNA was not detected with Qubit. The Quantifiler Human DNA Quantification Kit yielded more reliable and accurate results and an improved sensitivity for highly degraded samples in comparison to Qubit.

Visualization of DNA fragments up to 10 kb on Agilent 2100 Bioanalyzer Instrument provided visual confirmation of DNA degradation. Smear of DNA in electrophoresis results from DNA fragmentation due to degradation. In the present study, smear of DNA was more evident in teeth exposed to thermal stress than in controls because of the greater fragmentation of molecular DNA, as reported in a previous study^46^.

### Temperature effect on locus dropout and STR profiles

According to the present findings, human genetic identification is possible from teeth incinerated at 100 °C for up to 60 min but almost impossible from teeth exposed to temperatures of 200 °C and above. The highest locus dropout was observed in samples exposed to 200 °C and 400 °C (Fig. 1), and amelogenin Y alleles were also missing in two amplified male samples exposed to these temperatures. When DNA amounts are small, there is an increase in heterozygote peak height imbalance and in the number of undetected loci at 200 and 400 °C (Figs. S5, S6), mainly due to stochastic effects in pre-PCR sampling^47^. However, the undetected amelogenin Y allele at 400 °C may also result from the presence of a technology-related artefact peak overlapping the Y signal known as a dye blob (Fig. S6), described in detail elsewhere^48^.

The heat exposure on the DNA molecules produces the deamination of cytosine that is directly related to the increase of the temperature and the duration of the exposure^49,50^, generating a mutated daughter strand. This is an important issue in methods where DNA sequence is necessary, such as mtDNA typing by Sanger-type sequencing or STRs by DNA Next Generation Sequencing^51^. Because deamination of cytosine may result in inhibition of PCR or mutagenic DNA products, this is an important issue in methods where DNA sequence is crucial. In methods that rely on the amplicon length rather than the exact sequence (i.e. short tandem repeats used in human identification), deamination of cytosine may also originate a possible change of the nucleotide sequence and, if the impact is deeper, the loss of STR markers starting from the high molecular weight ones. Thermal degradation of DNA breaks covalent bonds within each DNA strand, leading to DNA fragmentation^52^. This study^52^ found that plasmid DNA degradation started after 5 min at 130 °C and was complete at around 190 °C. STR profiling is dependent on the temperature and the duration of exposure, due to DNA fragmentation. Most studies have exposed teeth for short time periods of 2–15 min and have obtained fully amplified loci up to temperatures of 300 °C^9,12,20^, while STR typing was found to be reliable up to 16 h of exposure when the temperature was 90 °C^17^. The present teeth were exposed for 60 min, and loci were fully amplified up to 100 °C, whereas only partial STR profiles could be obtained at 200 °C and 400 °C. The lesser degradation observed in teeth may be explained by the hardness of the surrounding tissue, which acts as a DNA protector^9,11,22^.

### Locus peak height dropout ratio

The locus peak height dropout ratio has previously been studied in teeth stored for long time periods^22,35^. In the present study, all samples were subjected to the same storage conditions and DNA extraction procedure; therefore, the locus peak height dropout ratio results should not be influenced by differences in treatment between control and test teeth, as in previous studies^37^.

Statistically significant peak height dropout was observed in the six largest loci after exposure to 100 °C for 60 min. The most affected amplicons were D7S820 and CSF1P0, with dropout ratios of 61.80% and 52.22%, respectively, while the least affected were D16S539 and D2S1338 (Fig. 2). The lowest molecular weight corresponds to the FGA locus, followed by D7S820, D16S539, D18S51, CSF1P0, and D2S1338. Although we cannot rule out a low template amplification or inhibition effect, the lack of a precise correlation between locus peak height dropout rate and locus size may have two main explanations. First, primers specific to the DNA target may vary among loci, leading to the over- or under-amplification of some PCR products in comparison to others^53^. Second, loci in the AmpFLSTR Identifiler Plus PCR Amplification Kit are tagged with different dyes (blue, green, yellow, and red channels for FAM, VIC, NED, and PET, respectively), and some channels can show different peak heights or signal intensities^54,55^. Given its possible effects on the peak height of each locus and therefore on the locus peak height dropout ratio in pairwise comparisons, the inter-locus balance was studied by locus and dye color.

### Inter-locus balance analysis by loci and dye color

Mattayad et al.^54^ found that the inter-locus balance by dye color and locus was superior with the AmpFLSTR Identifiler Plus PCR Amplification Kit *versus* Investigator IDplex Plus Kit (QIAGEN, Hilden, Germany). In the present study, blue (FAM) and green (VIC) channels had higher locus peak heights with the AmpFLSTR Identifiler Plus PCR Amplification Kit. Debernardi et al.^55^ reported evidence of some differences in sensitivity of the blue channel between two ABI 3130xl Genetic Analyzers. Other kits currently in the market have integrated quality sensors and may be useful in future research. Various issues should be considered when using locus peak height dropout ratios to study DNA degradation. First, dropout ratios from loci with similar molecular weight but tagged with different dyes are not comparable if they differ in inter-locus balance. Second, differences in inter-locus balance values between kits mean that a variation in peak height dropout ratio can even be observed in the same locus tagged with the same dye. Third, inter-laboratory studies using the same kit must consider dye channel sensitivities to avoid possible differences in genetic analyzers, spectral calibrations, and/or dye matrix procedures between laboratories. Inter-laboratory and inter-instrument validation are crucial for comparable and reliable results. In the present study, inter-locus balance values were worse for VIC and FAM markers and better for NED and PET markers, which showed more similar values between them (Fig. 3). Although D2S1338 (VIC) was the largest locus, its peak height dropout ratio was the second lowest (36.6%), while D7S820 (FAM), the second smallest locus, had the highest peak height dropout ratio (61.8%). The fact that inter-locus balance values were highest for VIC and lowest for FAM may explain these results. In contrast, a better inter-locus balance was observed for loci tagged with NED and PET dyes under both control and heated conditions. Our results confirm that the dye color can influence the signal peak height; therefore, not only the locus molecular weight but also the fluorescent dye of each locus should be taken into account in DNA degradation studies. According to the locus dropout ratio and inter-locus balance results, the comparison between amelogenin (PET dye) and D18S51 (NED dye), the third largest locus, with a 45.36% dropout ratio, can be recommended for investigations of DNA degradation.

### Degradation slope and expected peak heights

The degradation or “ski” slope describes the downward trend of a DNA electropherogram with increasing molecular weight^37^, which is attributed to DNA degradation and even observed in pristine DNA^37^. Differences in the PCR efficiency for each locus may lead to different loci signal intensities^55^. In addition, according to the present and previous findings^54,55^, locus peak height signals can also vary among marker dyes.

A reduction in Oa/Ea values is observed with increasing molecular weight, which can be considered as DNA degradation from exposure to 100 °C (Fig. 4). Oa/Ea rates were < 1 in loci with molecular weight > 200 bp, demonstrating that the locus peak height dropout due to temperature is greater in these loci. Interestingly, Oa/Ea values were > 1 for the two smallest loci, attributable to the amount of available template DNA and a greater PCR efficiency^55^. DNA is more fragmented after heat exposure, and it is more difficult to amplify loci with higher molecular weight, reducing the RFU signal detected. In contrast, the amplification of loci with lower molecular weight is less affected by DNA degradation. Consequently, there is a greater formation of primer/template complexes (binary complexes) with a larger amount of small amplicon DNA. In addition, there is a greater availability of free polymerase not bound to DNA for these binary complexes (ternary complexes formation), due to the lesser formation of binary complexes for amplicon loci of higher molecular weight. Hence, PCR efficiency may be higher for smaller loci in samples incinerated at 100 °C, and the RFU signal tended to be higher than in control samples. Loci with molecular weight between 150–200 bp showed intermediate Oa/Ea values of around 1. Therefore, the most consistent results were obtained for loci with a molecular weight < 200 bp. The Oa/Ea regression model obtained with the AmpFLSTR Identifiler Plus PCR Amplification Kit may estimate locus peak heights as a function of molecular weight in DNA degradation in teeth incinerated at 100 °C for up to 60 min.

Study limitations include its development in a controlled laboratory environment. It did not take into account the influence of the accelerant type or fire extinction method, among other relevant factors, including the duration of exposure. Furthermore, it was conducted in teeth lacking the protection provided by alveolar bone or maxillary bone. In addition, there was no exploration of the protective effect of facial tissues against external insults like high temperatures. Further research is needed to embrace other forensic scenarios with different exposure times and temperatures.

## Conclusion

After 60 min of incineration, all STR loci were detected in all teeth exposed to 100 °C, but genetic identification was almost impossible from teeth exposed to temperatures of 200 °C and above. STR amplification of DNA samples is time-consuming and can be costly, so determination of the minimum amount of DNA needed for full or partial STR typing may be of value for forensic laboratories. A statistically significant difference in the peak height dropout ratio of amelogenin relative to FGA, D7S820, D16S539, D18S51, CSF1P0, and D2S1338 was observed between unheated teeth and teeth exposed to 100 °C. NED and PET had a better inter-locus balance at 100 °C than the other dyes, and our locus peak height dropout ratio findings lead us to recommend the comparison of D18S51 (NED) with amelogenin (PET) in studies of DNA degradation. In addition, the slope ratio (Oa/Ea) obtained with the AmpFLSTR Identifiler Plus PCR Amplification Kit may estimate locus peak heights as a function of molecular weight for evaluating DNA degradation at up to 60 min of exposure to 100 °C. This study was carried out in a specific range of temperature and time of exposure, however, it adds to previous studies findings, and together may be useful to assess the reliability of dental DNA typing for human identification.

## Supplementary Information

Supplementary Information 1.

Supplementary Information 2.
