# Supplementary material for: DNA degradation in human teeth exposed to thermal stress
Source: Sci Rep. 2021 Jun 9;11:12118. doi: 10.1038/s41598-021-91505-8 (PMC8190102; doi:10.1038/s41598-021-91505-8)
Supplement: Supplementary file 2 — Supplementary Information 2. [file 41598_2021_91505_MOESM2_ESM.pdf]

DNA degradation in human teeth exposed to thermal stress

Diego Lozano-Peral[1,2†], Leticia Rubio[1†*], Santos I[1], María Jesús Gaitán[1], Enrique Viguera[3], Stella Martín-de-las-Heras[1]

[1] Department of Forensic Dentistry and Medicine, Instituto de Investigación Biomédica de Málaga (CE-18). University of Malaga, 29071 Malaga, Spain. Leticia Rubio E-mail address: lorubio@uma.es; Ignacio Santos E-mail address: isantos@uma.es; Maria Jesus Gaitan E-mail address: mjgaitan@uma.es; Stella Martin-de-las-Heras E-mail address: smdelasheras@uma.es

[2] Supercomputing and Bioinnovation Center, Instituto de Investigación Biomédica de Málaga (CE-18). University of Malaga, 29590 Malaga, Spain. Diego Lozano-Peral E-mail address: dlozanop@uma.es

[3] Department of Cellular Biology, Genetics and Physiology, University of Malaga, 29071 Malaga, Spain. Enrique Viguera E-mail address: eviguera@uma.es

†These authors contributed equally to this work

***Corresponding author:**

Leticia Rubio
E-mail address: lorubio@uma.es
Department of Forensic Dentistry and Medicine
University of Malaga
29071 Malaga, Spain

ORCID iD :

Leticia Rubio https://orcid.org/0000-0002-8233-624X
Diego Lozano-Peral https://orcid.org/0000-0001-8072-0355
Stella Martin-de-las-Heras https://orcid.org/0000-0002-1554-951X
Enrique Viguera https://orcid.org/0000-0001-5475-3807

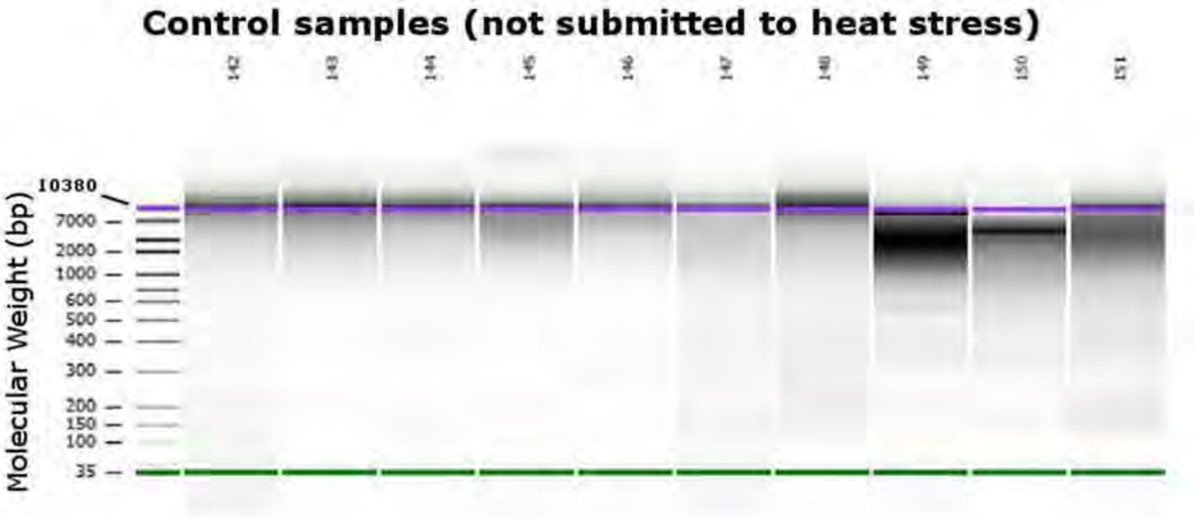

Fig. S1. Digital DNA electrophoresis from control samples (not submitted to heat stress) obtained by Bioanalyzer 2100. Purple and green lines represent the highest (10380 pb) and lowest (35 bp) molecular weight markers, respectively. The grouping of blots (divided lanes with white spaces) was cropped from different digital gel images (additional modifications were not applied). Source data showing full-length digital gels and electropherograms are provided as Supplementary Information File.

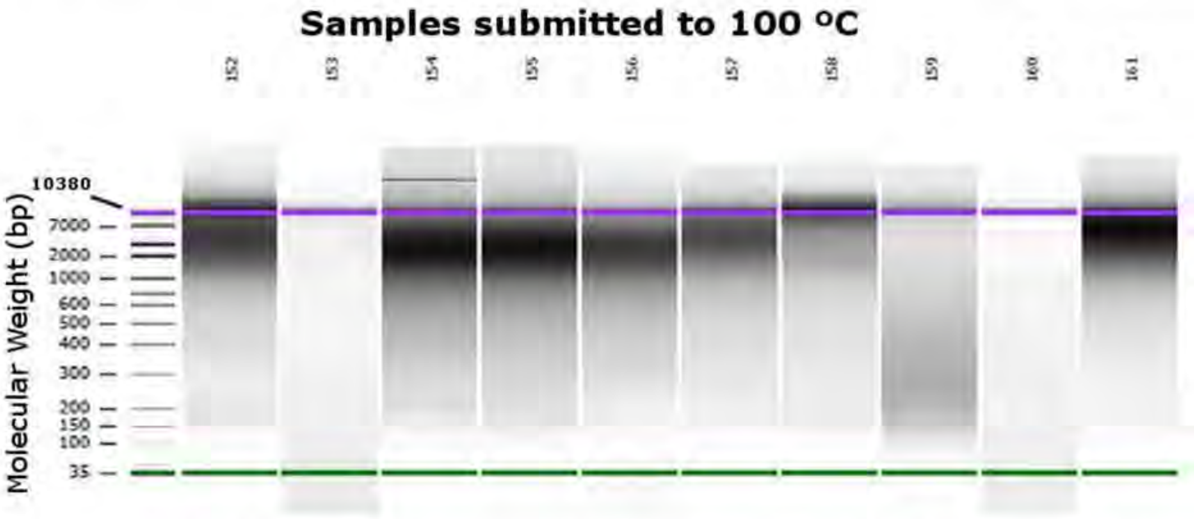

Fig. S2. Digital DNA electrophoresis from samples submitted to 100 °C obtained by Bioanalyzer 2100. Purple and green lines represent the highest (10380 pb) and lowest (35 bp) molecular weight markers, respectively. The grouping of blots (divided lanes with white spaces) was cropped from different digital gel images (additional modifications were not applied). Source data showing full-length digital gels and electropherograms are provided as Supplementary Information File.

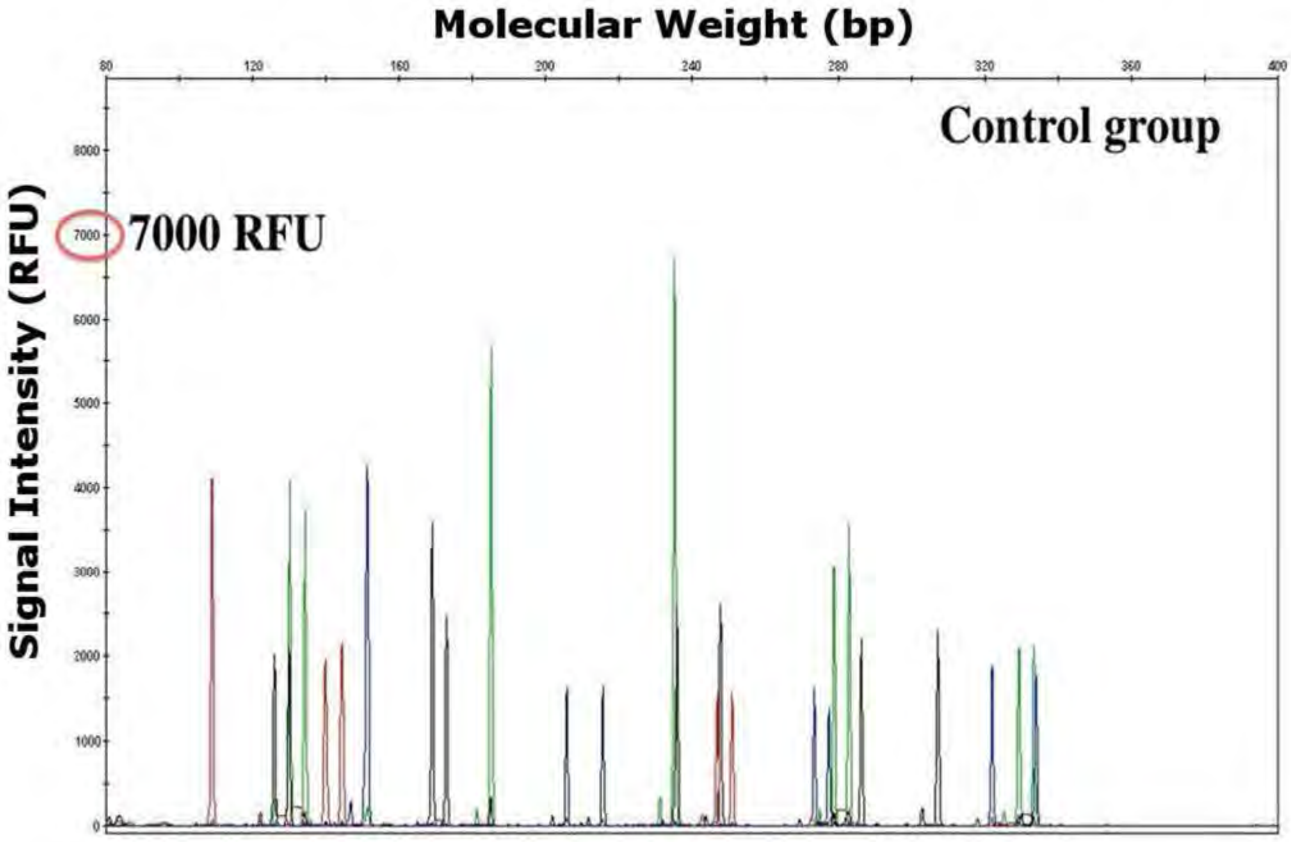

Fig. S3. Example of DNA profile obtained in a control group sample.

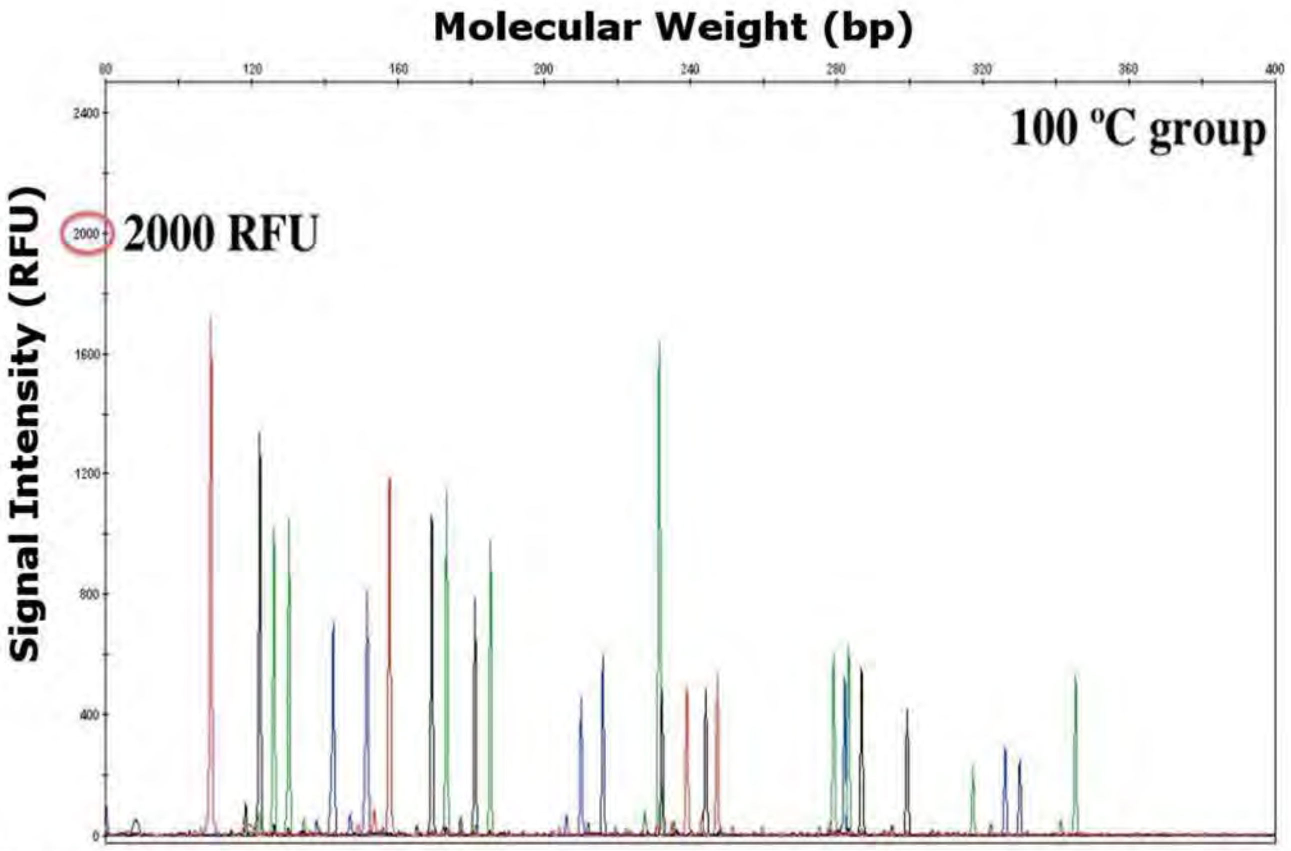

Fig. S4. Example of DNA profile obtained in one sample after incineration at 100 °C for 60 min.

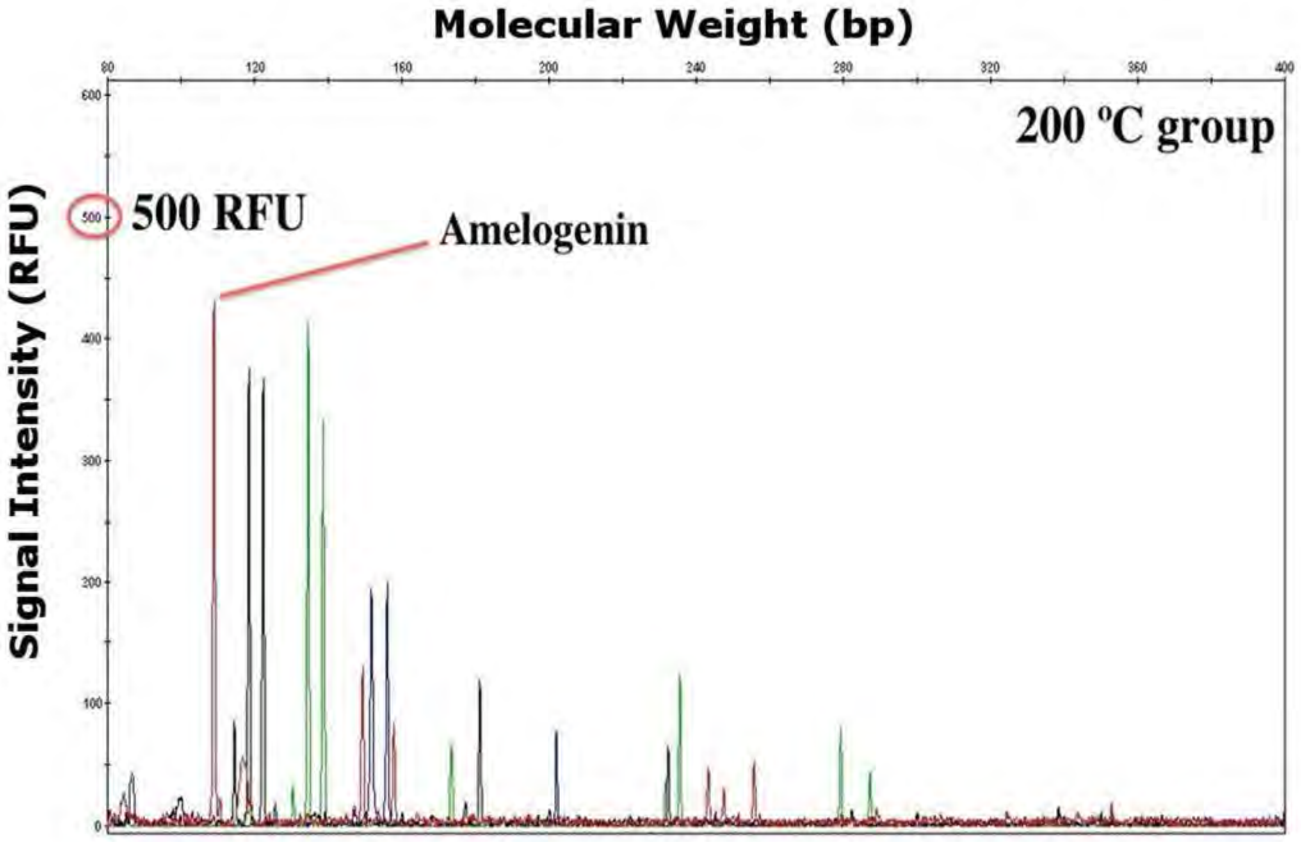

Fig. S5. Example of DNA profile obtained after incineration at 200 °C for 60 min.

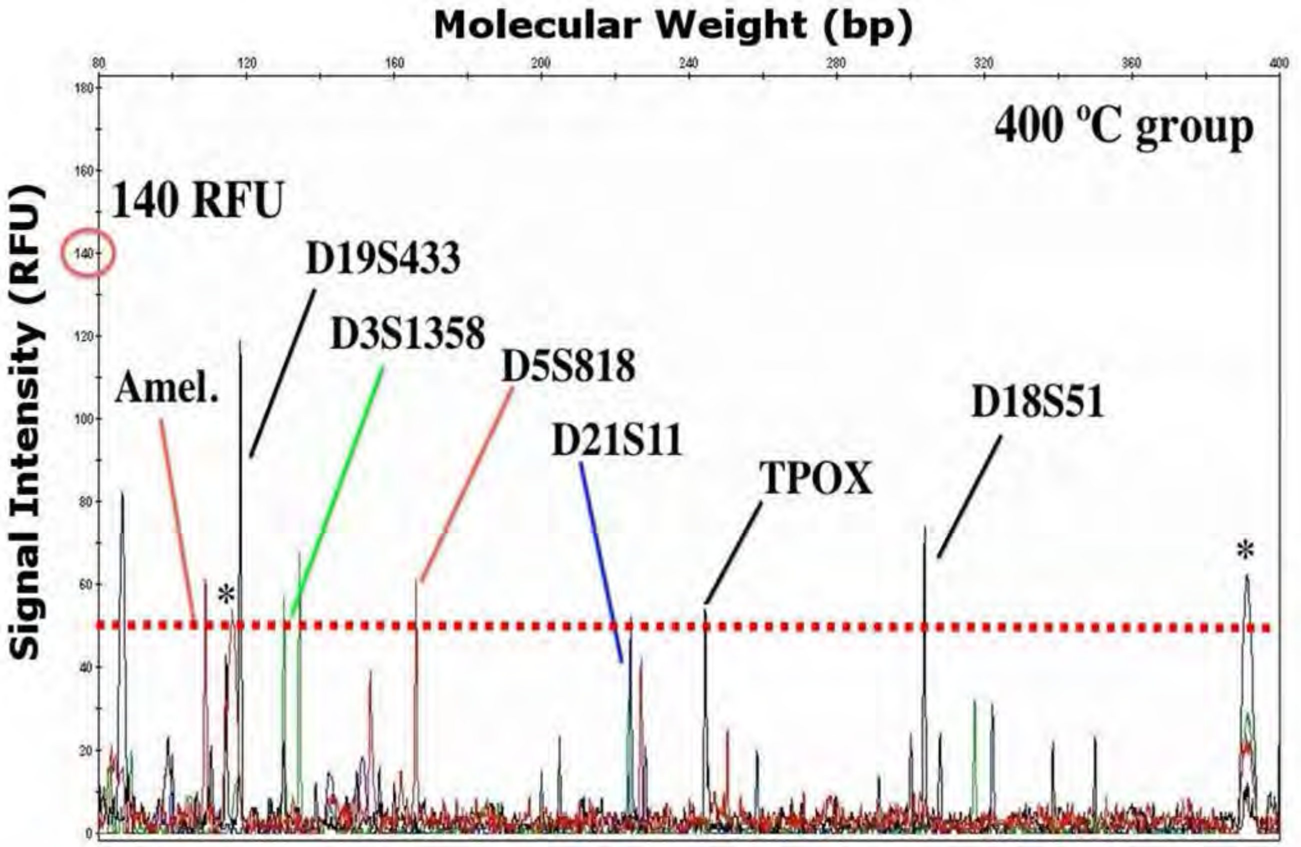

Fig. S6. DNA profile obtained after incineration at 400 °C for 60 min. Artefact peaks are marked by asterisks (*). Threshold for allele calling (50 RFU) is depicted by dotted red line.

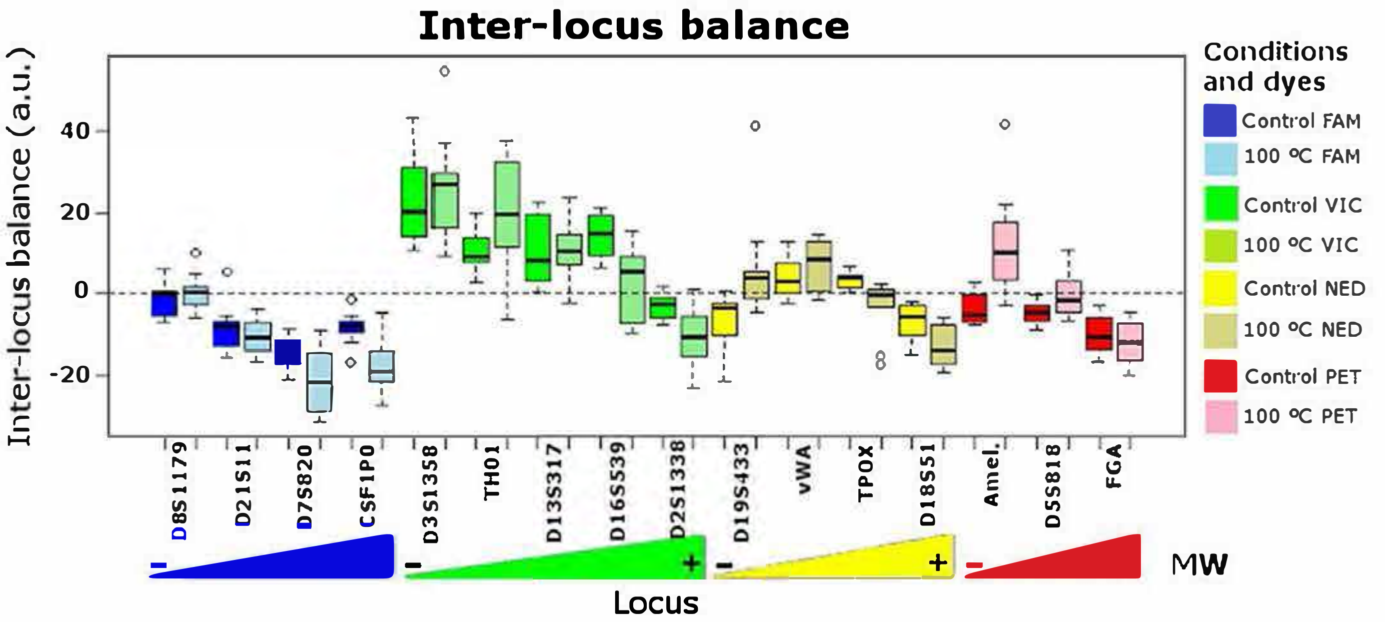

Fig. S7. Inter-locus balance in arbitrary units (a.u.) is depicted by dye color from smallest to largest molecular weight (MW) amplicons. For each dye group (FAM, VIC, NED and PET), a.u values of each locus are shown for control (bright color) and incinerated (light color) conditions. (Created using R Core Team, 2013 with package ggplot2)[38]

Assay Class: High Sensitivity DNA Assay
Data Path: C:\...gh Sensitivity DNA Assay_DE04105532_2014-12-15_12-24-38.xad

Created: 12/15/2014 12:26:15 PM
Modified: 12/15/2014 1:05:37 PM

Electrophoresis File Run Summary

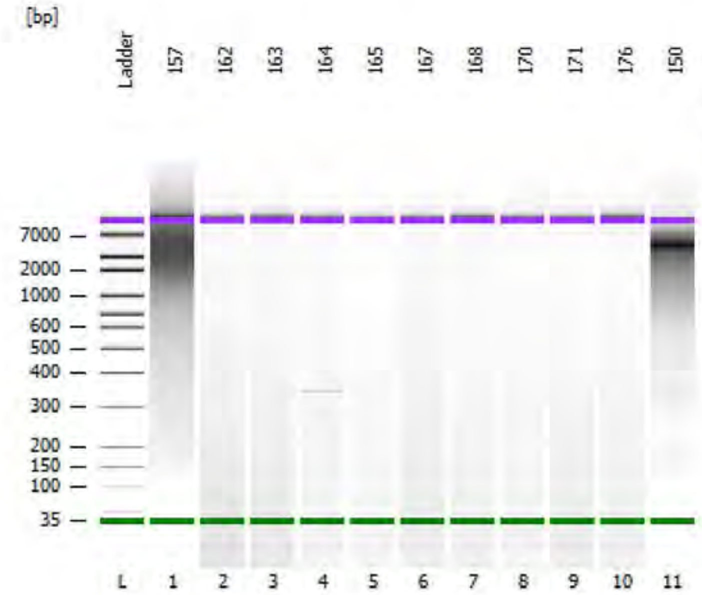

Instrument Information:

Instrument Name: DE04105532

Serial#: DE04105532

Firmware: C.01.069

Type: G2938C

Assay Information:

Assay Origin Path: C:\Program Files\Agilent\2100 bioanalyzer\2100 expert\assays\dsDNA\High Sensitivity DNA.xsy

Assay Class: High Sensitivity DNA Assay

Version:

157

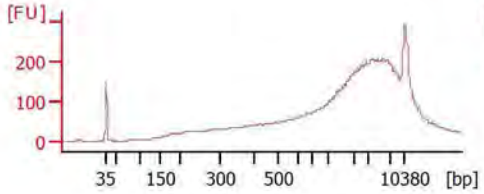

162

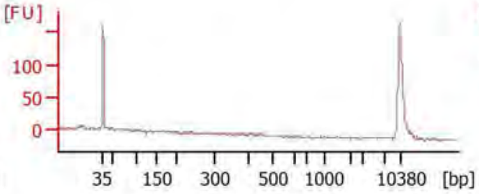

163

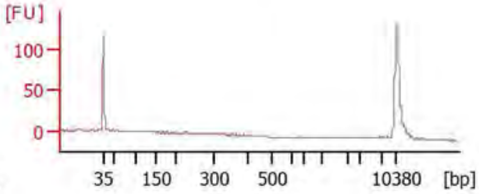

164

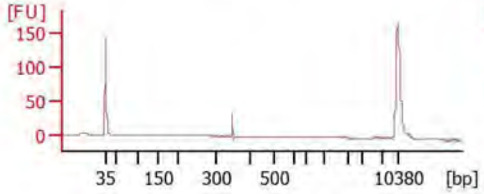

165

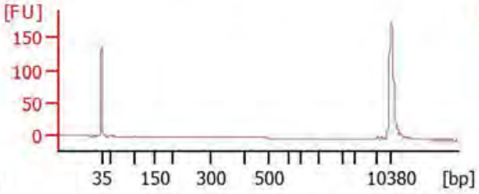

167

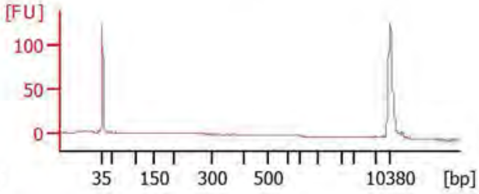

168

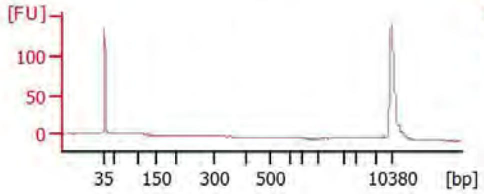

170

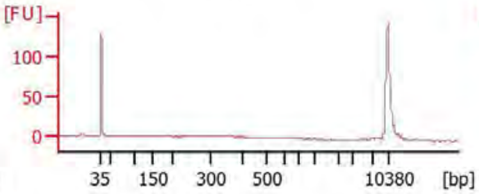

171

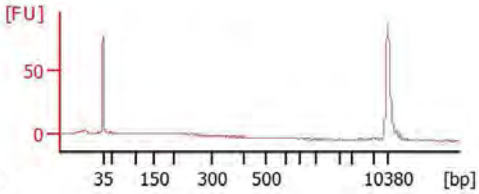

176

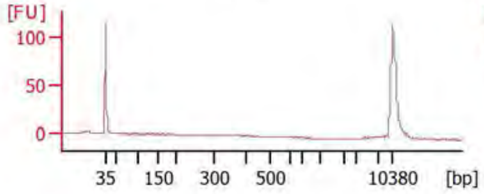

150

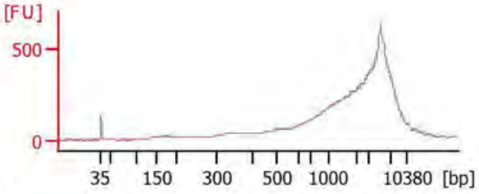

Assay Class: High Sensitivity DNA Assay
Data Path: C:\...gh Sensitivity DNA Assay_DE04105532_2014-12-15_12-24-38.xad

Created: 12/15/2014 12:26:15 PM
Modified: 12/15/2014 1:05:37 PM

Electrophoresis File Run Summary (Chip Summary)

Sample Name	Sample Comment	Rest. Digest	Status	Observation	Result Label	Result Color
157		☐	✔			
162		☐	✔			
163		☐	✔			
164		☐	✔			
165		☐	✔			
167		☐	✔			
168		☐	✔			
170		☐	✔			
171		☐	✔			
176		☐	✔			
150		☐	✔			
Ladder		☐	✔			

Chip Lot # **Reagent Kit Lot #**

Chip Comments :

Assay Class: High Sensitivity DNA Assay
Data Path: C:\...gh Sensitivity DNA Assay_DE04105532_2014-12-15_12-24-38.xad

Created: 12/15/2014 12:26:15 PM
Modified: 12/15/2014 1:05:37 PM

Electrophoresis Assay Details

General Analysis Settings

Number of Available Sample and Ladder Wells (Max.) : 12
Minimum Visible Range [s] : 32
Maximum Visible Range [s] : 138
Start Analysis Time Range [s] : 33
End Analysis Time Range [s] : 137.5
Ladder Concentration [pg/µl] : 1950
Uses Standard Area for Ladder Fragments
Lower Marker Concentration [pg/µl] : 125
Upper Marker Concentration [pg/µl] : 75
Used Upper Marker for Quantitation
Standard Curve Fit is Point to Point
Show Data Aligned to Lower and Upper Marker

Integrator Settings

Integration Start Time [s] : 33.05
Integration End Time [s] : 137
Slope Threshold : 0.8
Height Threshold [FU] : 5
Area Threshold : 0.1
Width Threshold [s] : 0.6
Baseline Plateau [s] : 0.5

Filter Settings

Filter Width [s] : 0.5
Polynomial Order : 4

Ladder

Ladder Peak	Size	Area
1	35	160
2	50	210
3	100	208
4	150	221
5	200	242
6	300	270
7	400	305
8	500	306
9	600	336
10	700	321
11	1000	366
12	2000	413
13	3000	411
14	7000	400
15	10380	214

Assay Class:	High Sensitivity DNA Assay	Created:	12/15/2014 12:26:15 PM
Data Path:	C:\...gh Sensitivity DNA Assay_DE04105532_2014-12-15_12-24-38.xad	Modified:	12/15/2014 1:05:37 PM

Electropherogram Summary

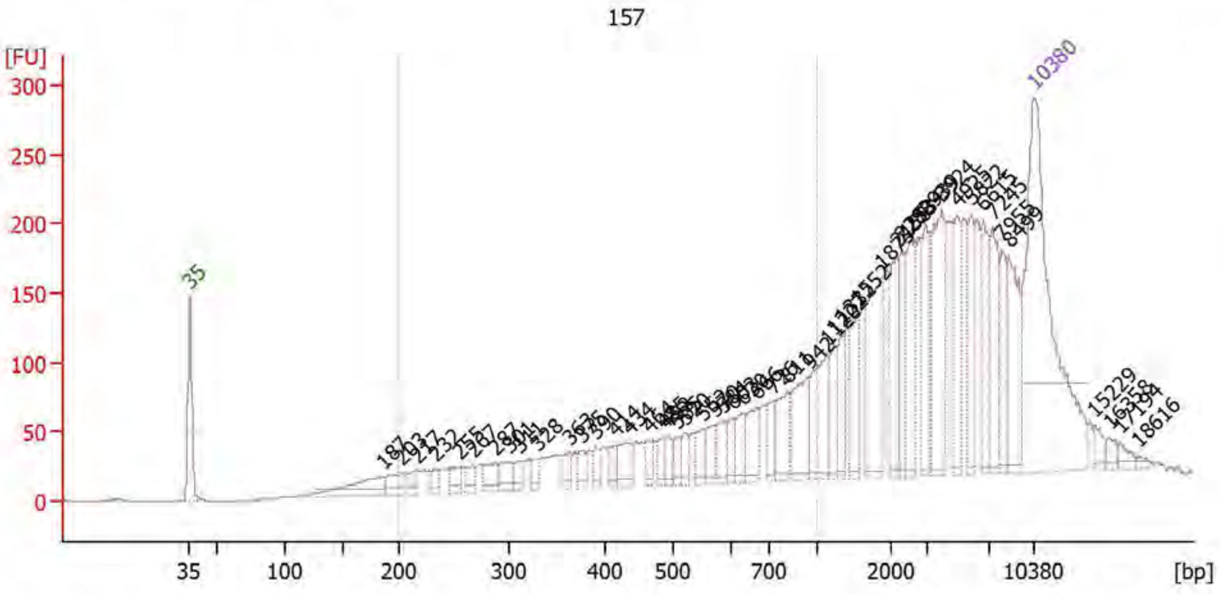

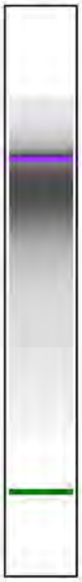

Overall Results for sample 1 : 157

Number of peaks found:	49	Corr. Area 1:	1,506.1
Noise:	0.4		

Peak table for sample 1 : 157

Peak	Size [bp]	Conc. [pg/µl]	Molarity [pmol/l]	Observations
1	35	125.00	5,411.3	Lower Marker
2	187	19.70	159.8	
3	203	8.70	64.9	
4	217	3.85	26.9	
5	232	3.85	25.1	
6	255	5.85	34.8	
7	267	5.07	28.8	
8	287	8.06	42.6	
9	301	7.21	36.3	
10	311	5.14	25.1	
11	328	4.13	19.0	
12	363	5.39	22.5	
13	375	4.69	18.9	
14	390	4.17	16.2	
15	414	4.56	16.7	
16	434	9.38	32.8	
17	464	4.76	15.5	
18	485	4.57	14.3	
19	492	4.97	15.3	
20	505	4.93	14.8	
21	520	4.67	13.6	
22	552	6.14	16.9	
23	570	7.44	19.8	
24	584	7.00	18.2	
25	603	6.41	16.1	
26	630	7.23	17.4	

Assay Class: High Sensitivity DNA Assay
Data Path: C:\...gh Sensitivity DNA Assay_DE04105532_2014-12-15_12-24-38.xad

Created: 12/15/2014 12:26:15 PM
Modified: 12/15/2014 1:05:37 PM

Electropherogram Summary Continued ...

... Peak table for sample 1 : 157

Peak	Size [bp]	Conc. [pg/µl]	Molarity [pmol/l]	Observations
27	666	10.78	24.5	
28	726	7.96	16.6	
29	811	12.83	24.0	
30	942	18.03	29.0	
31	1,120	20.41	27.6	
32	1,222	9.48	11.7	
33	1,332	8.70	9.9	
34	1,552	12.98	12.7	
35	1,875	25.11	20.3	
36	2,158	15.30	10.7	
37	2,288	12.10	8.0	
38	2,534	16.32	9.8	
39	2,939	14.05	7.2	
40	3,924	29.89	11.5	
41	4,925	14.47	4.5	
42	5,822	13.32	3.5	
43	6,615	16.74	3.8	
44	7,245	17.53	3.7	
45	7,955	11.02	2.1	
46	8,499	20.01	3.6	
47	10,380	75.00	10.9	Upper Marker
48	15,229	0.00	0.0	
49	16,358	0.00	0.0	
50	17,194	0.00	0.0	
51	18,616	0.00	0.0	

Region table for sample 1 : 157

From [bp]	To [bp]	Corr. Area	% of Total	Average Size [bp]	Size distribution in CV [%]	Conc. [pg/µl]	Molarity [pmol/l]	Color
200	1,000	1,506.1	35	551	39.0	260.91	929.6	

Assay Class: High Sensitivity DNA Assay
Data Path: C:\...gh Sensitivity DNA Assay_DE04105532_2014-12-15_12-24-38.xad

Created: 12/15/2014 12:26:15 PM
Modified: 12/15/2014 1:05:37 PM

Electropherogram Summary Continued ...

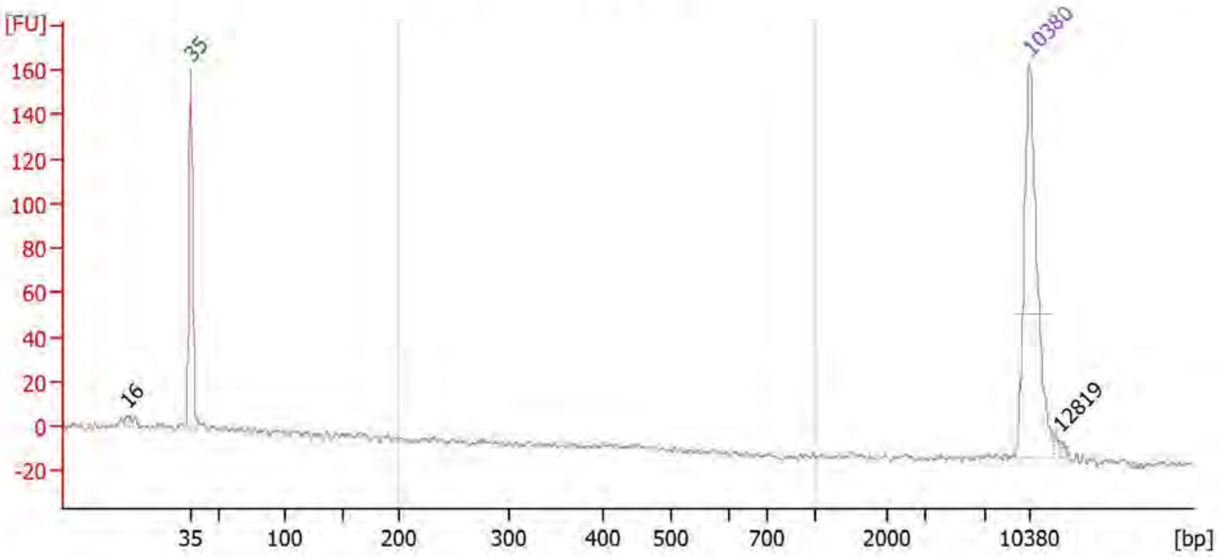

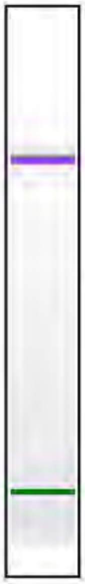

Overall Results for sample 2 : 162

Number of peaks found:	2	Corr. Area 1:	0.4
Noise:	1.3		

Peak table for sample 2 : 162

Peak		Size [bp]	Conc. [pg/µl]	Molarity [pmol/l]	Observations
1		16	0.00	0.0	
2	◄	35	125.00	5,411.3	Lower Marker
3	►	10,380	75.00	10.9	Upper Marker
4		12,819	0.00	0.0	

Region table for sample 2 : 162

From [bp]	To [bp]	Corr. Area	% of Total	Average Size [bp]	Size distribution in CV [%]	Conc. [pg/µl]	Molarity [pmol/l]	Color
200	1,000	0.4	1	343	28.7	0.23	1.2	

Assay Class: High Sensitivity DNA Assay
Data Path: C:\...gh Sensitivity DNA Assay_DE04105532_2014-12-15_12-24-38.xad

Created: 12/15/2014 12:26:15 PM
Modified: 12/15/2014 1:05:37 PM

Electropherogram Summary Continued ...

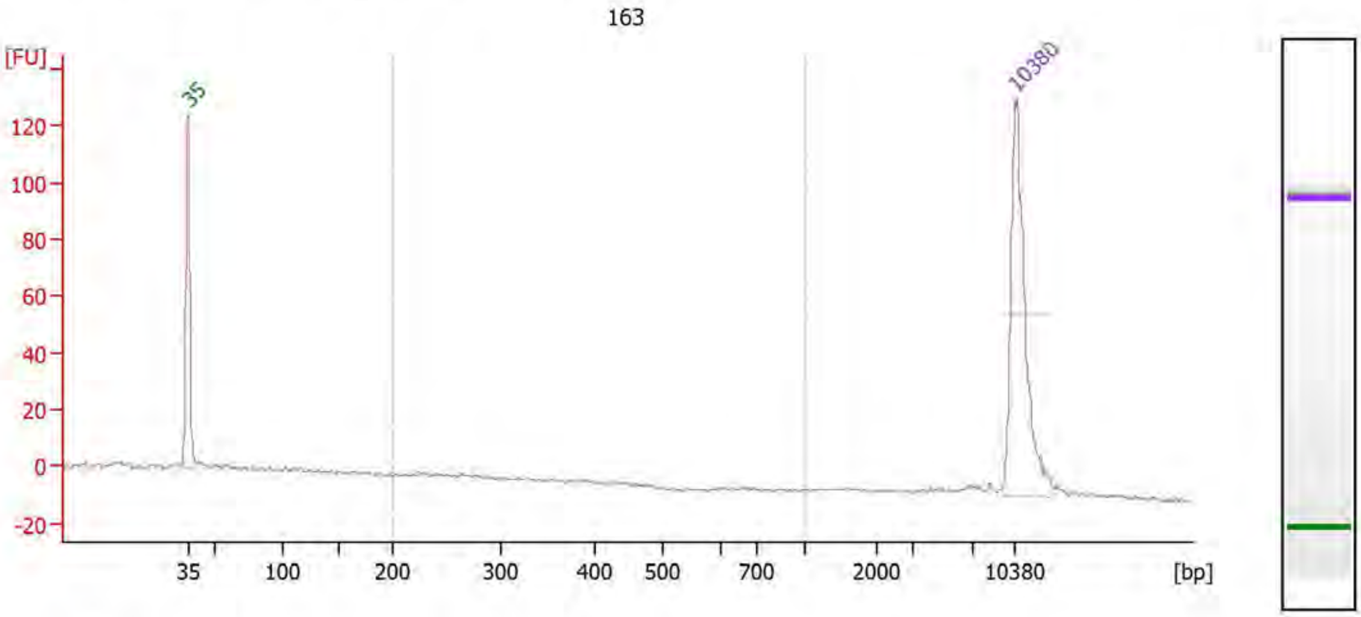

Overall Results for sample 3 : 163

Number of peaks found:	0	Corr. Area 1:	20.9
Noise:	0.4		

Peak table for sample 3 : 163

Peak	Size [bp]	Conc. [pg/µl]	Molarity [pmol/l]	Observations
1	35	125.00	5,411.3	Lower Marker
2	10,380	75.00	10.9	Upper Marker

Region table for sample 3 : 163

From [bp]	To [bp]	Corr. Area	% of Total	Average Size [bp]	Size distribution in CV [%]	Conc. [pg/µl]	Molarity [pmol/l]	Color
200	1,000	20.9	18	288	24.7	14.63	81.7	

Assay Class: High Sensitivity DNA Assay
Data Path: C:\...gh Sensitivity DNA Assay_DE04105532_2014-12-15_12-24-38.xad

Created: 12/15/2014 12:26:15 PM
Modified: 12/15/2014 1:05:37 PM

Electropherogram Summary Continued ...

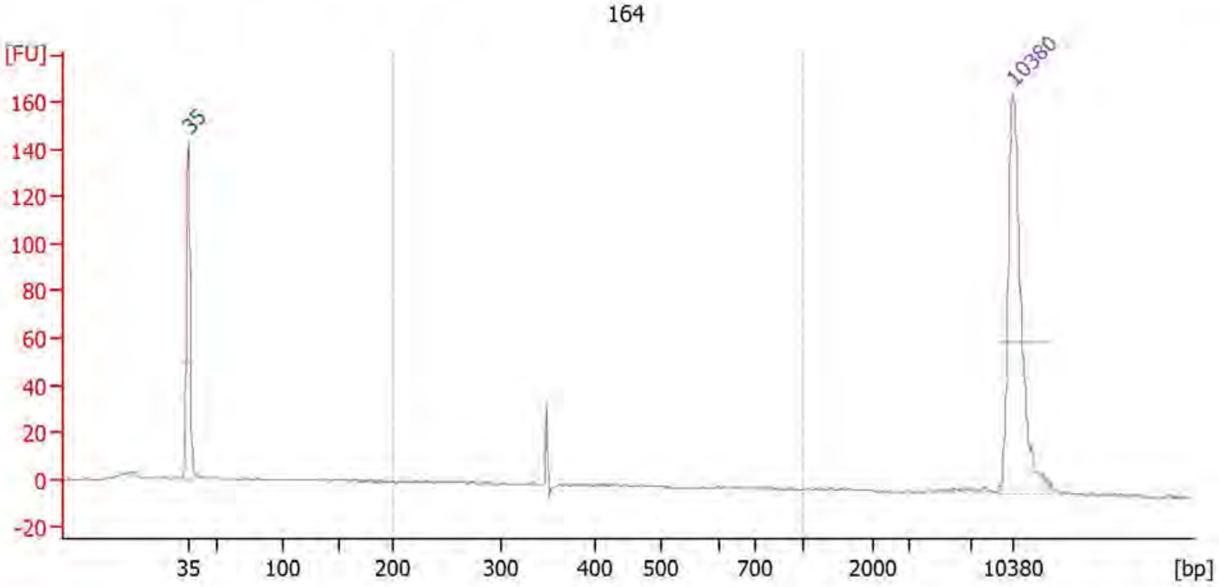

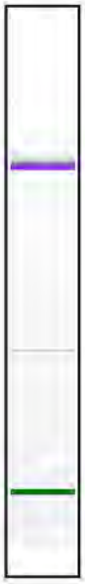

Overall Results for sample 4 : <u>164</u>

Number of peaks found:	0	Corr. Area 1:	66.3
Noise:	0.3		

Peak table for sample 4 : <u>164</u>

Peak		Size [bp]	Conc. [pg/µl]	Molarity [pmol/l]	Observations
1	◀	35	125.00	5,411.3	Lower Marker
2	▶	10,380	75.00	10.9	Upper Marker

Region table for sample 4 : <u>164</u>

From [bp]	To [bp]	Corr. Area	% of Total	Average Size [bp]	Size distribution in CV [%]	Conc. [pg/µl]	Molarity [pmol/l]	Color
200	1,000	66.3	40	422	41.2	38.00	167.1	

Assay Class: High Sensitivity DNA Assay
Data Path: C:\...gh Sensitivity DNA Assay_DE04105532_2014-12-15_12-24-38.xad

Created: 12/15/2014 12:26:15 PM
Modified: 12/15/2014 1:05:37 PM

Electropherogram Summary Continued ...

165

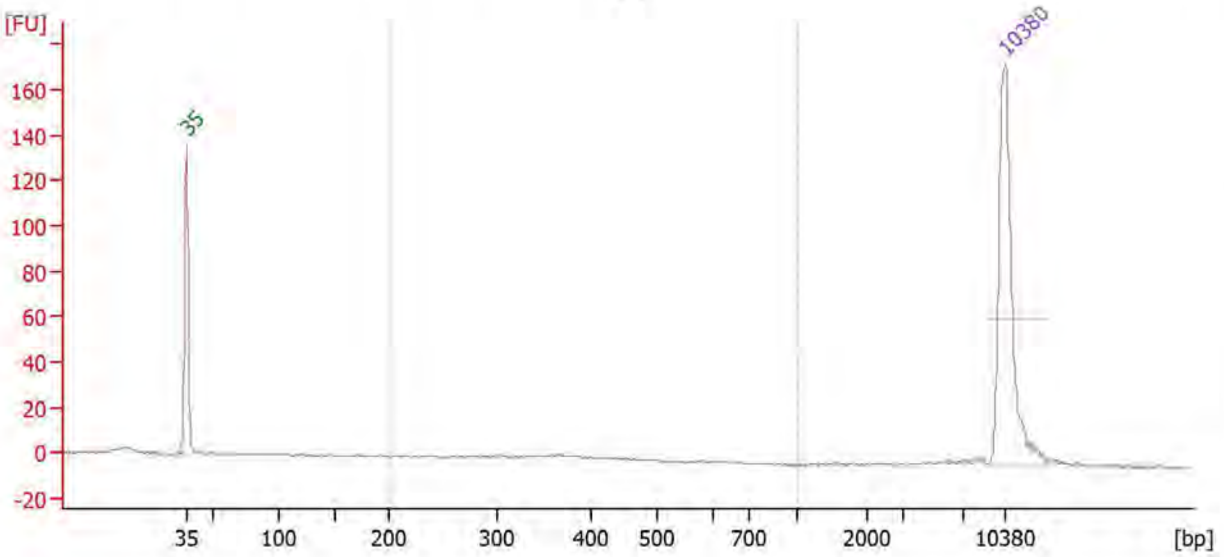

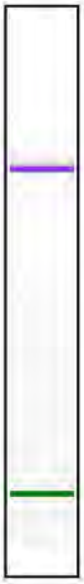

Overall Results for sample 5 : <u>165</u>

Number of peaks found:	0	Corr. Area 1:	26.2
Noise:	0.3		

Peak table for sample 5 : <u>165</u>

Peak		Size [bp]	Conc. [pg/µl]	Molarity [pmol/l]	Observations
1	◀	35	125.00	5,411.3	Lower Marker
2	▶	10,380	75.00	10.9	Upper Marker

Region table for sample 5 : <u>165</u>

From [bp]	To [bp]	Corr. Area	% of Total	Average Size [bp]	Size distribution in CV [%]	Conc. [pg/µl]	Molarity [pmol/l]	Color
200	1,000	26.2	35	348	18.7	14.25	65.4	

Assay Class: High Sensitivity DNA Assay
Data Path: C:\...gh Sensitivity DNA Assay_DE04105532_2014-12-15_12-24-38.xad
Created: 12/15/2014 12:26:15 PM
Modified: 12/15/2014 1:05:37 PM

Electropherogram Summary Continued ...

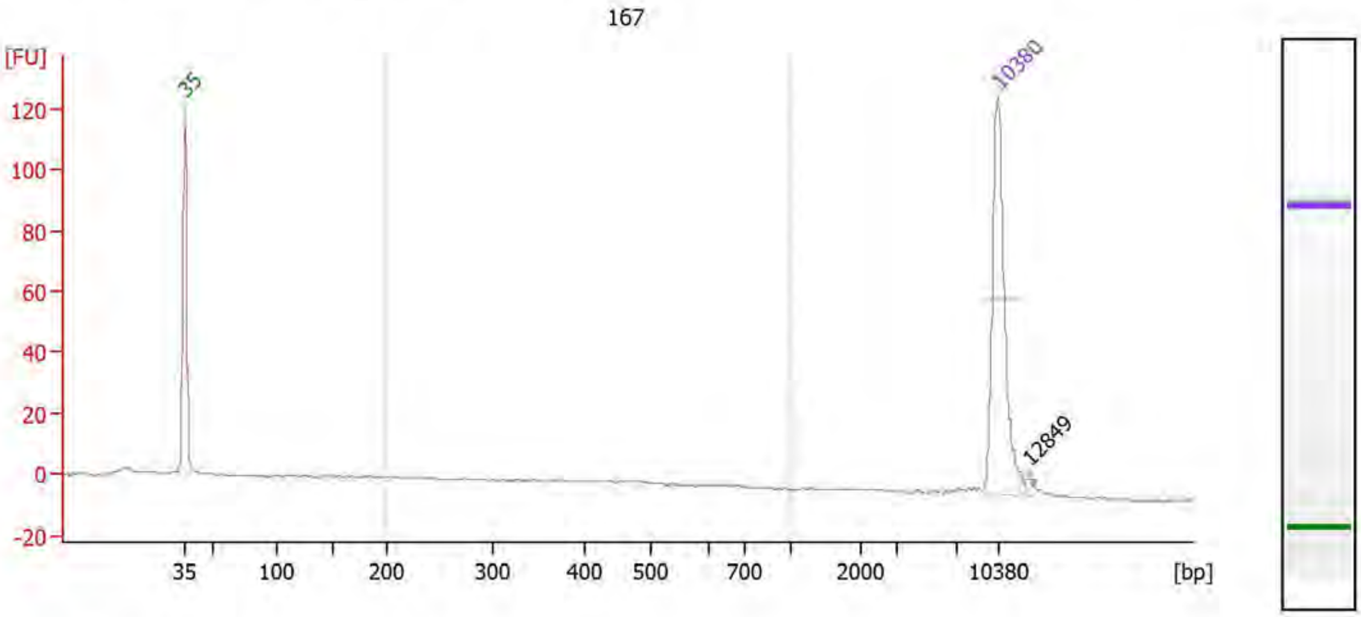

Overall Results for sample 6 : 167

Number of peaks found:	1	Corr. Area 1:	57.0
Noise:	0.2		

Peak table for sample 6 : 167

Peak		Size [bp]	Conc. [pg/µl]	Molarity [pmol/l]	Observations
1	◀	35	125.00	5,411.3	Lower Marker
2	▶	10,380	75.00	10.9	Upper Marker
3		12,849	0.00	0.0	

Region table for sample 6 : 167

From [bp]	To [bp]	Corr. Area	% of Total	Average Size [bp]	Size distribution in CV [%]	Conc. [pg/µl]	Molarity [pmol/l]	Color
200	1,000	57.0	41	426	39.0	44.60	195.2	■

Assay Class: High Sensitivity DNA Assay
Data Path: C:\...gh Sensitivity DNA Assay_DE04105532_2014-12-15_12-24-38.xad

Created: 12/15/2014 12:26:15 PM
Modified: 12/15/2014 1:05:37 PM

Electropherogram Summary Continued ...

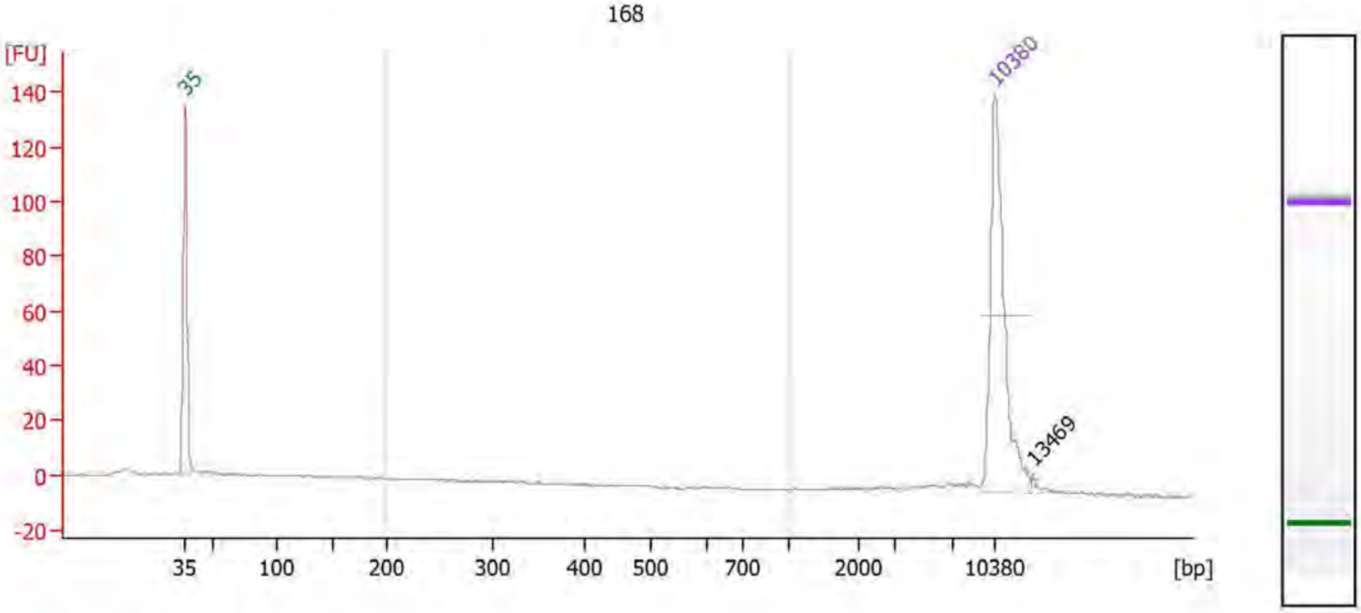

Overall Results for sample 7 : 168

Number of peaks found:	1	Corr. Area 1:	22.2
Noise:	0.3		

Peak table for sample 7 : 168

Peak		Size [bp]	Conc. [pg/µl]	Molarity [pmol/l]	Observations
1	◄	35	125.00	5,411.3	Lower Marker
2	►	10,380	75.00	10.9	Upper Marker
3		13,469	0.00	0.0	

Region table for sample 7 : 168

From [bp]	To [bp]	Corr. Area	% of Total	Average Size [bp]	Size distribution in CV [%]	Conc. [pg/µl]	Molarity [pmol/l]	Co lor
200	1,000	22.2	17	308	27.4	14.52	77.4	■

Assay Class: High Sensitivity DNA Assay
Data Path: C:\...gh Sensitivity DNA Assay_DE04105532_2014-12-15_12-24-38.xad

Created: 12/15/2014 12:26:15 PM
Modified: 12/15/2014 1:05:37 PM

Electropherogram Summary Continued ...

170

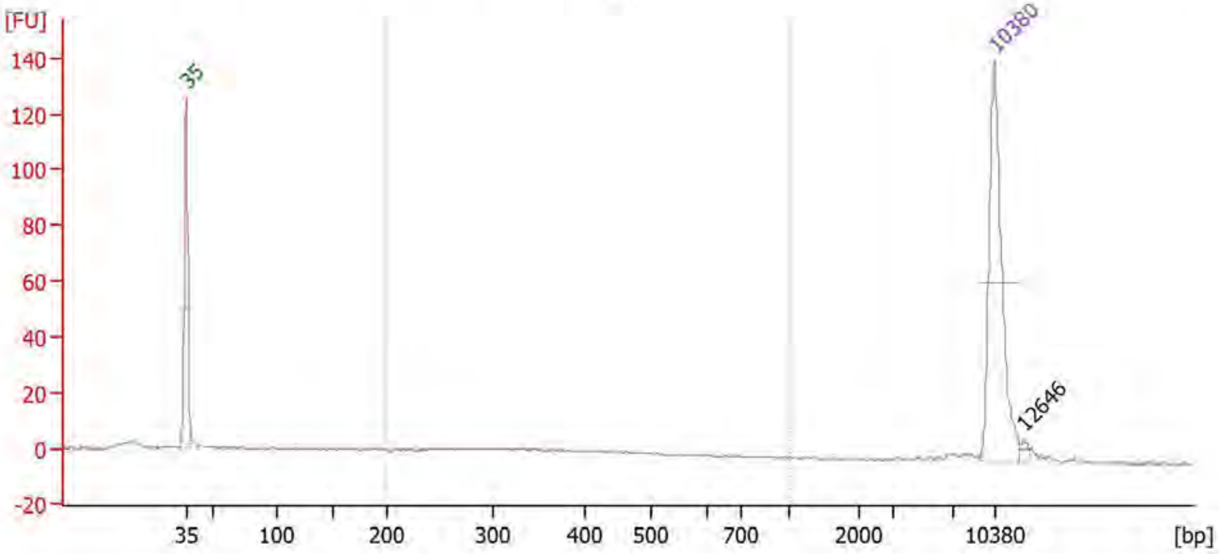

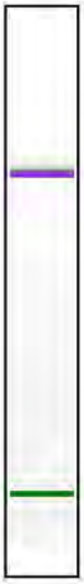

Overall Results for sample 8 : 170

Number of peaks found: 1
Corr. Area 1: 50.9
Noise: 0.3

Peak table for sample 8 : 170

Peak		Size [bp]	Conc. [pg/µl]	Molarity [pmol/l]	Observations
1	◀	35	125.00	5,411.3	Lower Marker
2	▶	10,380	75.00	10.9	Upper Marker
3		12,646	0.00	0.0	

Region table for sample 8 : 170

From [bp]	To [bp]	Corr. Area	% of Total	Average Size [bp]	Size distribution in CV [%]	Conc. [pg/µl]	Molarity [pmol/l]	Color
200	1,000	50.9	44	368	31.1	34.95	162.2	

Assay Class: High Sensitivity DNA Assay
Data Path: C:\...gh Sensitivity DNA Assay_DE04105532_2014-12-15_12-24-38.xad

Created: 12/15/2014 12:26:15 PM
Modified: 12/15/2014 1:05:37 PM

Electropherogram Summary Continued ...

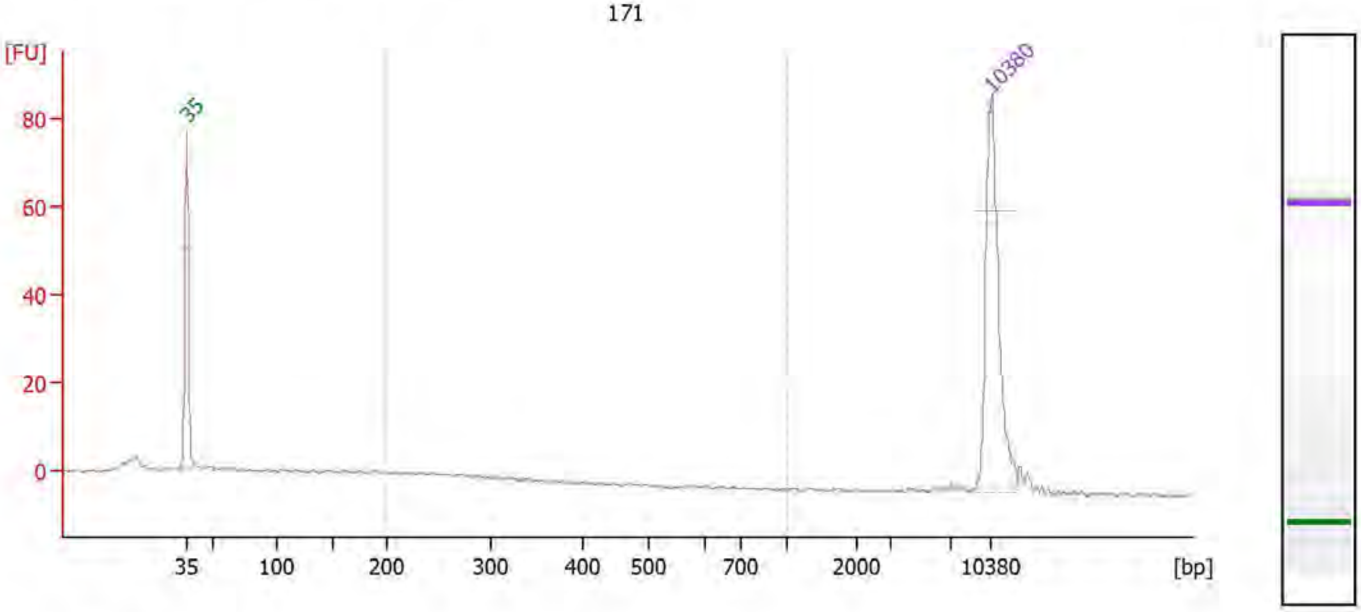

Overall Results for sample 9 : 171

Number of peaks found:	0	Corr. Area 1:	16.9
Noise:	0.3		

Peak table for sample 9 : 171

Peak		Size [bp]	Conc. [pg/µl]	Molarity [pmol/l]	Observations
1	◀	35	125.00	5,411.3	Lower Marker
2	▶	10,380	75.00	10.9	Upper Marker

Region table for sample 9 : 171

From [bp]	To [bp]	Corr. Area	% of Total	Average Size [bp]	Size distribution in CV [%]	Conc. [pg/µl]	Molarity [pmol/l]	Color
200	1,000	16.9	19	257	15.8	19.62	117.7	■

Assay Class: High Sensitivity DNA Assay
Data Path: C:\...gh Sensitivity DNA Assay_DE04105532_2014-12-15_12-24-38.xad
Created: 12/15/2014 12:26:15 PM
Modified: 12/15/2014 1:05:37 PM

Electropherogram Summary Continued ...

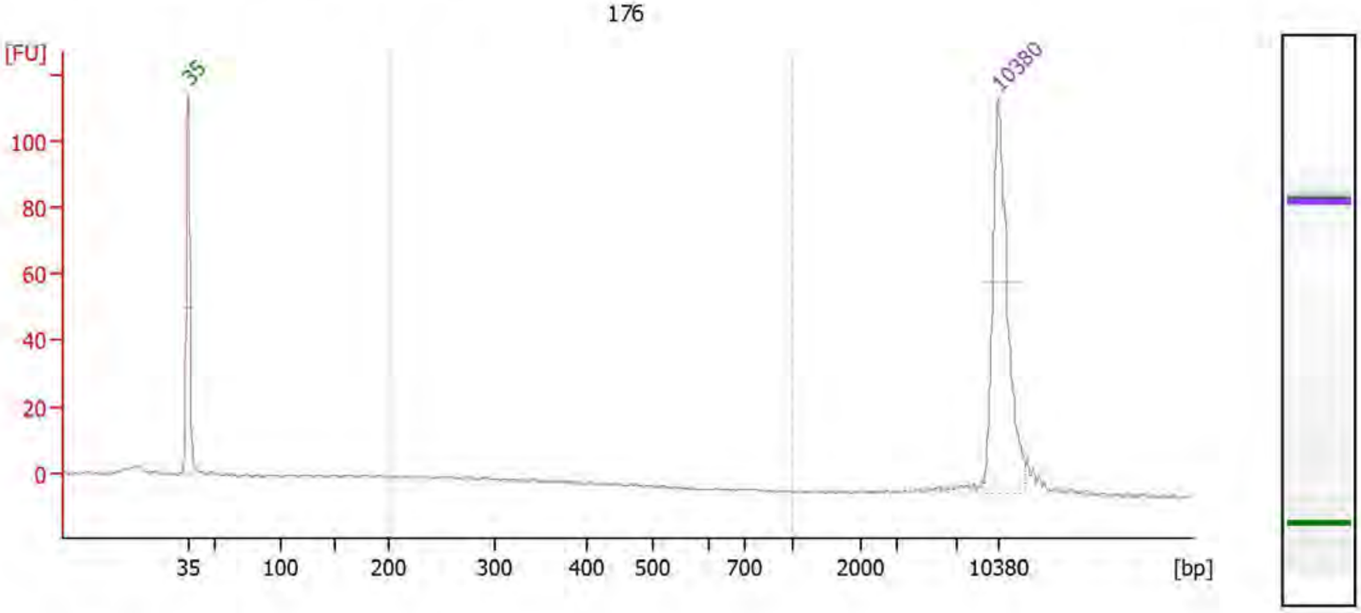

Overall Results for sample 10 : 176

Number of peaks found: 0   Corr. Area 1: 25.8
Noise: 0.3

Peak table for sample 10 : 176

Peak		Size [bp]	Conc. [pg/µl]	Molarity [pmol/l]	Observations
1	◀	35	125.00	5,411.3	Lower Marker
2	▶	10,380	75.00	10.9	Upper Marker

Region table for sample 10 : 176

From [bp]	To [bp]	Corr. Area	% of Total	Average Size [bp]	Size distribution in CV [%]	Conc. [pg/µl]	Molarity [pmol/l]	Co lor
200	1,000	25.8	26	298	22.6	19.32	104.2	■

Assay Class: High Sensitivity DNA Assay
Data Path: C:\...gh Sensitivity DNA Assay_DE04105532_2014-12-15_12-24-38.xad

Created: 12/15/2014 12:26:15 PM
Modified: 12/15/2014 1:05:37 PM

Electropherogram Summary Continued ...

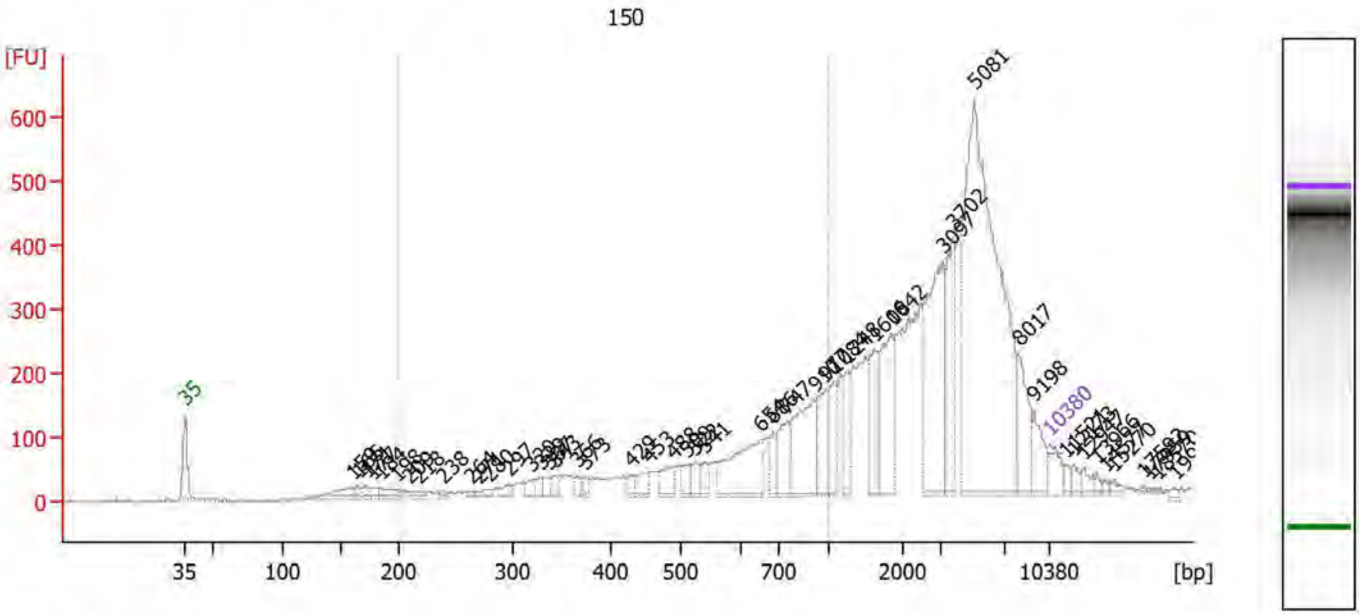

Overall Results for sample 11 : 150

Number of peaks found:	49	Corr. Area 1:	2,007.6
Noise:	1.2		

Peak table for sample 11 : 150

Peak	Size [bp]	Conc. [pg/µl]	Molarity [pmol/l]	Observations
1	35	125.00	5,411.3	Lower Marker
2	159	128.00	1,217.1	
3	165	35.21	323.5	
4	171	39.70	351.0	
5	177	36.46	311.3	
6	184	60.91	500.6	
7	198	24.21	185.5	
8	209	24.62	178.8	
9	218	18.86	131.1	
10	238	18.82	119.8	
11	264	13.75	78.9	
12	271	15.27	85.4	
13	280	27.42	148.4	
14	297	45.91	234.3	
15	320	41.23	195.4	
16	329	42.66	196.2	
17	337	36.22	162.8	
18	343	43.27	191.2	
19	366	34.33	142.2	
20	373	35.08	142.4	
21	429	37.83	133.5	
22	453	64.23	215.0	
23	488	94.52	293.7	
24	509	58.10	172.8	
25	522	66.42	192.8	
26	541	55.10	154.4	

Assay Class: High Sensitivity DNA Assay
Data Path: C:\...gh Sensitivity DNA Assay_DE04105532_2014-12-15_12-24-38.xad

Created: 12/15/2014 12:26:15 PM
Modified: 12/15/2014 1:05:37 PM

Electropherogram Summary Continued ...

... Peak table for sample 11 : 150

Peak		Size [bp]	Conc. [pg/µl]	Molarity [pmol/l]	Observations
27		654	366.13	848.6	
28		686	88.72	196.0	
29		747	162.72	330.1	
30		910	353.29	588.4	
31		977	180.69	280.3	
32		1,084	157.33	219.9	
33		1,248	121.38	147.3	
34		1,606	192.85	182.0	
35		1,842	331.82	273.0	
36		3,097	575.98	281.8	
37		3,702	280.34	114.7	
38		5,081	1,836.73	547.7	
39		8,017	185.28	35.0	
40		9,198	107.63	17.7	
41	▶	10,380	75.00	10.9	Upper Marker
42		11,521	0.00	0.0	
43		12,173	0.00	0.0	
44		12,947	0.00	0.0	
45		13,966	0.00	0.0	
46		14,577	0.00	0.0	
47		15,270	0.00	0.0	
48		17,592	0.00	0.0	
49		17,959	0.00	0.0	
50		18,570	0.00	0.0	
51		19,956	0.00	0.0	

Region table for sample 11 : 150

From [bp]	To [bp]	Corr. Area	% of Total	Average Size [bp]	Size distribution in CV [%]	Conc. [pg/µl]	Molarity [pmol/l]	Color
200	1,000	2,007.6	27	613	34.9	2,467.79	7,654.7	■

Assay Class:	High Sensitivity DNA Assay	Created:	12/15/2014 12:26:15 PM
Data Path:	C:\...gh Sensitivity DNA Assay_DE04105532_2014-12-15_12-24-38.xad	Modified:	12/15/2014 1:05:37 PM

Gel Image

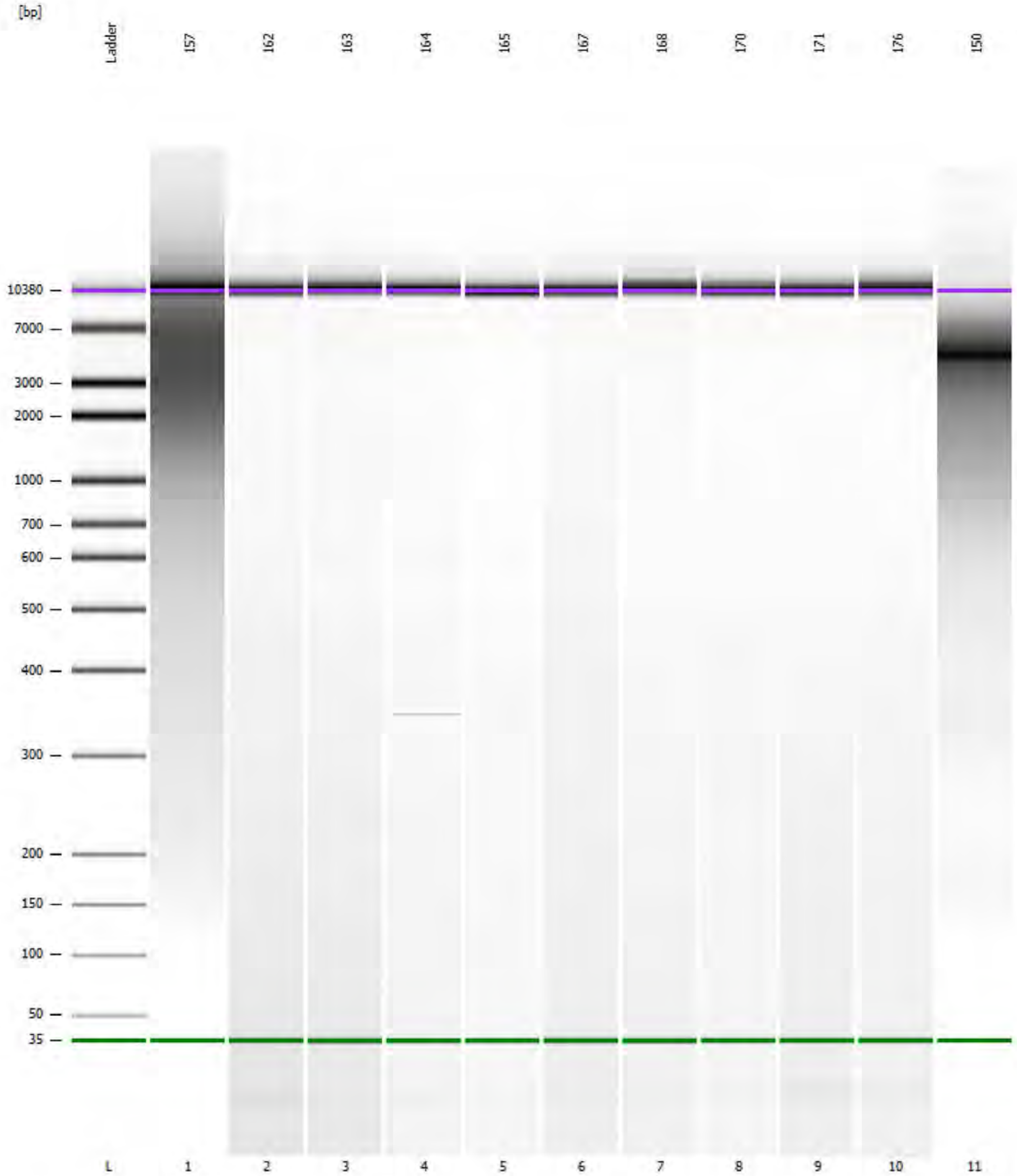

Assay Class: High Sensitivity DNA Assay
Data Path: C:\...gh Sensitivity DNA Assay_DE04105532_2014-12-15_12-24-38.xad

Created: 12/15/2014 12:26:15 PM
Modified: 12/15/2014 1:05:37 PM

Curves

Standard Curve

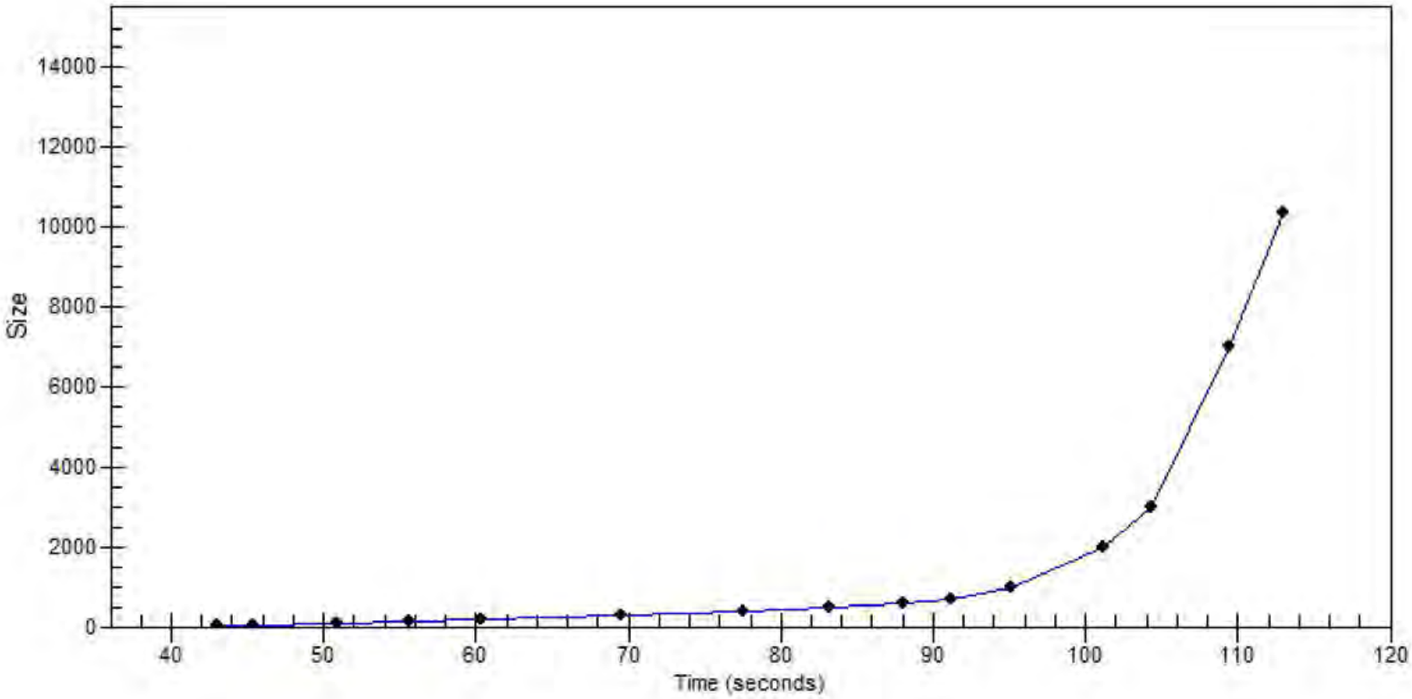

Assay Class: High Sensitivity DNA Assay
Data Path: C:\...gh Sensitivity DNA Assay_DE04105532_2014-12-15_12-24-38.xad

Created: 12/15/2014 12:26:15 PM
Modified: 12/15/2014 1:05:37 PM

Run Logbook

Description	Number	Source	Category	Sub Category	Time	Time Zone	User	Host
Run ended on port 2 (Number of wells acquired: 12)		Instrument	Run		12/15/2014 1:05:37 PM	(GMT +01:00) W. Europe Standard Time	Admin	Datasystem01
Run started on port 2 (File: C:\Program Files\Agilent\2100 bioanalyzer\2100 expert\Data\2014-12-15\2100 expert_High Sensitivity DNA Assay_DE04105532_2014-12-15_12-24-38.xad)		Instrument	Run		12/15/2014 12:26:15 PM	(GMT +01:00) W. Europe Standard Time	Admin	Datasystem01
Product Number : G2938C		Instrument	Run		12/15/2014 12:26:15 PM	(GMT +01:00) W. Europe Standard Time	Admin	Datasystem01
Name :		Instrument	Run		12/15/2014 12:26:15 PM	(GMT +01:00) W. Europe Standard Time	Admin	Datasystem01
Vendor : Agilent Technologies		Instrument	Run		12/15/2014 12:26:15 PM	(GMT +01:00) W. Europe Standard Time	Admin	Datasystem01
Serial# : DE04105532		Instrument	Run		12/15/2014 12:26:15 PM	(GMT +01:00) W. Europe Standard Time	Admin	Datasystem01
Firmware : C.01.069		Instrument	Run		12/15/2014 12:26:15 PM	(GMT +01:00) W. Europe Standard Time	Admin	Datasystem01
Cartridge : Electrode		Instrument	Run		12/15/2014 12:26:15 PM	(GMT +01:00) W. Europe Standard Time	Admin	Datasystem01

Assay Class: High Sensitivity DNA Assay
Data Path: C:\...gh Sensitivity DNA Assay_DE04105532_2014-12-15_14-01-58.xad

Created: 12/15/2014 2:01:57 PM
Modified: 12/15/2014 2:43:23 PM

Electrophoresis File Run Summary

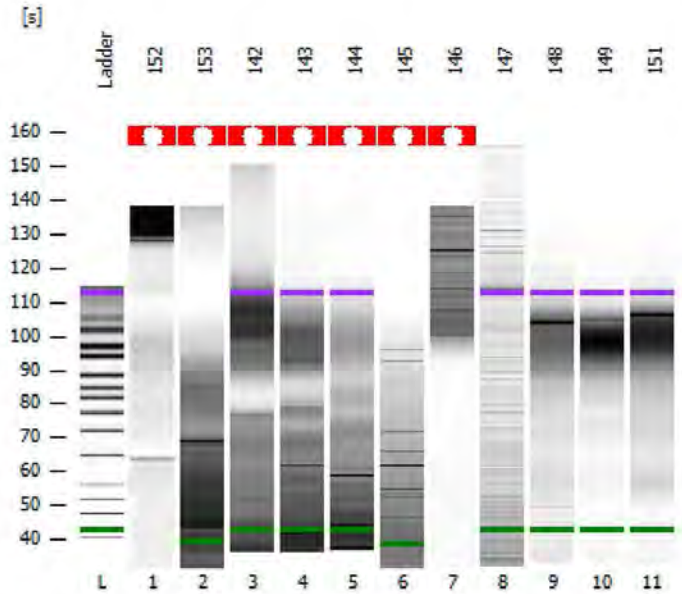

Instrument Information:

Instrument Name: DE04105532
Serial#: DE04105532
Firmware: C.01.069
Type: G2938C

Assay Information:

Assay Origin Path: C:\Program Files\Agilent\2100 bioanalyzer\2100 expert\assays\dsDNA\High Sensitivity DNA.xsy

Assay Class: High Sensitivity DNA Assay

Version: 1.03

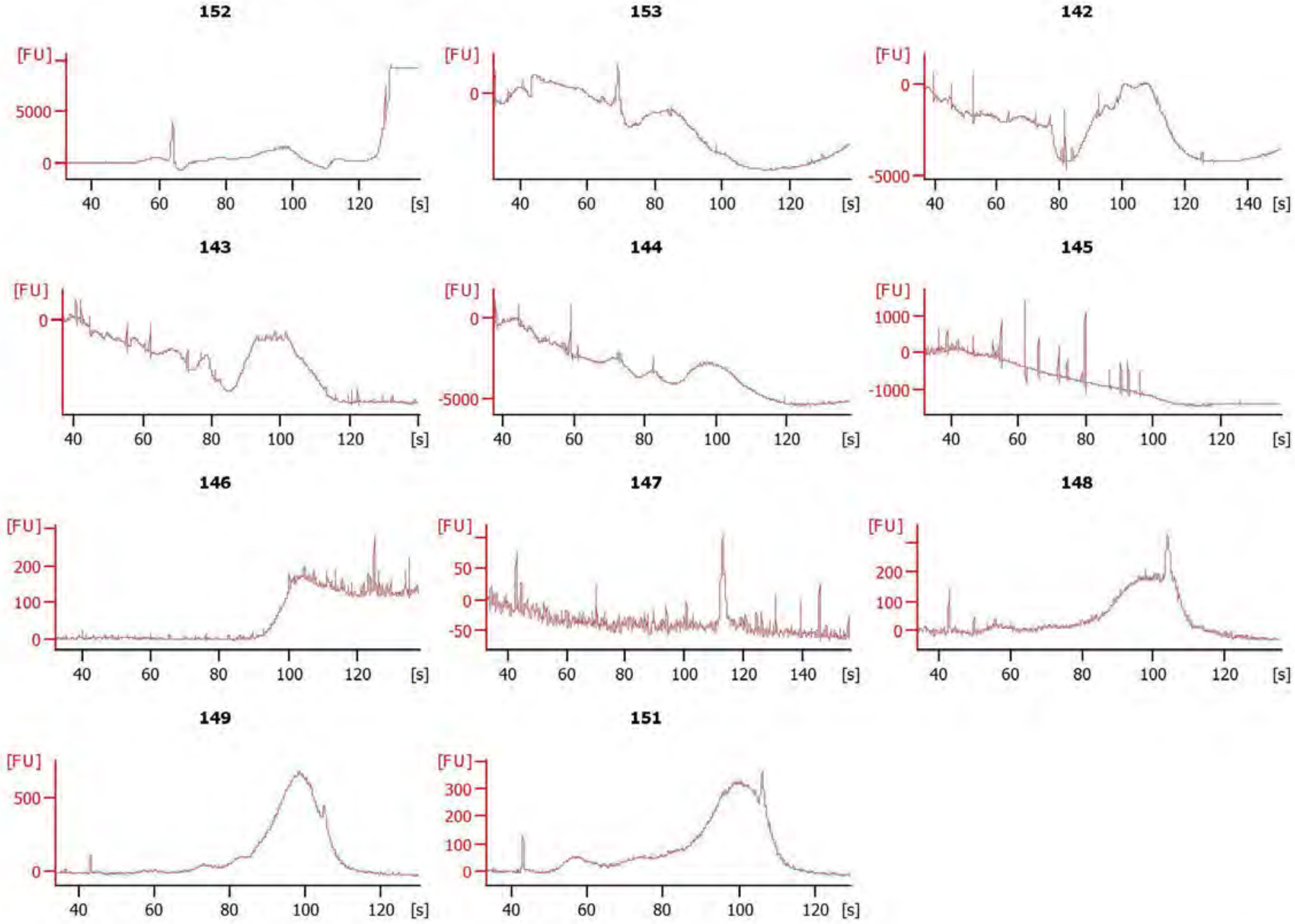

Assay Class: High Sensitivity DNA Assay
Data Path: C:\...gh Sensitivity DNA Assay_DE04105532_2014-12-15_14-01-58.xad
Created: 12/15/2014 2:01:57 PM
Modified: 12/15/2014 2:43:23 PM

Electrophoresis File Run Summary (Chip Summary)

Sample Name	Sample Comment	Rest. Digest	Status	Observation	Result Label	Result Color
152		☐	⚠			
153		☐	⚠			
142		☐	⚠			
143		☐	⚠			
144		☐	⚠			
145		☐	⚠			
146		☐	✔			
147		☐	✔			
148		☐	✔			
149		☐	✔			
151		☐	✔			
Ladder		☐	✔			

Chip Lot # **Reagent Kit Lot #**

Chip Comments :

Assay Class: High Sensitivity DNA Assay
Data Path: C:\...gh Sensitivity DNA Assay_DE04105532_2014-12-15_14-01-58.xad

Created: 12/15/2014 2:01:57 PM
Modified: 12/15/2014 2:43:23 PM

Electrophoresis Assay Details

General Analysis Settings

Number of Available Sample and Ladder Wells (Max.) : 12
Minimum Visible Range [s] : 32
Maximum Visible Range [s] : 138
Start Analysis Time Range [s] : 33
End Analysis Time Range [s] : 137.5
Ladder Concentration [pg/µl] : 1950
Uses Standard Area for Ladder Fragments
Lower Marker Concentration [pg/µl] : 125
Upper Marker Concentration [pg/µl] : 75
Used Upper Marker for Quantitation
Standard Curve Fit is Point to Point
Show Data Aligned to Lower and Upper Marker

Integrator Settings

Integration Start Time [s] : 33.05
Integration End Time [s] : 137
Slope Threshold : 0.8
Height Threshold [FU] : 5
Area Threshold : 0.1
Width Threshold [s] : 0.6
Baseline Plateau [s] : 0.5

Filter Settings

Filter Width [s] : 0.5
Polynomial Order : 4

Ladder

Ladder Peak	Size	Area
1	35	160
2	50	210
3	100	208
4	150	221
5	200	242
6	300	270
7	400	305
8	500	306
9	600	336
10	700	321
11	1000	366
12	2000	413
13	3000	411
14	7000	400
15	10380	214

Assay Class: High Sensitivity DNA Assay
Data Path: C:\...gh Sensitivity DNA Assay_DE04105532_2014-12-15_14-01-58.xad
Created: 12/15/2014 2:01:57 PM
Modified: 12/15/2014 2:43:23 PM

Electropherogram Summary

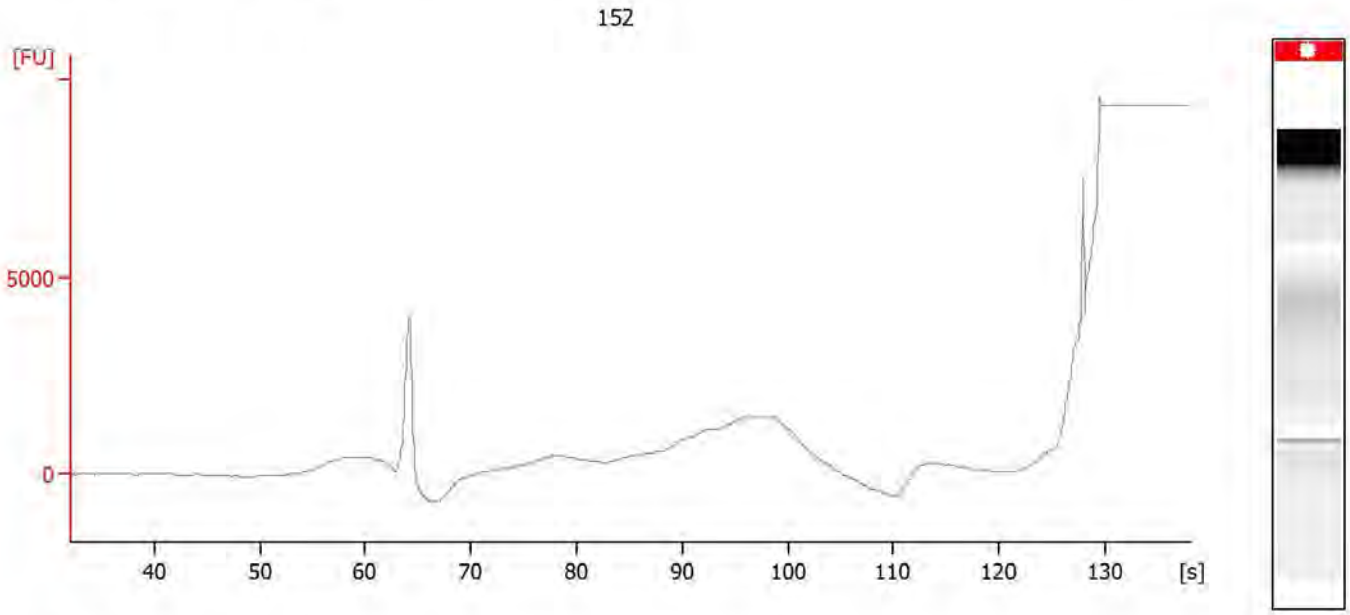

Overall Results for sample 1 : **152**

Noise: 14.2

Assay Class: High Sensitivity DNA Assay
Data Path: C:\...gh Sensitivity DNA Assay_DE04105532_2014-12-15_14-01-58.xad

Created: 12/15/2014 2:01:57 PM
Modified: 12/15/2014 2:43:23 PM

Electropherogram Summary Continued ...

153

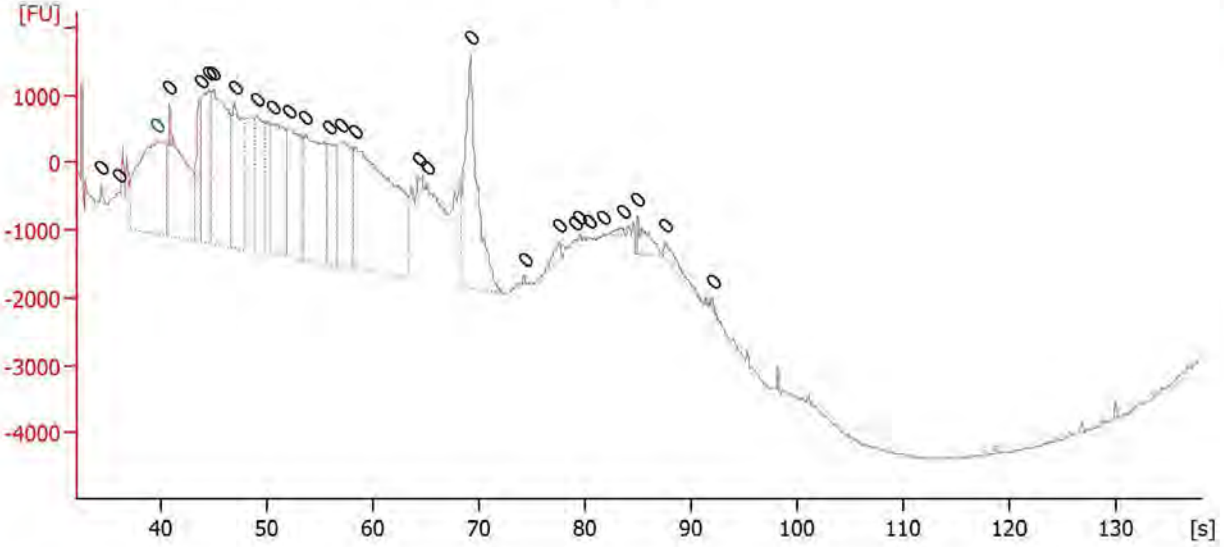

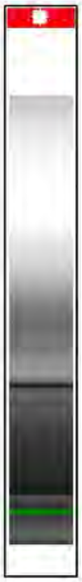

Overall Results for sample 2 : 153

Number of peaks found: 27 Noise: 7.3

Peak table for sample 2 : 153

Peak	Size [bp]	Conc. [pg/µl]	Molarity [pmol/l]	Observations
1	0	0.00	0.0	
2	0	0.00	0.0	
3	0	0.00	0.0	Lower Marker
4	0	0.00	0.0	
5	0	0.00	0.0	
6	0	0.00	0.0	
7	0	0.00	0.0	
8	0	0.00	0.0	
9	0	0.00	0.0	
10	0	0.00	0.0	
11	0	0.00	0.0	
12	0	0.00	0.0	
13	0	0.00	0.0	
14	0	0.00	0.0	
15	0	0.00	0.0	
16	0	0.00	0.0	
17	0	0.00	0.0	
18	0	0.00	0.0	
19	0	0.00	0.0	
20	0	0.00	0.0	
21	0	0.00	0.0	
22	0	0.00	0.0	
23	0	0.00	0.0	
24	0	0.00	0.0	
25	0	0.00	0.0	
26	0	0.00	0.0	
27	0	0.00	0.0	

Assay Class: High Sensitivity DNA Assay
Data Path: C:\...gh Sensitivity DNA Assay_DE04105532_2014-12-15_14-01-58.xad
Created: 12/15/2014 2:01:57 PM
Modified: 12/15/2014 2:43:23 PM

Electropherogram Summary Continued ...

... Peak table for sample 2 : 153

Peak	Size [bp]	Conc. [pg/µl]	Molarity [pmol/l]	Observations
28	0	0.00	0.0	

Assay Class: High Sensitivity DNA Assay
Data Path: C:\...gh Sensitivity DNA Assay_DE04105532_2014-12-15_14-01-58.xad

Created: 12/15/2014 2:01:57 PM
Modified: 12/15/2014 2:43:23 PM

Electropherogram Summary Continued ...

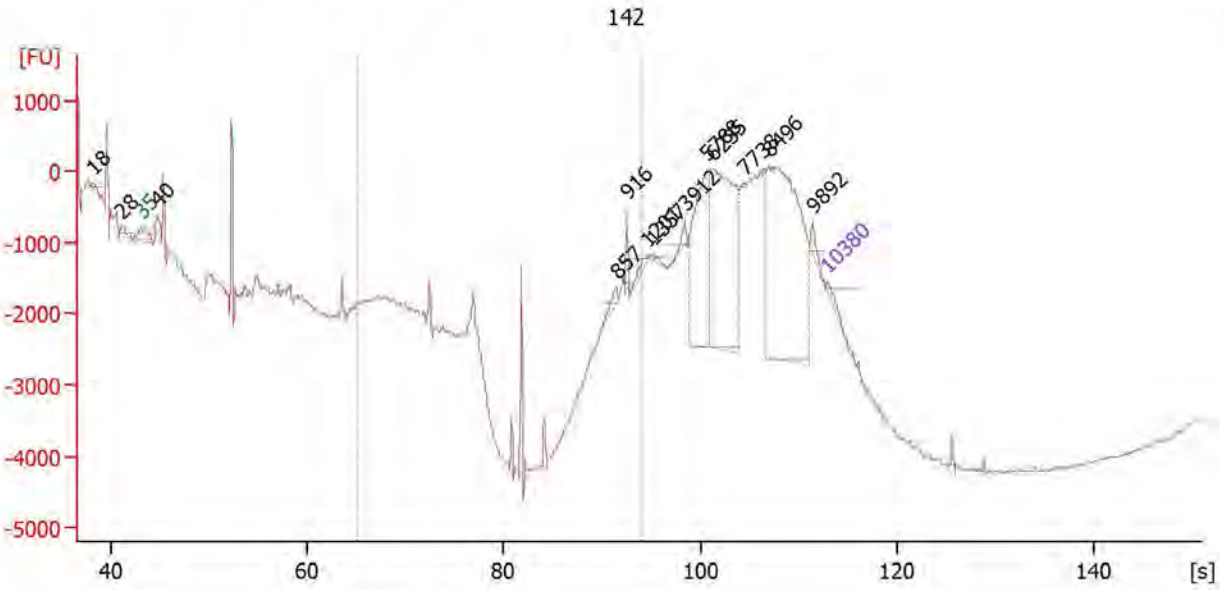

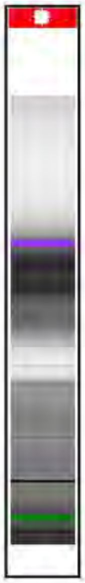

Overall Results for sample 3 : 142

Number of peaks found:	13	Corr. Area 1:	899.7
Noise:	34.5		

Peak table for sample 3 : 142

Peak		Size [bp]	Conc. [pg/µl]	Molarity [pmol/l]	Observations
1		18	0.00	0.0	
2		28	0.00	0.0	
3	◀	35	125.00	5,411.3	Lower Marker
4		40	169.09	6,400.2	
5		857	117.61	207.9	
6		916	120.56	199.4	
7		1,201	100.67	127.0	
8		1,357	24.34	27.2	
9		3,912	78.10	30.3	
10		5,788	1,922.24	503.2	
11		6,235	3,027.77	735.7	
12		7,738	12.38	2.4	
13		8,496	4,244.02	756.9	
14		9,892	77.19	11.8	
15	▶	10,380	75.00	10.9	Upper Marker

Region table for sample 3 : 142

From [bp]	To [bp]	Corr. Area	% of Total	Average Size [bp]	Size distribution in CV [%]	Conc. [pg/µl]	Molarity [pmol/l]	Co lor
200	1,000	899.7	3	933	6.3	425.12	687.2	

Assay Class: High Sensitivity DNA Assay
Data Path: C:\...gh Sensitivity DNA Assay_DE04105532_2014-12-15_14-01-58.xad

Created: 12/15/2014 2:01:57 PM
Modified: 12/15/2014 2:43:23 PM

Electropherogram Summary Continued ...

143

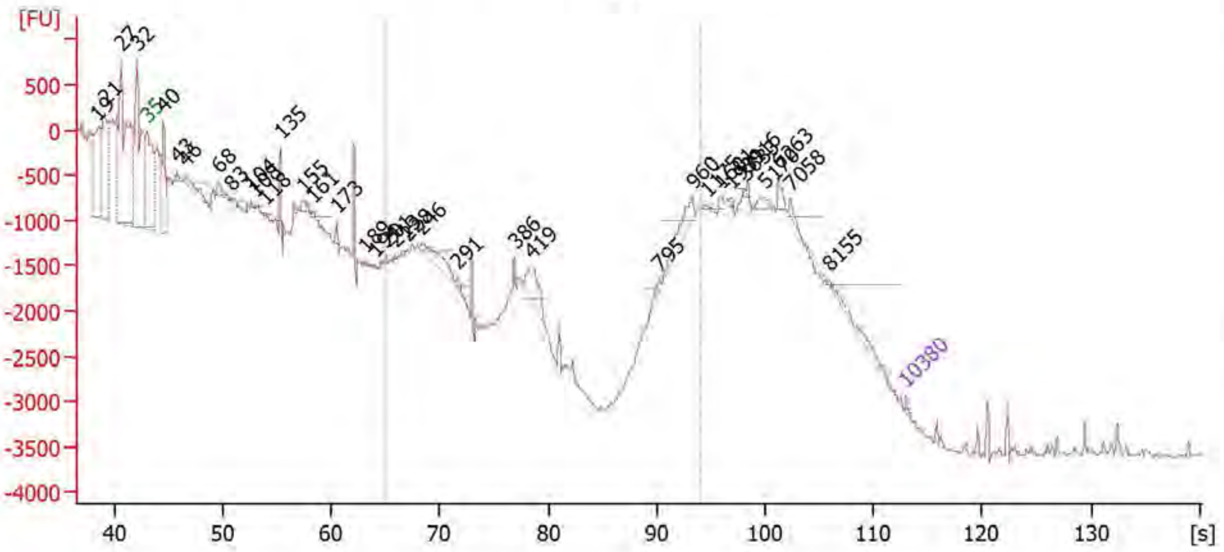

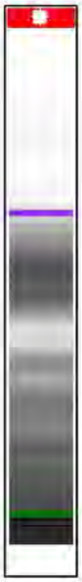

Overall Results for sample 4 : 143

Number of peaks found: 36

Corr. Area 1: 3,535.9

Noise: 32.4

Peak table for sample 4 : 143

Peak		Size [bp]	Conc. [pg/µl]	Molarity [pmol/l]	Observations
1		19	0.00	0.0	
2		21	0.00	0.0	
3		27	0.00	0.0	
4		32	0.00	0.0	
5	◀	35	125.00	5,411.3	Lower Marker
6		40	5,766.23	220,049.9	
7		43	479.29	16,798.1	
8		46	1,126.49	37,324.1	
9		68	758.17	16,835.2	
10		83	127.37	2,323.1	
11		104	284.86	4,142.2	
12		108	147.53	2,066.0	
13		118	79.27	1,018.7	
14		135	1,062.67	11,924.8	
15		155	468.44	4,574.3	
16		161	178.39	1,679.0	
17		173	317.53	2,773.3	
18		189	124.94	1,003.7	
19		194	17.90	139.7	
20		201	219.15	1,650.4	
21		213	58.67	417.2	
22		228	34.49	229.5	
23		246	1,968.82	12,149.2	
24		291	313.07	1,632.6	
25		386	222.96	874.9	
26		419	1,982.14	7,171.7	

Assay Class: High Sensitivity DNA Assay
Data Path: C:\...gh Sensitivity DNA Assay_DE04105532_2014-12-15_14-01-58.xad
Created: 12/15/2014 2:01:57 PM
Modified: 12/15/2014 2:43:23 PM

Electropherogram Summary Continued ...

... Peak table for sample 4 : 143

Peak	Size [bp]	Conc. [pg/µl]	Molarity [pmol/l]	Observations
27	795	365.14	695.9	
28	960	1,603.44	2,529.7	
29	1,175	118.02	152.1	
30	1,601	131.84	124.8	
31	1,931	52.13	40.9	
32	3,633	13.36	5.6	
33	3,916	68.88	26.6	
34	5,170	345.01	101.1	
35	6,263	424.24	102.6	
36	7,058	216.35	46.4	
37	8,155	941.98	175.0	
38	10,380	75.00	10.9	Upper Marker

Region table for sample 4 : 143

From [bp]	To [bp]	Corr. Area	% of Total	Average Size [bp]	Size distribution in CV [%]	Conc. [pg/µl]	Molarity [pmol/l]	Color
200	1,000	3,535.9	14	917	6.1	8,640.67	14,279.7	

Assay Class: High Sensitivity DNA Assay
Data Path: C:\...gh Sensitivity DNA Assay_DE04105532_2014-12-15_14-01-58.xad
Created: 12/15/2014 2:01:57 PM
Modified: 12/15/2014 2:43:23 PM

Electropherogram Summary Continued ...

144

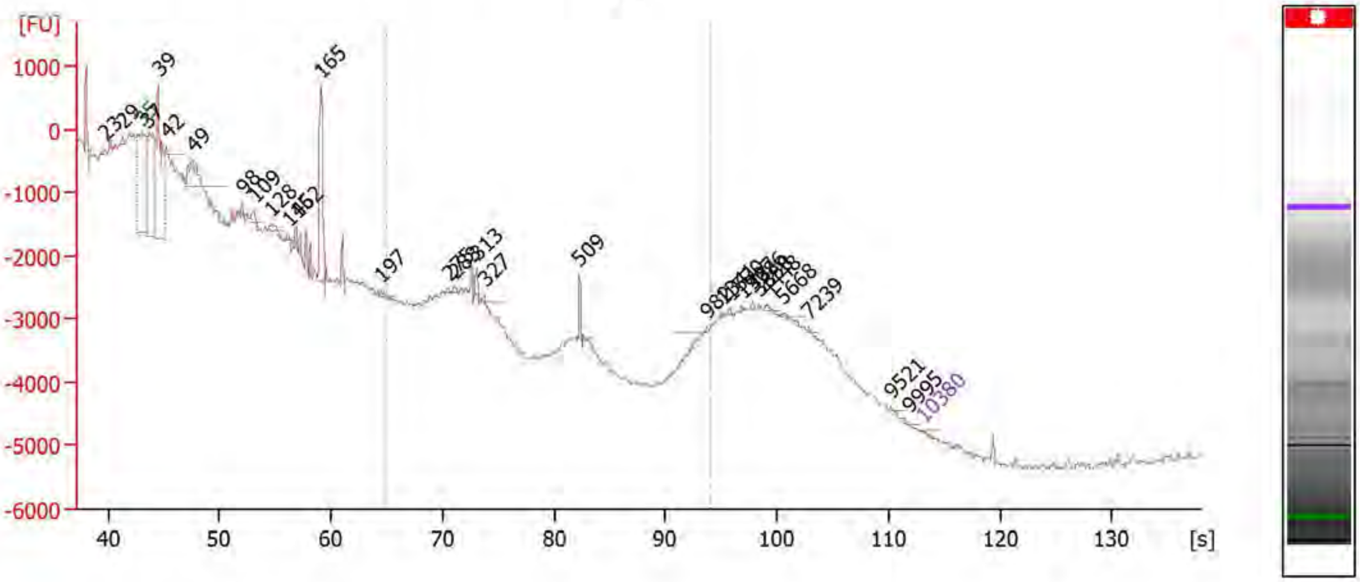

Overall Results for sample 5 : 144

Number of peaks found:	29	Corr. Area 1:	0.0
Noise:	56.3		

Peak table for sample 5 : 144

Peak	Size [bp]	Conc. [pg/µl]	Molarity [pmol/l]	Observations
1	23	0.00	0.0	
2	29	0.00	0.0	
3	35	125.00	5,411.3	Lower Marker
4	37	16,648.61	689,743.8	
5	39	18,554.91	713,006.3	
6	42	318.75	11,617.2	
7	49	5,623.73	175,417.8	
8	98	306.66	4,753.9	
9	109	685.27	9,491.6	
10	128	915.26	10,859.1	
11	146	237.59	2,465.3	
12	152	980.74	9,780.7	
13	165	9,165.67	84,201.1	
14	197	400.65	3,079.0	
15	275	108.77	600.1	
16	283	185.62	993.9	
17	313	550.98	2,666.1	
18	327	172.08	798.1	
19	509	1,087.61	3,237.5	
20	982	421.10	649.4	
21	1,341	57.17	64.6	
22	1,570	92.52	89.3	
23	1,907	33.36	26.5	
24	3,386	70.25	31.4	
25	3,858	27.55	10.8	
26	4,448	124.41	42.4	

Assay Class: High Sensitivity DNA Assay
Data Path: C:\...gh Sensitivity DNA Assay_DE04105532_2014-12-15_14-01-58.xad
Created: 12/15/2014 2:01:57 PM
Modified: 12/15/2014 2:43:23 PM

Electropherogram Summary Continued ...

... Peak table for sample 5 : 144

Peak	Size [bp]	Conc. [pg/µl]	Molarity [pmol/l]	Observations
27	5,668	241.29	64.5	
28	7,239	45.10	9.4	
29	9,521	95.16	15.1	
30	9,995	50.62	7.7	
31	10,380	75.00	10.9	Upper Marker

Region table for sample 5 : 144

From [bp]	To [bp]	Corr. Area	% of Total	Average Size [bp]	Size distribution in CV [%]	Conc. [pg/µl]	Molarity [pmol/l]	Co lor
200	1,000	0.0	0	0	0.0	0.00	0.0	

Assay Class: High Sensitivity DNA Assay
Data Path: C:\...gh Sensitivity DNA Assay_DE04105532_2014-12-15_14-01-58.xad
Created: 12/15/2014 2:01:57 PM
Modified: 12/15/2014 2:43:23 PM

Electropherogram Summary Continued ...

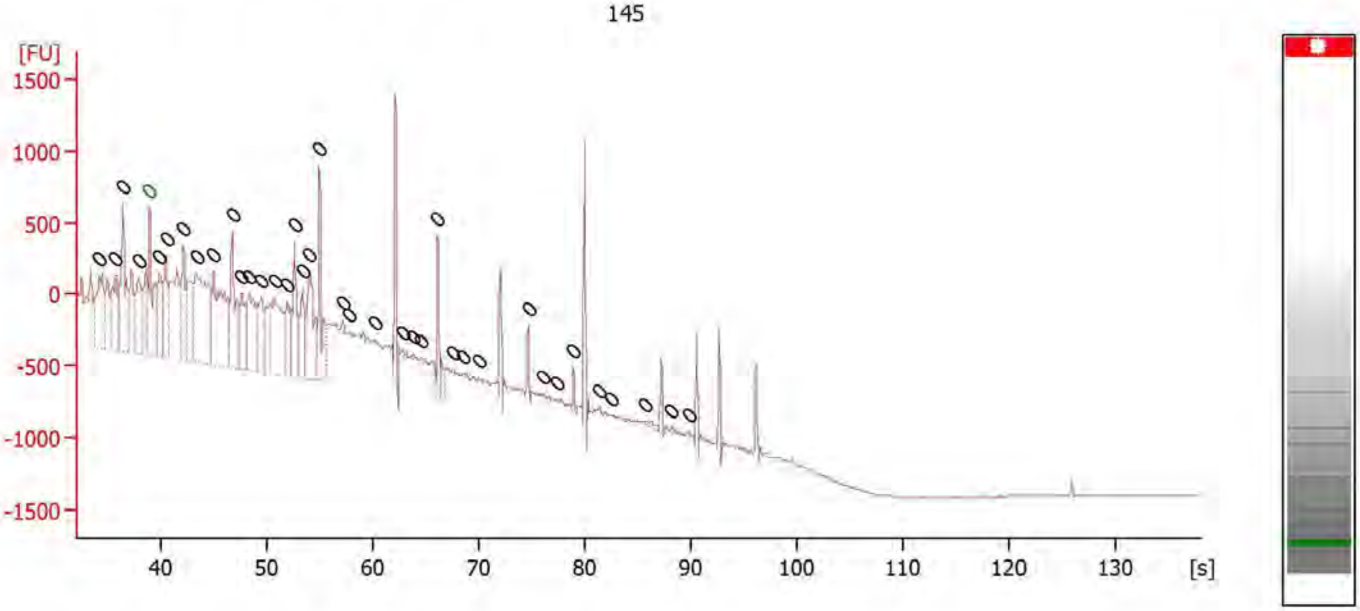

Overall Results for sample 6 : 145

Number of peaks found: 38 Noise: 0.6

Peak table for sample 6 : 145

Peak	Size [bp]	Conc. [pg/µl]	Molarity [pmol/l]	Observations
1	0	0.00	0.0	
2	0	0.00	0.0	
3	0	0.00	0.0	
4	0	0.00	0.0	
5	0	0.00	0.0	Lower Marker
6	0	0.00	0.0	
7	0	0.00	0.0	
8	0	0.00	0.0	
9	0	0.00	0.0	
10	0	0.00	0.0	
11	0	0.00	0.0	
12	0	0.00	0.0	
13	0	0.00	0.0	
14	0	0.00	0.0	
15	0	0.00	0.0	
16	0	0.00	0.0	
17	0	0.00	0.0	
18	0	0.00	0.0	
19	0	0.00	0.0	
20	0	0.00	0.0	
21	0	0.00	0.0	
22	0	0.00	0.0	
23	0	0.00	0.0	
24	0	0.00	0.0	
25	0	0.00	0.0	
26	0	0.00	0.0	
27	0	0.00	0.0	

Assay Class: High Sensitivity DNA Assay
Data Path: C:\...gh Sensitivity DNA Assay_DE04105532_2014-12-15_14-01-58.xad

Created: 12/15/2014 2:01:57 PM
Modified: 12/15/2014 2:43:23 PM

Electropherogram Summary Continued ...

... Peak table for sample 6 : 145

Peak	Size [bp]	Conc. [pg/µl]	Molarity [pmol/l]	Observations
28	0	0.00	0.0	
29	0	0.00	0.0	
30	0	0.00	0.0	
31	0	0.00	0.0	
32	0	0.00	0.0	
33	0	0.00	0.0	
34	0	0.00	0.0	
35	0	0.00	0.0	
36	0	0.00	0.0	
37	0	0.00	0.0	
38	0	0.00	0.0	
39	0	0.00	0.0	

Assay Class: High Sensitivity DNA Assay
Data Path: C:\...gh Sensitivity DNA Assay_DE04105532_2014-12-15_14-01-58.xad
Created: 12/15/2014 2:01:57 PM
Modified: 12/15/2014 2:43:23 PM

Electropherogram Summary Continued ...

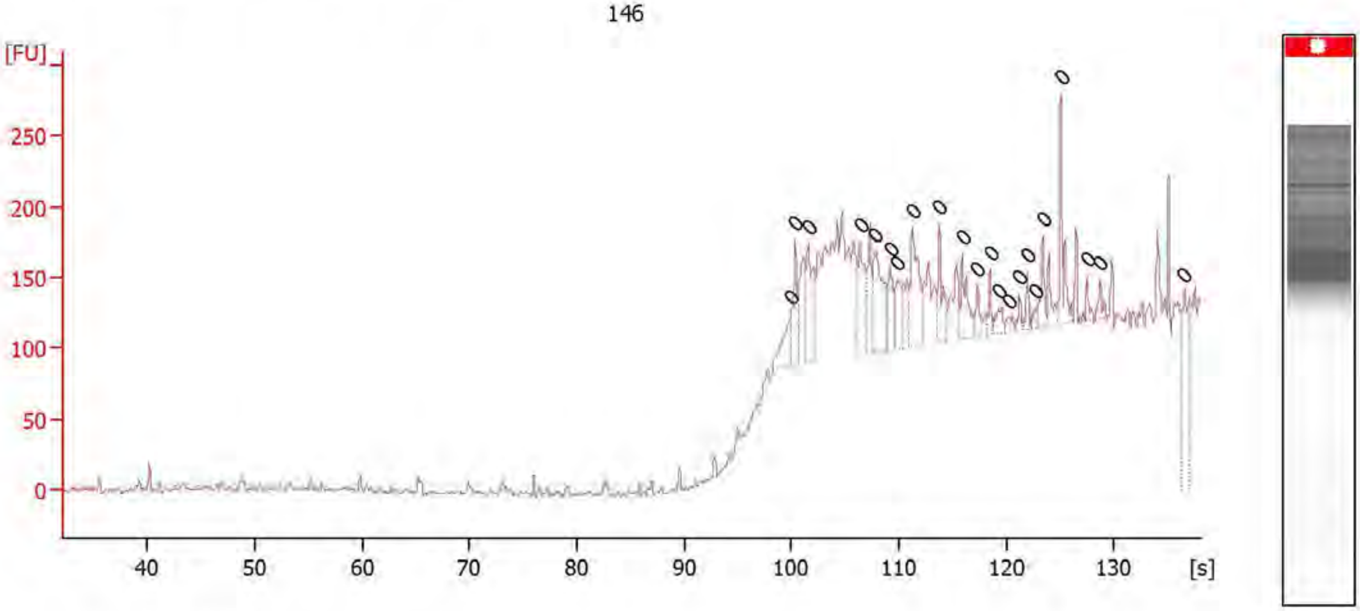

Overall Results for sample 7 : 146

Number of peaks found: 0 Noise: 1.8

Peak table for sample 7 : 146

Peak	Size [bp]	Conc. [pg/µl]	Molarity [pmol/l]	Observations
1	0	0.00	0.0	
2	0	0.00	0.0	
3	0	0.00	0.0	
4	0	0.00	0.0	
5	0	0.00	0.0	
6	0	0.00	0.0	
7	0	0.00	0.0	
8	0	0.00	0.0	
9	0	0.00	0.0	
10	0	0.00	0.0	
11	0	0.00	0.0	
12	0	0.00	0.0	
13	0	0.00	0.0	
14	0	0.00	0.0	
15	0	0.00	0.0	
16	0	0.00	0.0	
17	0	0.00	0.0	
18	0	0.00	0.0	
19	0	0.00	0.0	
20	0	0.00	0.0	
21	0	0.00	0.0	
22	0	0.00	0.0	

Assay Class: High Sensitivity DNA Assay
Data Path: C:\...gh Sensitivity DNA Assay_DE04105532_2014-12-15_14-01-58.xad
Created: 12/15/2014 2:01:57 PM
Modified: 12/15/2014 2:43:23 PM

Electropherogram Summary Continued ...

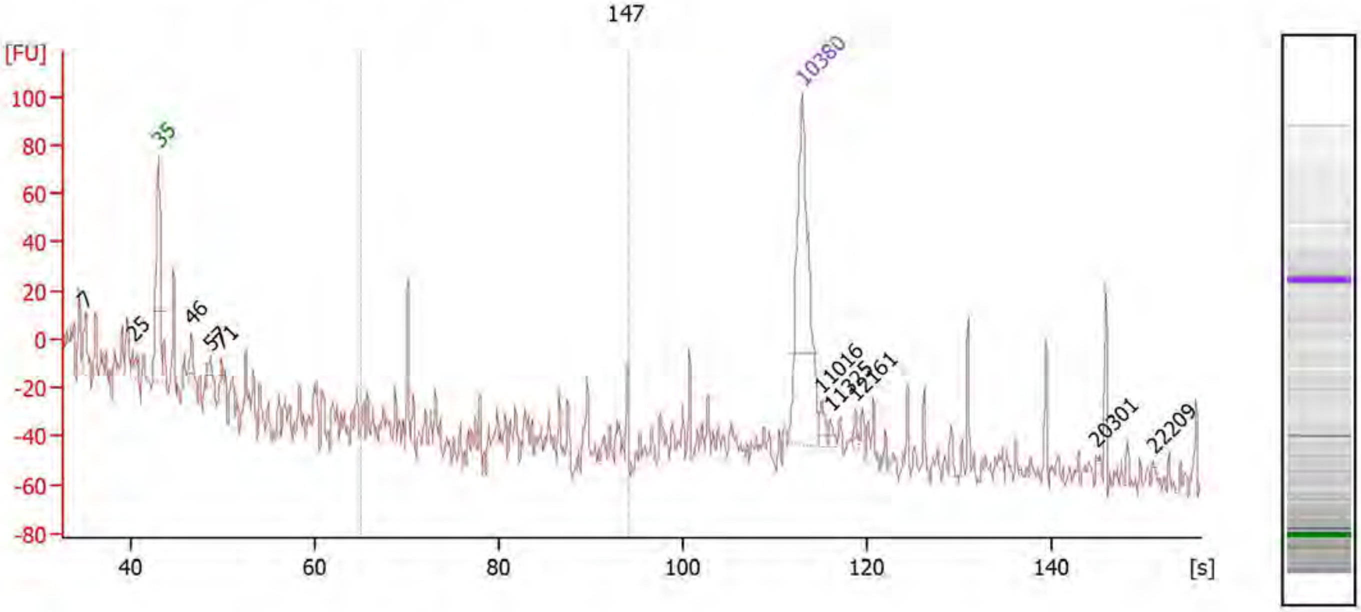

Overall Results for sample 8 : **147**

Number of peaks found:	10	Corr. Area 1:	9.5
Noise:	9.2		

Peak table for sample 8 : **147**

Peak		Size [bp]	Conc. [pg/µl]	Molarity [pmol/l]	Observations
1		7	0.00	0.0	
2		25	0.00	0.0	
3	◀	35	125.00	5,411.3	Lower Marker
4		46	14.17	468.2	
5		57	10.99	291.6	
6		71	6.73	142.8	
7	▶	10,380	75.00	10.9	Upper Marker
8		11,016	0.00	0.0	
9		11,325	0.00	0.0	
10		12,161	0.00	0.0	
11		20,301	0.00	0.0	
12		22,209	0.00	0.0	

Region table for sample 8 : **147**

From [bp]	To [bp]	Corr. Area	% of Total	Average Size [bp]	Size distribution in CV [%]	Conc. [pg/µl]	Molarity [pmol/l]	Co lor
200	1,000	9.5	11	403	66.0	6.12	32.0	■

Assay Class: High Sensitivity DNA Assay
Data Path: C:\...gh Sensitivity DNA Assay_DE04105532_2014-12-15_14-01-58.xad

Created: 12/15/2014 2:01:57 PM
Modified: 12/15/2014 2:43:23 PM

Electropherogram Summary Continued ...

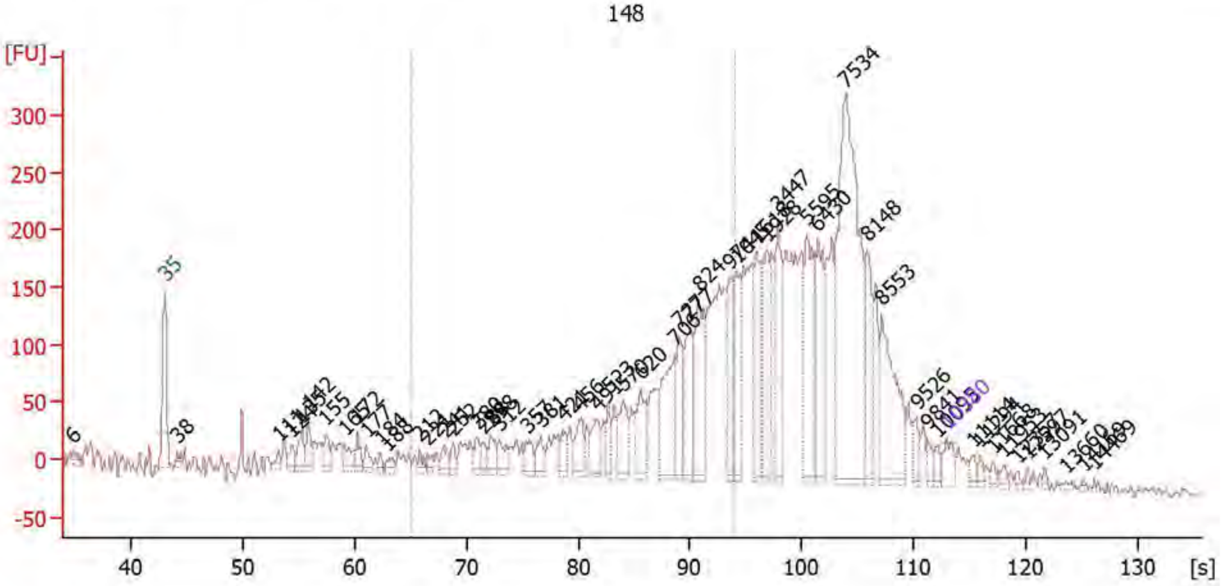

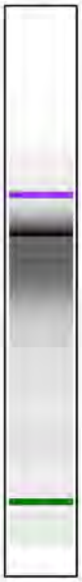

Overall Results for sample 9 : 148

Number of peaks found:	55	Corr. Area 1:	1,890.9
Noise:	6.3		

Peak table for sample 9 : 148

Peak		Size [bp]	Conc. [pg/µl]	Molarity [pmol/l]	Observations
1		6	0.00	0.0	
2	◀	35	125.00	5,411.3	Lower Marker
3		38	59.07	2,326.8	
4		111	66.24	906.8	
5		124	90.03	1,098.7	
6		135	162.16	1,822.5	
7		142	139.10	1,483.2	
8		155	123.20	1,203.4	
9		165	139.06	1,278.3	
10		172	103.97	916.0	
11		177	81.75	701.6	
12		184	38.59	317.8	
13		188	59.01	476.4	
14		212	54.30	388.7	
15		221	49.03	335.5	
16		241	87.99	553.3	
17		252	62.00	372.8	
18		280	82.32	445.5	
19		288	74.81	393.9	
20		298	104.98	533.4	
21		312	121.07	587.8	
22		357	98.96	420.2	
23		381	96.76	384.3	
24		421	83.26	299.4	
25		456	157.87	524.6	
26		491	151.02	466.4	

Assay Class: High Sensitivity DNA Assay
Data Path: C:\...gh Sensitivity DNA Assay_DE04105532_2014-12-15_14-01-58.xad
Created: 12/15/2014 2:01:57 PM
Modified: 12/15/2014 2:43:23 PM

Electropherogram Summary Continued ...

... Peak table for sample 9 : 148

Peak	Size [bp]	Conc. [pg/µl]	Molarity [pmol/l]	Observations
27	523	114.86	333.0	
28	570	188.01	499.6	
29	620	221.65	541.4	
30	706	431.02	924.7	
31	727	229.79	478.8	
32	777	307.66	599.9	
33	824	436.77	803.0	
34	974	294.77	458.7	
35	1,045	273.77	396.9	
36	1,618	271.02	253.8	
37	1,928	261.83	205.8	
38	3,447	335.49	147.5	
39	5,595	428.55	116.1	
40	6,430	379.85	89.5	
41	7,534	1,438.79	289.3	
42	8,148	282.66	52.6	
43	8,553	469.81	83.2	
44	9,526	65.56	10.4	
45	9,841	40.21	6.2	
46	10,095	41.43	6.2	
47	10,380	75.00	10.9	Upper Marker
48	11,114	0.00	0.0	
49	11,294	0.00	0.0	
50	11,668	0.00	0.0	
51	11,953	0.00	0.0	
52	12,357	0.00	0.0	
53	12,597	0.00	0.0	
54	13,091	0.00	0.0	
55	13,660	0.00	0.0	
56	14,169	0.00	0.0	
57	14,469	0.00	0.0	

Region table for sample 9 : 148

From [bp]	To [bp]	Corr. Area	% of Total	Average Size [bp]	Size distribution in CV [%]	Conc. [pg/µl]	Molarity [pmol/l]	Co lor
200	1,000	1,890.9	35	681	31.5	4,825.95	13,308.0	

Assay Class: High Sensitivity DNA Assay
Data Path: C:\...gh Sensitivity DNA Assay_DE04105532_2014-12-15_14-01-58.xad

Created: 12/15/2014 2:01:57 PM
Modified: 12/15/2014 2:43:23 PM

Electropherogram Summary Continued ...

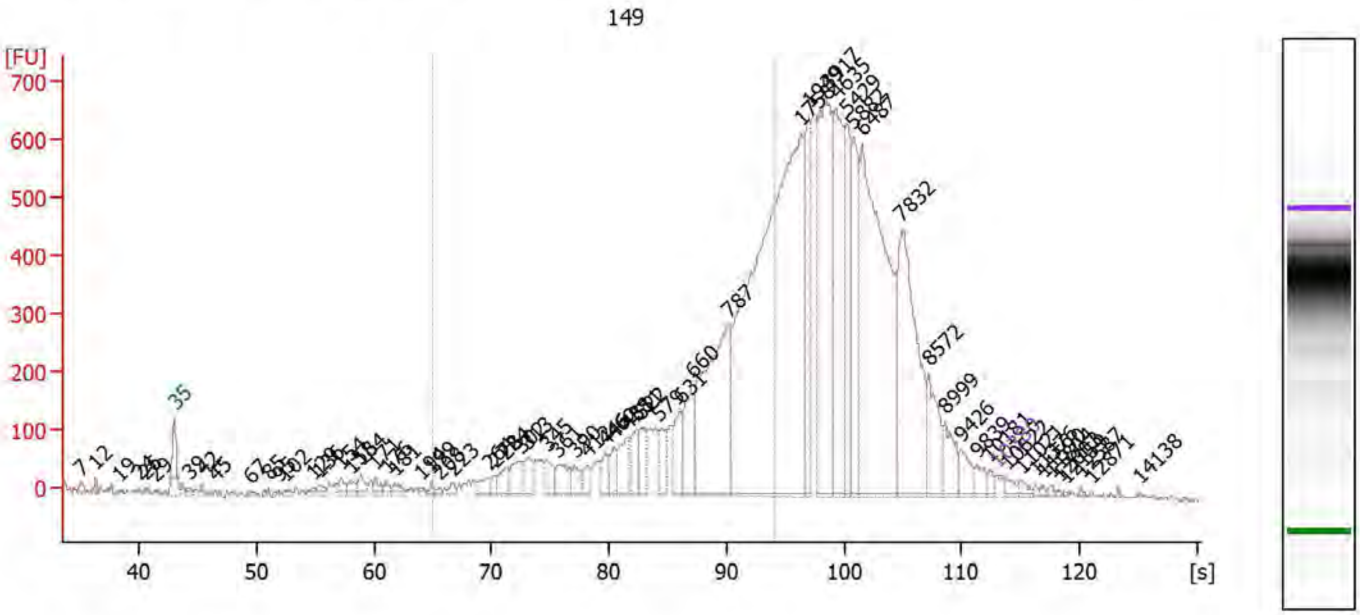

Overall Results for sample 10 : 149

Number of peaks found:	66	Corr. Area 1:	4,428.3
Noise:	5.7		

Peak table for sample 10 : 149

Peak	Size [bp]	Conc. [pg/µl]	Molarity [pmol/l]	Observations
1	7	0.00	0.0	
2	12	0.00	0.0	
3	19	0.00	0.0	
4	24	0.00	0.0	
5	26	0.00	0.0	
6	29	0.00	0.0	
7	35	125.00	5,411.3	Lower Marker
8	39	109.36	4,265.7	
9	42	87.91	3,171.1	
10	45	33.22	1,107.8	
11	67	36.41	817.7	
12	85	47.45	845.3	
13	95	37.96	604.2	
14	102	83.05	1,238.7	
15	129	96.77	1,133.9	
16	136	145.62	1,625.7	
17	154	198.68	1,956.5	
18	158	178.55	1,710.0	
19	164	262.35	2,421.1	
20	171	172.23	1,523.9	
21	176	109.91	944.0	
22	181	187.92	1,575.3	
23	194	74.81	584.9	
24	199	87.55	665.0	
25	208	60.38	439.6	
26	223	106.97	727.0	

Assay Class: High Sensitivity DNA Assay
Data Path: C:\...gh Sensitivity DNA Assay_DE04105532_2014-12-15_14-01-58.xad

Created: 12/15/2014 2:01:57 PM
Modified: 12/15/2014 2:43:23 PM

Electropherogram Summary Continued ...

... Peak table for sample 10 : 149

Peak	Size [bp]	Conc. [pg/µl]	Molarity [pmol/l]	Observations
27	264	229.90	1,317.6	
28	272	147.59	820.9	
29	284	302.74	1,617.8	
30	302	480.00	2,407.7	
31	313	333.54	1,612.8	
32	345	312.18	1,372.7	
33	361	401.39	1,684.4	
34	390	238.66	928.0	
35	412	217.20	797.9	
36	446	261.87	888.8	
37	462	277.57	910.7	
38	488	524.84	1,631.0	
39	511	425.44	1,261.1	
40	522	417.39	1,210.5	
41	579	332.27	869.6	
42	631	531.23	1,275.2	
43	660	859.45	1,973.2	
44	787	3,449.51	6,640.6	
45	1,758	10,031.18	8,645.8	
46	1,949	1,294.77	1,006.4	
47	3,917	2,986.66	1,155.4	
48	4,635	2,205.80	721.1	
49	5,429	1,252.53	349.6	
50	5,882	1,379.28	355.3	
51	6,487	4,993.25	1,166.2	
52	7,832	2,702.24	522.8	
53	8,572	711.59	125.8	
54	8,999	442.91	74.6	
55	9,426	253.39	40.7	
56	9,839	156.05	24.0	
57	10,181	76.98	11.5	
58	10,380	75.00	10.9	Upper Marker
59	10,622	0.00	0.0	
60	11,021	0.00	0.0	
61	11,376	0.00	0.0	
62	11,590	0.00	0.0	
63	11,804	0.00	0.0	
64	12,003	0.00	0.0	
65	12,159	0.00	0.0	
66	12,587	0.00	0.0	
67	12,871	0.00	0.0	
68	14,138	0.00	0.0	

Region table for sample 10 : 149

From [bp]	To [bp]	Corr. Area	% of Total	Average Size [bp]	Size distribution in CV [%]	Conc. [pg/µl]	Molarity [pmol/l]	Color
200	1,000	4,428.3	35	694	31.0	17,799.31	47,937.2	

Assay Class: High Sensitivity DNA Assay
Data Path: C:\...gh Sensitivity DNA Assay_DE04105532_2014-12-15_14-01-58.xad

Created: 12/15/2014 2:01:57 PM
Modified: 12/15/2014 2:43:23 PM

Electropherogram Summary Continued ...

151

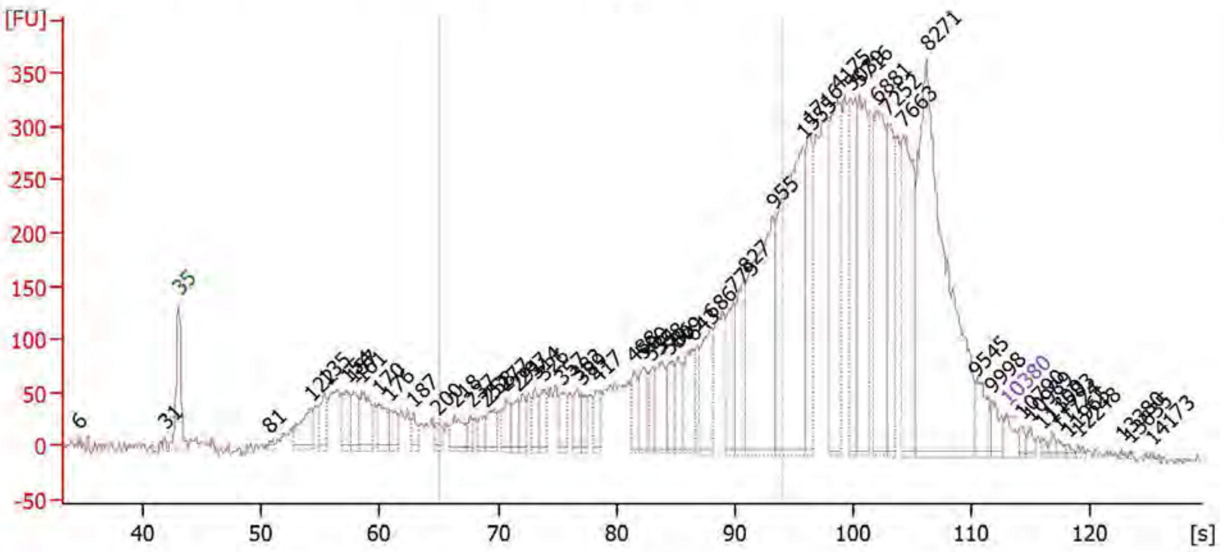

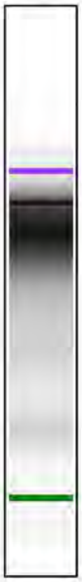

Overall Results for sample 11 : 151

Number of peaks found: 57

Corr. Area 1: 2,914.7

Noise: 4.7

Peak table for sample 11 : 151

Peak	Size [bp]	Conc. [pg/µl]	Molarity [pmol/l]	Observations
1	6	0.00	0.0	
2	31	0.00	0.0	
3	35	125.00	5,411.3	Lower Marker
4	81	46.41	864.7	
5	122	399.53	4,955.7	
6	135	304.37	3,405.9	
7	154	326.51	3,214.4	
8	157	277.83	2,678.7	
9	161	412.54	3,871.2	
10	170	308.11	2,748.8	
11	176	211.86	1,829.0	
12	187	115.12	930.8	
13	200	93.89	712.9	
14	218	238.18	1,655.4	
15	237	111.66	713.7	
16	252	114.85	689.4	
17	258	213.27	1,252.7	
18	277	195.57	1,069.7	
19	291	158.51	826.7	
20	297	155.83	795.8	
21	314	161.83	780.1	
22	326	211.01	979.5	
23	357	181.76	770.5	
24	382	142.15	564.4	
25	389	145.47	566.1	
26	417	168.92	613.7	

Assay Class: High Sensitivity DNA Assay
Data Path: C:\...gh Sensitivity DNA Assay_DE04105532_2014-12-15_14-01-58.xad
Created: 12/15/2014 2:01:57 PM
Modified: 12/15/2014 2:43:23 PM

Electropherogram Summary Continued ...

... Peak table for sample 11 : 151

Peak		Size [bp]	Conc. [pg/µl]	Molarity [pmol/l]	Observations
27		488	194.61	604.7	
28		509	229.50	682.6	
29		534	189.04	536.9	
30		558	261.60	710.9	
31		586	228.83	591.2	
32		609	176.56	439.0	
33		643	319.80	753.6	
34		686	435.41	961.0	
35		775	367.40	718.7	
36		827	426.02	780.9	
37		955	1,436.68	2,278.6	
38		1,555	1,653.46	1,610.9	
39		1,716	547.44	483.3	
40		4,175	791.85	287.4	
41		5,039	620.94	186.7	
42		5,716	718.98	190.6	
43		6,881	830.83	182.9	
44		7,252	427.31	89.3	
45		7,663	808.89	159.9	
46		8,271	2,292.12	419.9	
47		9,545	183.08	29.1	
48		9,998	90.53	13.7	
49	▶	10,380	75.00	10.9	Upper Marker
50		10,790	0.00	0.0	
51		10,989	0.00	0.0	
52		11,300	0.00	0.0	
53		11,583	0.00	0.0	
54		11,724	0.00	0.0	
55		11,965	0.00	0.0	
56		12,248	0.00	0.0	
57		13,380	0.00	0.0	
58		13,635	0.00	0.0	
59		14,173	0.00	0.0	

Region table for sample 11 : 151

From [bp]	To [bp]	Corr. Area	% of Total	Average Size [bp]	Size distribution in CV [%]	Conc. [pg/µl]	Molarity [pmol/l]	Co lor
200	1,000	2,914.7	34	630	37.0	8,694.33	27,288.7	■

Assay Class: High Sensitivity DNA Assay
Data Path: C:\...gh Sensitivity DNA Assay_DE04105532_2014-12-15_14-01-58.xad

Created: 12/15/2014 2:01:57 PM
Modified: 12/15/2014 2:43:23 PM

Gel Image

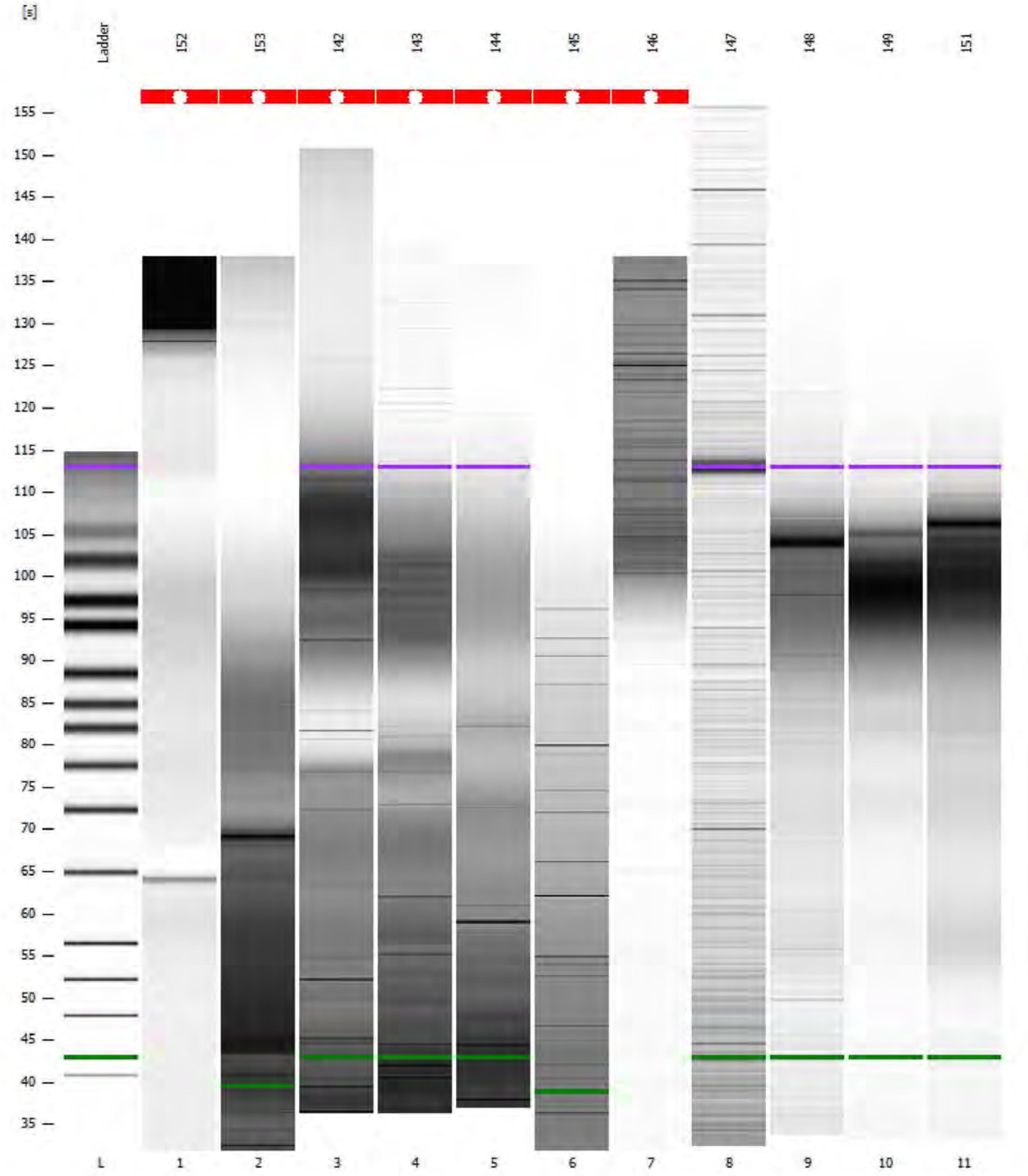

Assay Class: High Sensitivity DNA Assay
Data Path: C:\...gh Sensitivity DNA Assay_DE04105532_2014-12-15_14-01-58.xad
Created: 12/15/2014 2:01:57 PM
Modified: 12/15/2014 2:43:23 PM

Curves

Standard Curve

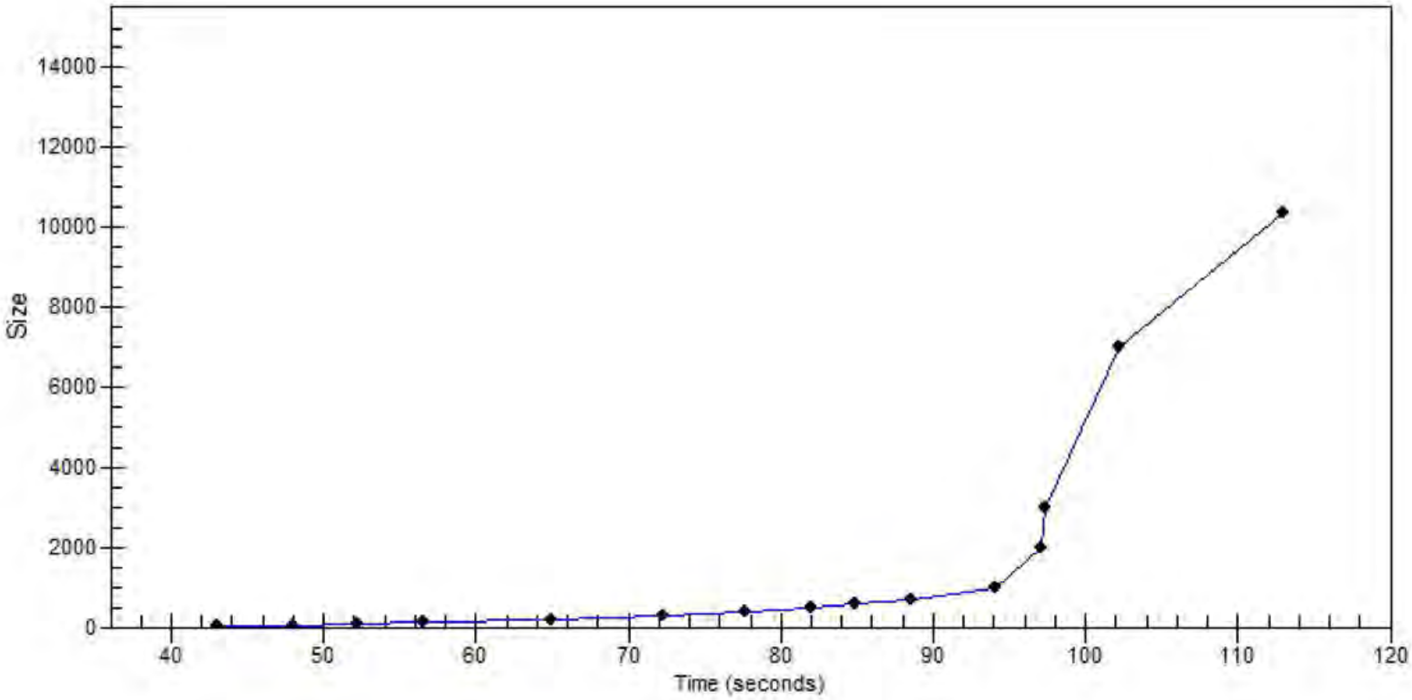

Assay Class: High Sensitivity DNA Assay
Data Path: C:\...gh Sensitivity DNA Assay_DE04105532_2014-12-15_14-01-58.xad

Created: 12/15/2014 2:01:57 PM
Modified: 12/15/2014 2:43:23 PM

Run Logbook

Description	Number	Source	Category	Sub Category	Time	Time Zone	User	Host
Run ended on port 2 (Number of wells acquired: 12)		Instrument	Run		12/15/2014 2:43:19 PM	(GMT +01:00) W. Europe Standard Time	Admin	Datasystem01
Instrument error occurred on port 2, Optical signal too high (1605h)	559	Instrument	Run	Sample 6	12/15/2014 2:27:41 PM	(GMT +01:00) W. Europe Standard Time	Admin	Datasystem01
Instrument error occurred on port 2, Optical signal too high (1605h)	559	Instrument	Run	Sample 1	12/15/2014 2:14:57 PM	(GMT +01:00) W. Europe Standard Time	Admin	Datasystem01
Run started on port 2 (File: C:\Program Files\Agilent\2100 bioanalyzer\2100 expert\Data\2014-12-15\2100 expert_High Sensitivity DNA Assay_DE04105532_2014-12-15_14-01-58.xad)		Instrument	Run		12/15/2014 2:02:03 PM	(GMT +01:00) W. Europe Standard Time	Admin	Datasystem01
Product Number : G2938C		Instrument	Run		12/15/2014 2:02:03 PM	(GMT +01:00) W. Europe Standard Time	Admin	Datasystem01
Name :		Instrument	Run		12/15/2014 2:02:03 PM	(GMT +01:00) W. Europe Standard Time	Admin	Datasystem01
Vendor : Agilent Technologies		Instrument	Run		12/15/2014 2:02:03 PM	(GMT +01:00) W. Europe Standard Time	Admin	Datasystem01
Serial# : DE04105532		Instrument	Run		12/15/2014 2:02:03 PM	(GMT +01:00) W. Europe Standard Time	Admin	Datasystem01
Firmware : C.01.069		Instrument	Run		12/15/2014 2:02:03 PM	(GMT +01:00) W. Europe Standard Time	Admin	Datasystem01
Cartridge : Electrode		Instrument	Run		12/15/2014 2:02:03 PM	(GMT +01:00) W. Europe Standard Time	Admin	Datasystem01

Assay Class: High Sensitivity DNA Assay
Data Path: C:\...gh Sensitivity DNA Assay_DE04105532_2014-12-17_11-51-28.xad

Created: 12/17/2014 11:52:30 AM
Modified: 12/17/2014 12:31:52 PM

Electrophoresis File Run Summary

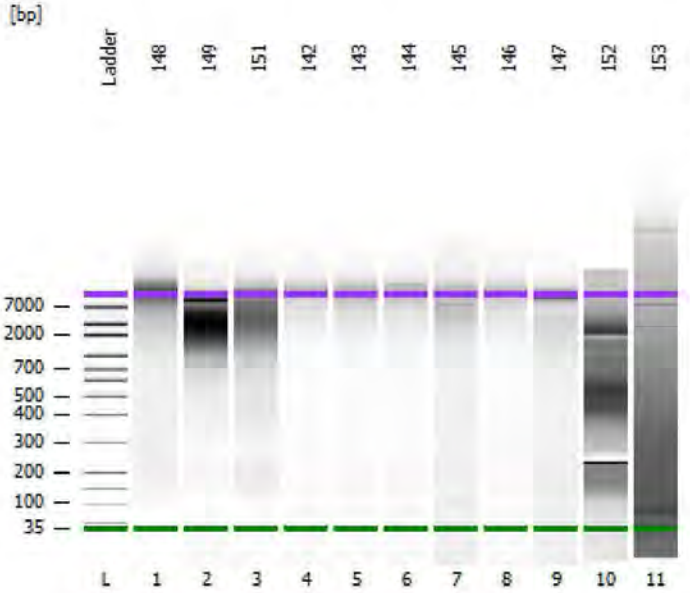

Instrument Information:

Instrument Name: DE04105532
Firmware: C.01.069
Serial#: DE04105532
Type: G2938C

Assay Information:

Assay Origin Path: C:\Program Files\Agilent\2100 bioanalyzer\2100 expert\assays\dsDNA\High Sensitivity DNA.xsy

Assay Class: High Sensitivity DNA Assay

Version: 1.03

148

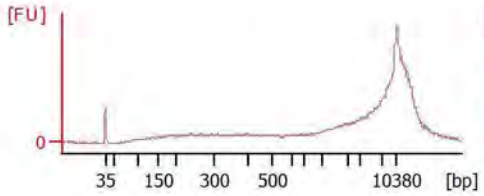

149

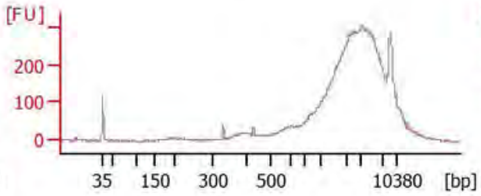

151

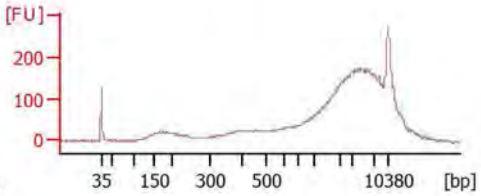

142

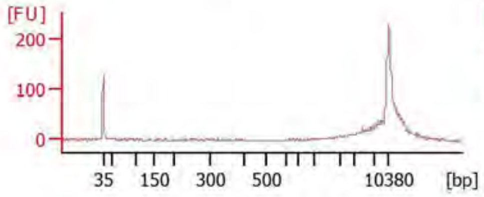

143

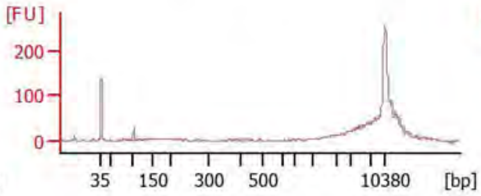

144

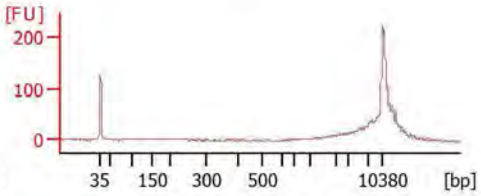

145

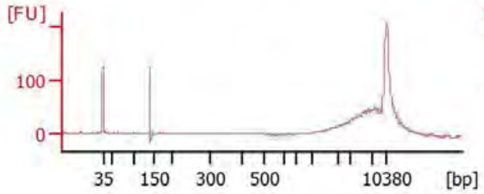

146

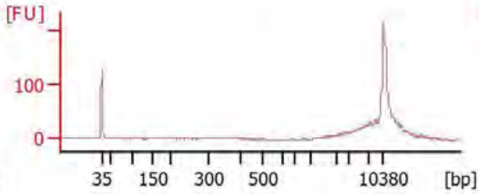

147

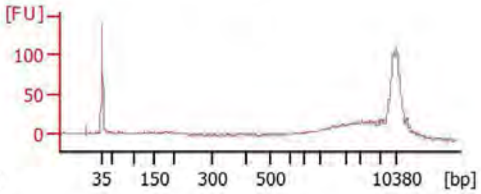

152

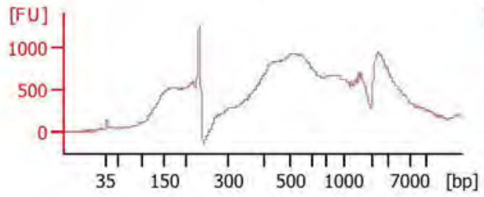

153

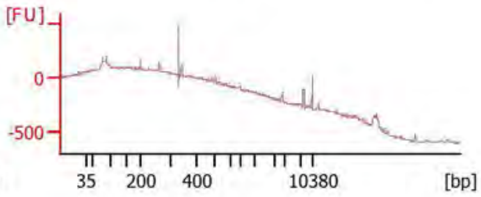

Assay Class: High Sensitivity DNA Assay
Data Path: C:\...gh Sensitivity DNA Assay_DE04105532_2014-12-17_11-51-28.xad

Created: 12/17/2014 11:52:30 AM
Modified: 12/17/2014 12:31:52 PM

Electrophoresis File Run Summary (Chip Summary)

Sample Name	Sample Comment	Rest. Digest	Status	Observation	Result Label	Result Color
148		☐	✔			
149		☐	✔			
151		☐	✔			
142		☐	✔			
143		☐	✔			
144		☐	✔			
145		☐	✔			
146		☐	✔			
147		☐	✔			
152		☐	✔			
153		☐	✔			
Ladder		☐	✔			

Chip Lot # **Reagent Kit Lot #**

Chip Comments :

Assay Class: High Sensitivity DNA Assay
Data Path: C:\...gh Sensitivity DNA Assay_DE04105532_2014-12-17_11-51-28.xad

Created: 12/17/2014 11:52:30 AM
Modified: 12/17/2014 12:31:52 PM

Electrophoresis Assay Details

General Analysis Settings

Number of Available Sample and Ladder Wells (Max.) : 12
Minimum Visible Range [s] : 32
Maximum Visible Range [s] : 138
Start Analysis Time Range [s] : 33
End Analysis Time Range [s] : 137.5
Ladder Concentration [pg/µl] : 1950
Uses Standard Area for Ladder Fragments
Lower Marker Concentration [pg/µl] : 125
Upper Marker Concentration [pg/µl] : 75
Used Upper Marker for Quantitation
Standard Curve Fit is Point to Point
Show Data Aligned to Lower and Upper Marker

Integrator Settings

Integration Start Time [s] : 33.05
Integration End Time [s] : 137
Slope Threshold : 0.8
Height Threshold [FU] : 5
Area Threshold : 0.1
Width Threshold [s] : 0.6
Baseline Plateau [s] : 0.5

Filter Settings

Filter Width [s] : 0.5
Polynomial Order : 4

Ladder

Ladder Peak	Size	Area
1	35	160
2	50	210
3	100	208
4	150	221
5	200	242
6	300	270
7	400	305
8	500	306
9	600	336
10	700	321
11	1000	366
12	2000	413
13	3000	411
14	7000	400
15	10380	214

Assay Class: High Sensitivity DNA Assay
Data Path: C:\...gh Sensitivity DNA Assay_DE04105532_2014-12-17_11-51-28.xad

Created: 12/17/2014 11:52:30 AM
Modified: 12/17/2014 12:31:52 PM

Electropherogram Summary

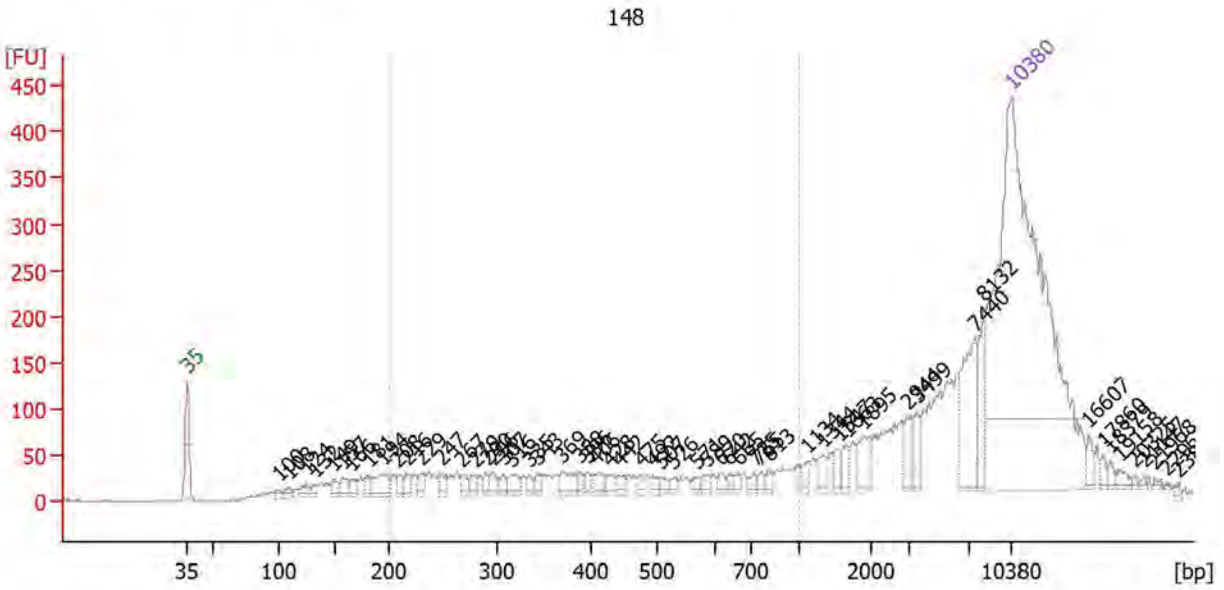

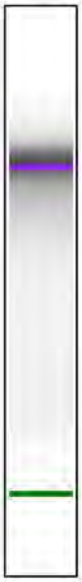

Overall Results for sample 1 : 148

Number of peaks found: 57
Corr. Area 1: 1,011.6
Noise: 0.5

Peak table for sample 1 : 148

Peak	Size [bp]	Conc. [pg/µl]	Molarity [pmol/l]	Observations
1	35	125.00	5,411.3	Lower Marker
2	100	1.10	16.7	
3	108	1.25	17.5	
4	124	2.03	24.8	
5	132	1.31	15.0	
6	149	2.07	21.1	
7	157	2.59	25.0	
8	166	1.93	17.7	
9	181	1.82	15.2	
10	194	4.98	39.0	
11	208	1.95	14.2	
12	216	2.10	14.8	
13	229	1.93	12.7	
14	247	2.20	13.5	
15	267	1.87	10.6	
16	279	1.58	8.6	
17	290	1.44	7.5	
18	296	1.61	8.2	
19	307	1.38	6.8	
20	316	2.47	11.9	
21	335	1.14	5.1	
22	343	1.22	5.4	
23	369	3.16	13.0	
24	388	1.16	4.5	
25	396	1.20	4.6	
26	406	2.13	7.9	

Assay Class: High Sensitivity DNA Assay
Data Path: C:\...gh Sensitivity DNA Assay_DE04105532_2014-12-17_11-51-28.xad
Created: 12/17/2014 11:52:30 AM
Modified: 12/17/2014 12:31:52 PM

Electropherogram Summary Continued ...

... Peak table for sample 1 : 148

Peak	Size [bp]	Conc. [pg/µl]	Molarity [pmol/l]	Observations
27	428	1.44	5.1	
28	442	1.38	4.7	
29	475	0.92	2.9	
30	492	0.95	2.9	
31	507	1.12	3.3	
32	526	0.92	2.7	
33	571	1.04	2.8	
34	589	1.13	2.9	
35	620	1.07	2.6	
36	642	0.82	1.9	
37	665	0.81	1.8	
38	725	1.02	2.1	
39	755	0.92	1.8	
40	813	1.00	1.9	
41	1,134	1.32	1.8	
42	1,344	1.83	2.1	
43	1,547	1.67	1.6	
44	1,663	1.65	1.5	
45	1,895	3.58	2.9	
46	2,944	2.26	1.2	
47	3,499	2.20	1.0	
48	7,440	9.63	2.0	
49	8,132	6.19	1.2	
50	10,380	75.00	10.9	Upper Marker
51	16,607	0.00	0.0	
52	17,860	0.00	0.0	
53	18,379	0.00	0.0	
54	19,158	0.00	0.0	
55	20,325	0.00	0.0	
56	21,147	0.00	0.0	
57	21,968	0.00	0.0	
58	22,963	0.00	0.0	
59	23,871	0.00	0.0	

Region table for sample 1 : 148

From [bp]	To [bp]	Corr. Area	% of Total	Average Size [bp]	Size distribution in CV [%]	Conc. [pg/µl]	Molarity [pmol/l]	Co lor
200	1,000	1,011.6	37	448	45.1	65.87	288.5	

Assay Class: High Sensitivity DNA Assay
Data Path: C:\...gh Sensitivity DNA Assay_DE04105532_2014-12-17_11-51-28.xad

Created: 12/17/2014 11:52:30 AM
Modified: 12/17/2014 12:31:52 PM

Electropherogram Summary Continued ...

149

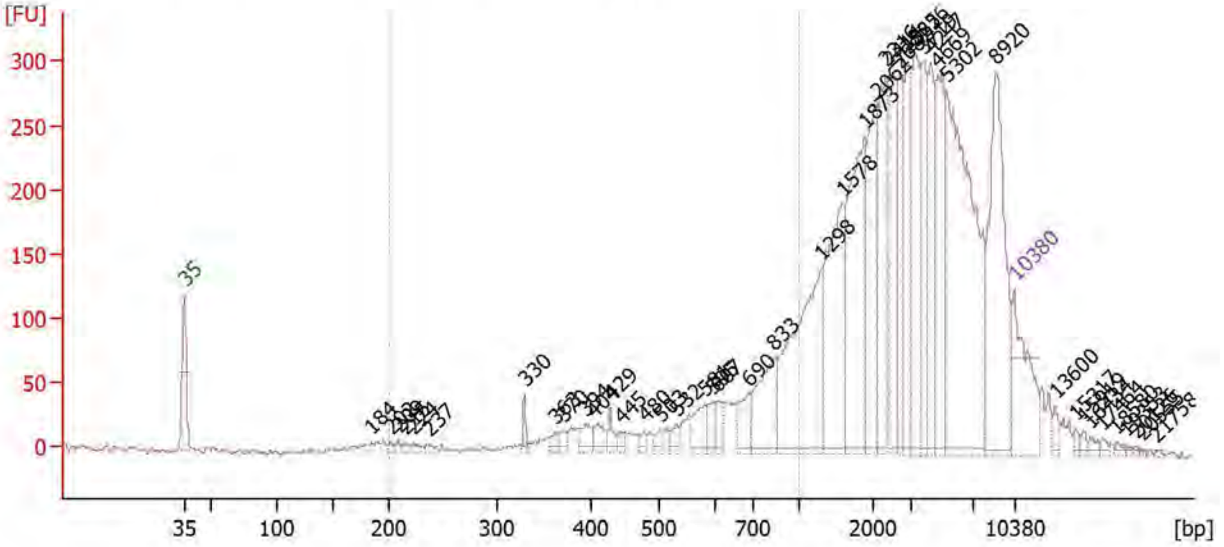

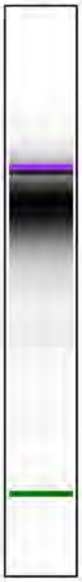

Overall Results for sample 2 : **149**

Number of peaks found:	45	Corr. Area 1:	915.4
Noise:	2.0		

Peak table for sample 2 : **149**

Peak	Size [bp]	Conc. [pg/µl]	Molarity [pmol/l]	Observations
1	35	125.00	5,411.3	Lower Marker
2	184	6.79	55.8	
3	202	4.59	34.3	
4	209	4.64	33.6	
5	215	5.28	37.2	
6	224	4.98	33.7	
7	237	4.54	29.0	
8	330	10.09	46.3	
9	363	9.49	39.6	
10	370	9.72	39.8	
11	394	17.46	67.1	
12	404	18.82	70.6	
13	429	13.41	47.4	
14	445	9.84	33.5	
15	480	8.45	26.7	
16	503	9.38	28.2	
17	532	12.26	34.9	
18	581	29.52	77.0	
19	595	16.11	41.0	
20	607	15.32	38.2	
21	690	32.85	72.2	
22	833	83.81	152.4	
23	1,298	209.45	244.6	
24	1,578	160.10	153.7	
25	1,873	186.37	150.7	
26	2,062	111.52	82.0	

Assay Class: High Sensitivity DNA Assay
Data Path: C:\...gh Sensitivity DNA Assay_DE04105532_2014-12-17_11-51-28.xad

Created: 12/17/2014 11:52:30 AM
Modified: 12/17/2014 12:31:52 PM

Electropherogram Summary Continued ...

... Peak table for sample 2 : 149

Peak	Size [bp]	Conc. [pg/µl]	Molarity [pmol/l]	Observations
27	2,316	103.80	67.9	
28	2,414	89.27	56.0	
29	2,682	76.73	43.3	
30	2,865	96.36	51.0	
31	3,226	103.74	48.7	
32	3,719	78.10	31.8	
33	4,247	76.62	27.3	
34	4,669	114.76	37.2	
35	5,302	330.60	94.5	
36	8,920	191.53	32.5	
37	10,380	75.00	10.9	Upper Marker
38	13,600	0.00	0.0	
39	15,317	0.00	0.0	
40	15,919	0.00	0.0	
41	16,434	0.00	0.0	
42	17,464	0.00	0.0	
43	18,580	0.00	0.0	
44	19,353	0.00	0.0	
45	20,126	0.00	0.0	
46	20,555	0.00	0.0	
47	21,758	0.00	0.0	

Region table for sample 2 : 149

From [bp]	To [bp]	Corr. Area	% of Total	Average Size [bp]	Size distribution in CV [%]	Conc. [pg/µl]	Molarity [pmol/l]	Co lor
200	1,000	915.4	18	650	31.9	523.04	1,481.5	

Assay Class: High Sensitivity DNA Assay
Data Path: C:\...gh Sensitivity DNA Assay_DE04105532_2014-12-17_11-51-28.xad

Created: 12/17/2014 11:52:30 AM
Modified: 12/17/2014 12:31:52 PM

Electropherogram Summary Continued ...

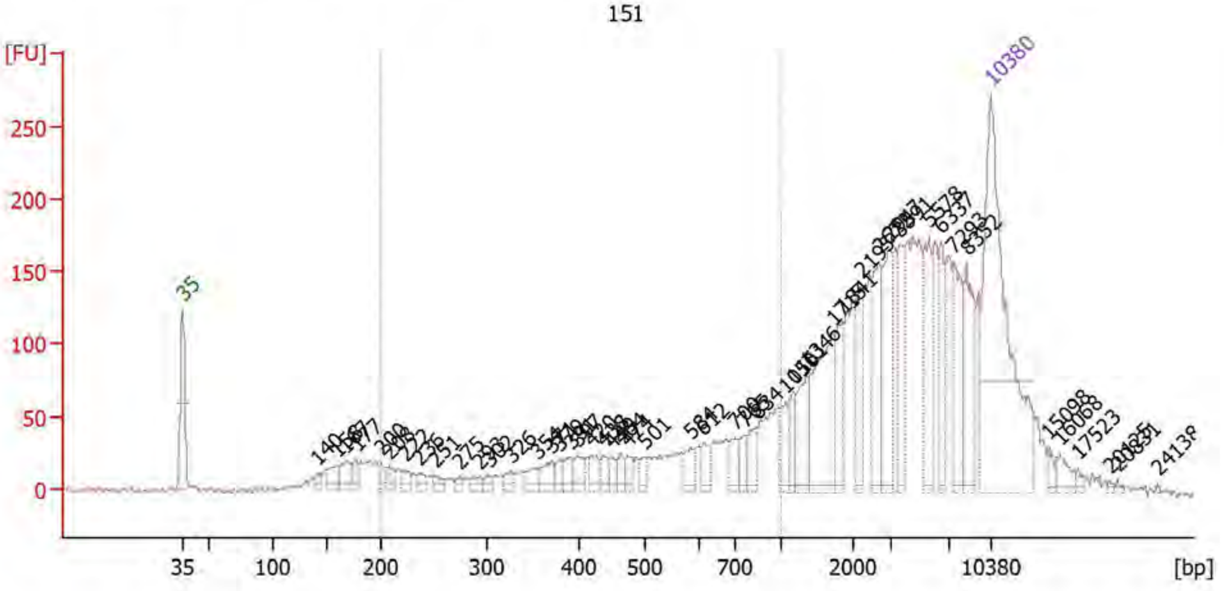

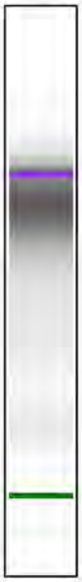

Overall Results for sample 3 : 151

Number of peaks found:	48	Corr. Area 1:	1,016.6
Noise:	1.5		

Peak table for sample 3 : 151

Peak		Size [bp]	Conc. [pg/µl]	Molarity [pmol/l]	Observations
1	◄	35	125.00	5,411.3	Lower Marker
2		140	3.14	33.8	
3		158	7.24	69.4	
4		167	8.87	80.4	
5		177	5.08	43.6	
6		200	4.20	31.8	
7		208	3.93	28.7	
8		222	3.79	25.9	
9		236	3.31	21.2	
10		251	3.29	19.9	
11		275	1.91	10.5	
12		293	3.31	17.1	
13		302	2.24	11.3	
14		326	2.89	13.5	
15		354	5.03	21.5	
16		371	6.38	26.1	
17		379	3.45	13.8	
18		391	4.99	19.3	
19		397	5.67	21.6	
20		420	5.29	19.1	
21		438	3.97	13.7	
22		449	3.27	11.0	
23		464	3.66	12.0	
24		474	4.29	13.7	
25		501	3.70	11.2	
26		584	5.41	14.1	

Assay Class: High Sensitivity DNA Assay
Data Path: C:\...gh Sensitivity DNA Assay_DE04105532_2014-12-17_11-51-28.xad

Created: 12/17/2014 11:52:30 AM
Modified: 12/17/2014 12:31:52 PM

Electropherogram Summary Continued ...

... Peak table for sample 3 : 151

Peak		Size [bp]	Conc. [pg/µl]	Molarity [pmol/l]	Observations
27		612	5.31	13.1	
28		700	6.05	13.1	
29		765	5.10	10.1	
30		834	6.51	11.8	
31		1,050	7.38	10.6	
32		1,161	6.98	9.1	
33		1,346	13.57	15.3	
34		1,715	30.62	27.0	
35		1,841	12.91	10.6	
36		2,195	12.37	8.5	
37		2,672	18.58	10.5	
38		2,947	19.39	10.0	
39		3,591	14.86	6.3	
40		5,578	18.84	5.1	
41		6,337	14.30	3.4	
42		7,293	18.48	3.8	
43		8,352	20.07	3.6	
44	▶	10,380	75.00	10.9	Upper Marker
45		15,098	0.00	0.0	
46		16,068	0.00	0.0	
47		17,523	0.00	0.0	
48		20,125	0.00	0.0	
49		20,831	0.00	0.0	
50		24,138	0.00	0.0	

Region table for sample 3 : 151

From [bp]	To [bp]	Corr. Area	% of Total	Average Size [bp]	Size distribution in CV [%]	Conc. [pg/µl]	Molarity [pmol/l]	Co lor
200	1,000	1,016.6	28	547	39.7	189.31	686.4	■

Assay Class: High Sensitivity DNA Assay
Data Path: C:\...gh Sensitivity DNA Assay_DE04105532_2014-12-17_11-51-28.xad
Created: 12/17/2014 11:52:30 AM
Modified: 12/17/2014 12:31:52 PM

Electropherogram Summary Continued ...

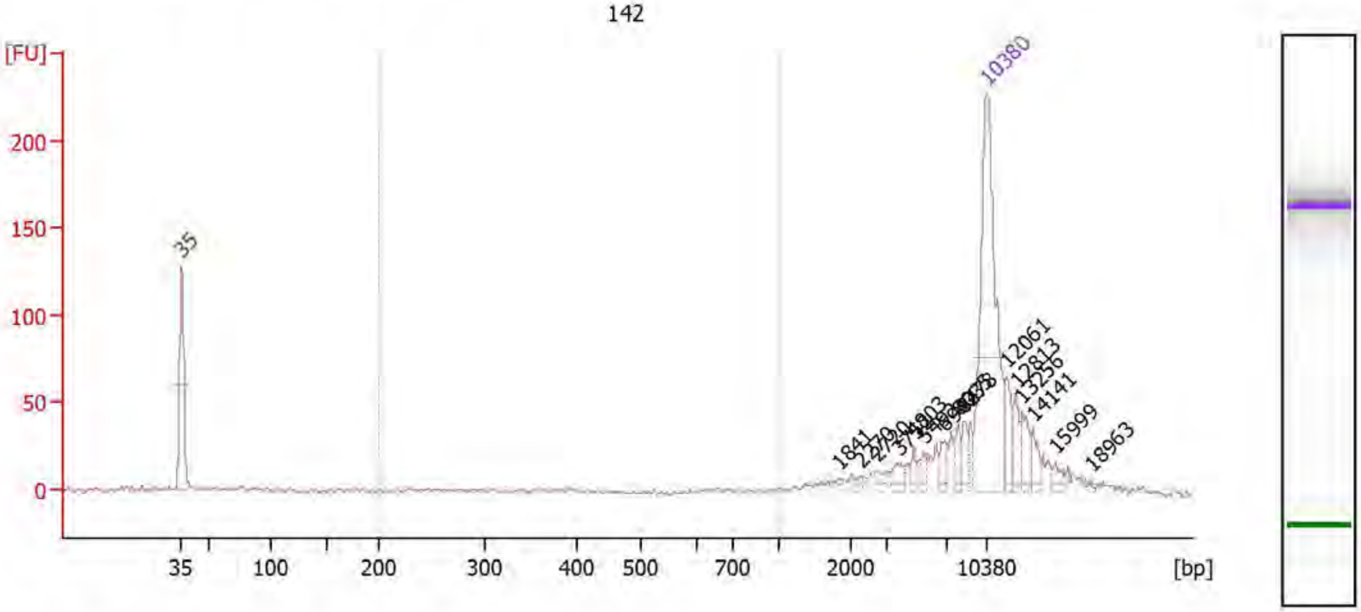

Overall Results for sample 4 : 142

Number of peaks found:	15	Corr. Area 1:	0.7
Noise:	1.0		

Peak table for sample 4 : 142

Peak		Size [bp]	Conc. [pg/µl]	Molarity [pmol/l]	Observations
1	◀	35	125.00	5,411.3	Lower Marker
2		1,841	1.12	0.9	
3		2,270	1.48	1.0	
4		2,720	2.18	1.2	
5		3,752	4.48	1.8	
6		4,803	3.03	1.0	
7		5,492	2.93	0.8	
8		6,942	4.89	1.1	
9		8,035	4.74	0.9	
10		8,478	5.05	0.9	
11	▶	10,380	75.00	10.9	Upper Marker
12		12,061	0.00	0.0	
13		12,813	0.00	0.0	
14		13,256	0.00	0.0	
15		14,141	0.00	0.0	
16		15,999	0.00	0.0	
17		18,963	0.00	0.0	

Region table for sample 4 : 142

From [bp]	To [bp]	Corr. Area	% of Total	Average Size [bp]	Size distribution in CV [%]	Conc. [pg/µl]	Molarity [pmol/l]	Color
200	1,000	0.7	0	359	41.8	0.25	1.3	

Assay Class: High Sensitivity DNA Assay
Data Path: C:\...gh Sensitivity DNA Assay_DE04105532_2014-12-17_11-51-28.xad

Created: 12/17/2014 11:52:30 AM
Modified: 12/17/2014 12:31:52 PM

Electropherogram Summary Continued ...

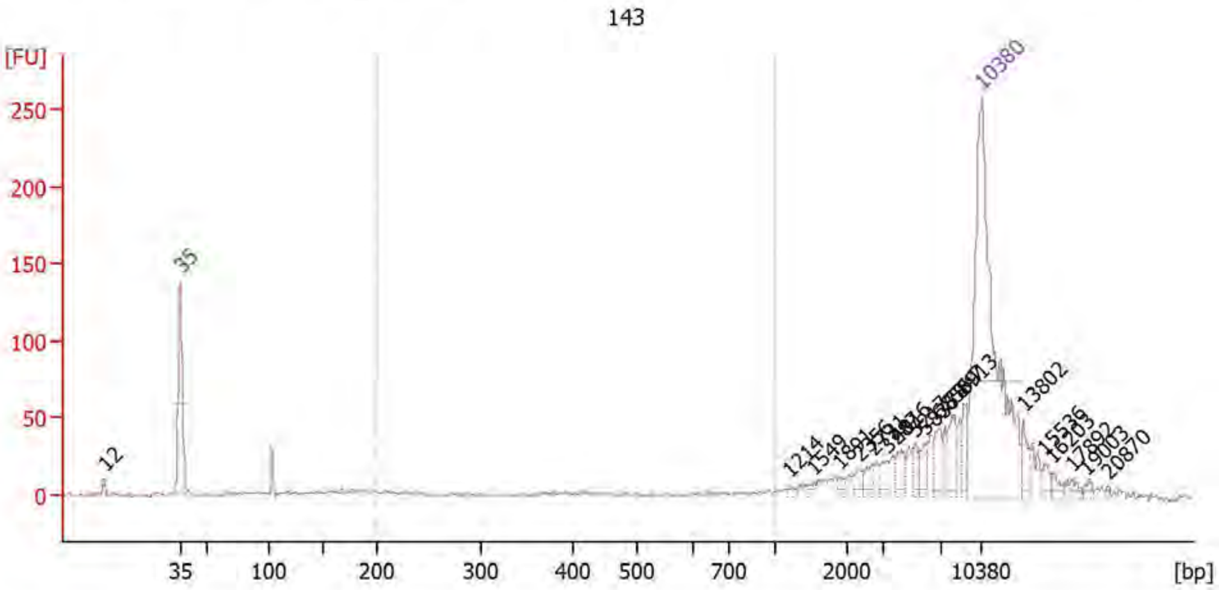

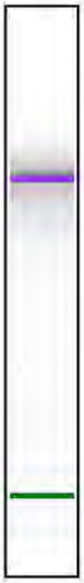

Overall Results for sample 5 : 143

Number of peaks found:	19	Corr. Area 1:	14.9
Noise:	0.8		

Peak table for sample 5 : 143

Peak		Size [bp]	Conc. [pg/µl]	Molarity [pmol/l]	Observations
1		12	0.00	0.0	
2	◀	35	125.00	5,411.3	Lower Marker
3		1,214	0.97	1.2	
4		1,549	1.02	1.0	
5		1,891	1.42	1.1	
6		2,356	2.71	1.7	
7		2,721	2.48	1.4	
8		3,287	2.90	1.3	
9		4,016	4.54	1.7	
10		5,217	3.13	0.9	
11		5,836	3.52	0.9	
12		6,856	5.34	1.2	
13		7,847	6.49	1.3	
14		8,913	5.25	0.9	
15	▶	10,380	75.00	10.9	Upper Marker
16		13,802	0.00	0.0	
17		15,536	0.00	0.0	
18		16,203	0.00	0.0	
19		17,892	0.00	0.0	
20		19,003	0.00	0.0	
21		20,870	0.00	0.0	

Region table for sample 5 : 143

From [bp]	To [bp]	Corr. Area	% of Total	Average Size [bp]	Size distribution in CV [%]	Conc. [pg/µl]	Molarity [pmol/l]	Color
200	1,000	14.9	3	428	60.3	3.70	19.7	■

Assay Class: High Sensitivity DNA Assay
Data Path: C:\...gh Sensitivity DNA Assay_DE04105532_2014-12-17_11-51-28.xad

Created: 12/17/2014 11:52:30 AM
Modified: 12/17/2014 12:31:52 PM

Electropherogram Summary Continued ...

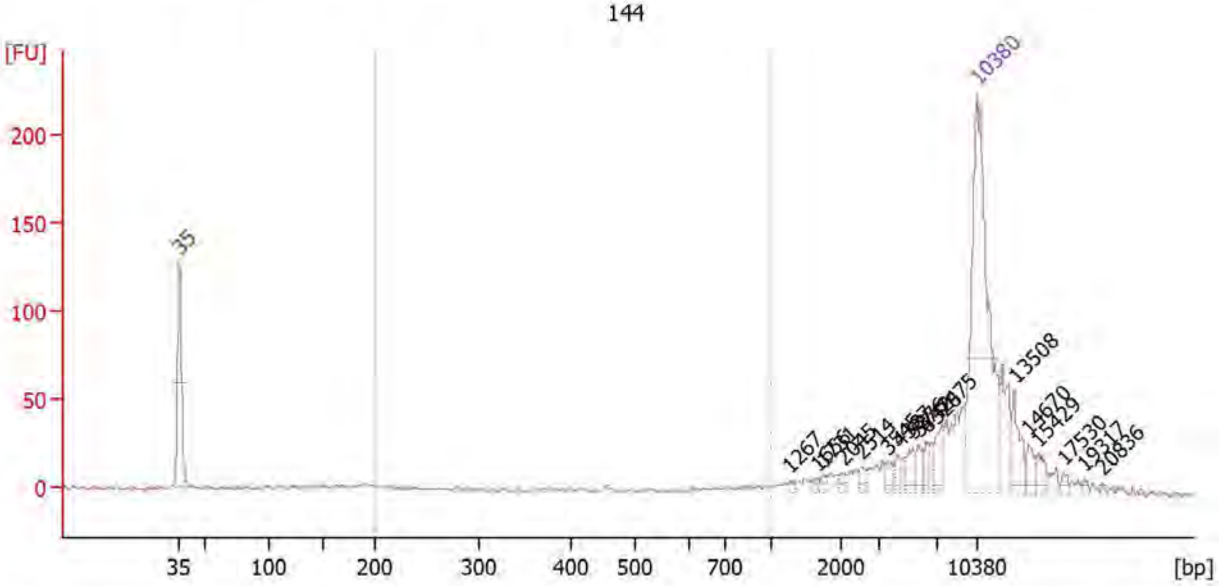

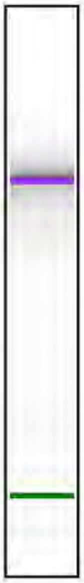

Overall Results for sample 6 : 144

Number of peaks found:	17	Corr. Area 1:	41.4
Noise:	0.9		

Peak table for sample 6 : 144

Peak		Size [bp]	Conc. [pg/µl]	Molarity [pmol/l]	Observations
1	◄	35	125.00	5,411.3	Lower Marker
2		1,267	1.02	1.2	
3		1,656	1.41	1.3	
4		1,731	1.54	1.4	
5		2,045	1.59	1.2	
6		2,514	2.32	1.4	
7		3,545	2.74	1.2	
8		4,387	2.84	1.0	
9		5,376	4.58	1.3	
10		5,742	3.41	0.9	
11		6,328	3.67	0.9	
12		7,475	6.58	1.3	
13	►	10,380	75.00	10.9	Upper Marker
14		13,508	0.00	0.0	
15		14,670	0.00	0.0	
16		15,429	0.00	0.0	
17		17,530	0.00	0.0	
18		19,317	0.00	0.0	
19		20,836	0.00	0.0	

Region table for sample 6 : 144

From [bp]	To [bp]	Corr. Area	% of Total	Average Size [bp]	Size distribution in CV [%]	Conc. [pg/µl]	Molarity [pmol/l]	Co lor
200	1,000	41.4	8	603	39.1	12.67	44.3	■

Assay Class: High Sensitivity DNA Assay
Data Path: C:\...gh Sensitivity DNA Assay_DE04105532_2014-12-17_11-51-28.xad

Created: 12/17/2014 11:52:30 AM
Modified: 12/17/2014 12:31:52 PM

Electropherogram Summary Continued ...

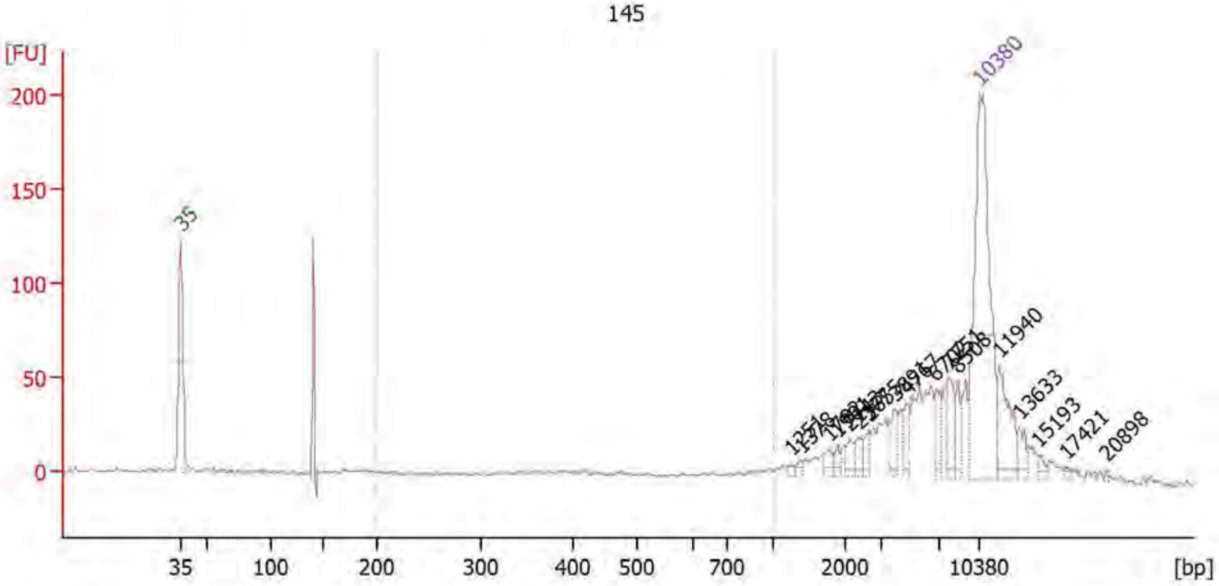

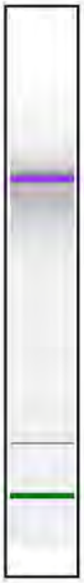

Overall Results for sample 7 : 145

Number of peaks found: 17
Corr. Area 1: 68.5
Noise: 1.1

Peak table for sample 7 : 145

Peak		Size [bp]	Conc. [pg/µl]	Molarity [pmol/l]	Observations
1	◄	35	125.00	5,411.3	Lower Marker
2		1,251	1.23	1.5	
3		1,378	1.59	1.7	
4		1,782	3.40	2.9	
5		1,901	2.65	2.1	
6		2,143	3.96	2.8	
7		2,377	4.12	2.6	
8		2,655	3.71	2.1	
9		3,891	6.98	2.7	
10		4,767	5.96	1.9	
11		6,702	7.78	1.8	
12		7,751	9.51	1.9	
13		8,508	6.80	1.2	
14	►	10,380	75.00	10.9	Upper Marker
15		11,940	0.00	0.0	
16		13,633	0.00	0.0	
17		15,193	0.00	0.0	
18		17,421	0.00	0.0	
19		20,898	0.00	0.0	

Region table for sample 7 : 145

From [bp]	To [bp]	Corr. Area	% of Total	Average Size [bp]	Size distribution in CV [%]	Conc. [pg/µl]	Molarity [pmol/l]	Color
200	1,000	68.5	9	513	43.6	25.19	97.2	■

Assay Class: High Sensitivity DNA Assay
Data Path: C:\...gh Sensitivity DNA Assay_DE04105532_2014-12-17_11-51-28.xad

Created: 12/17/2014 11:52:30 AM
Modified: 12/17/2014 12:31:52 PM

Electropherogram Summary Continued ...

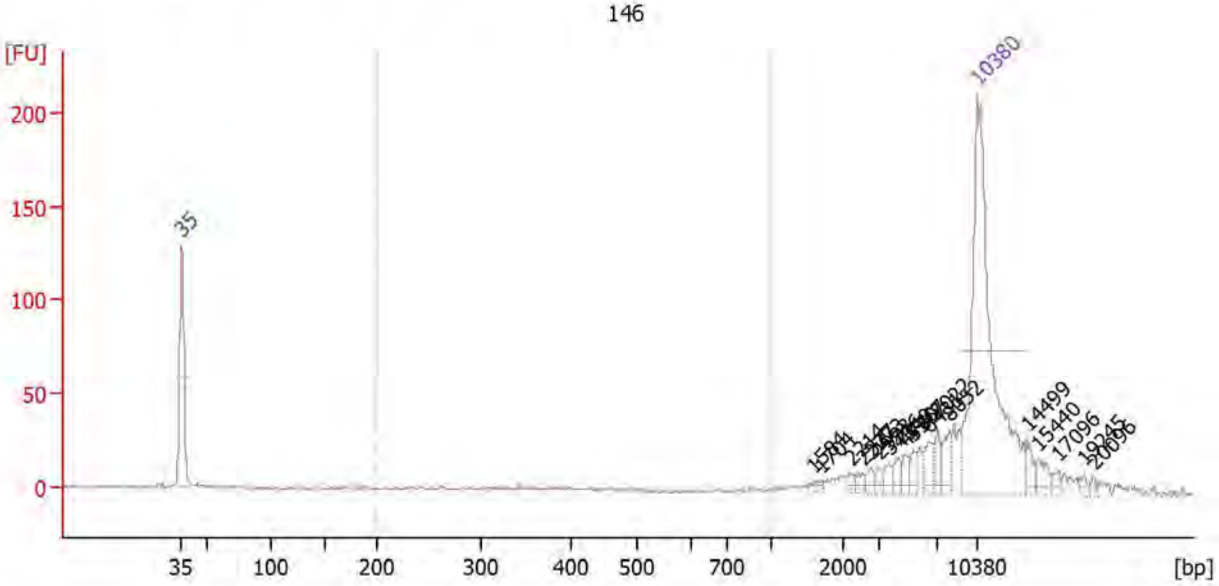

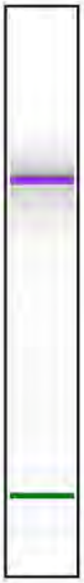

Overall Results for sample 8 : 146

Number of peaks found:	18	Corr. Area 1:	22.7
Noise:	0.8		

Peak table for sample 8 : 146

Peak		Size [bp]	Conc. [pg/µl]	Molarity [pmol/l]	Observations
1	◀	35	125.00	5,411.3	Lower Marker
2		1,584	0.93	0.9	
3		1,704	0.89	0.8	
4		2,214	1.52	1.0	
5		2,537	1.71	1.0	
6		2,773	2.03	1.1	
7		2,993	1.82	0.9	
8		3,716	2.36	1.0	
9		4,340	2.61	0.9	
10		4,780	2.74	0.9	
11		5,367	2.60	0.7	
12		6,431	4.79	1.1	
13		7,022	3.60	0.8	
14		8,052	4.83	0.9	
15	▶	10,380	75.00	10.9	Upper Marker
16		14,499	0.00	0.0	
17		15,440	0.00	0.0	
18		17,096	0.00	0.0	
19		19,245	0.00	0.0	
20		20,096	0.00	0.0	

Region table for sample 8 : 146

From [bp]	To [bp]	Corr. Area	% of Total	Average Size [bp]	Size distribution in CV [%]	Conc. [pg/µl]	Molarity [pmol/l]	Color
200	1,000	22.7	7	437	45.0	6.47	27.6	■

Assay Class: High Sensitivity DNA Assay
Data Path: C:\...gh Sensitivity DNA Assay_DE04105532_2014-12-17_11-51-28.xad

Created: 12/17/2014 11:52:30 AM
Modified: 12/17/2014 12:31:52 PM

Electropherogram Summary Continued ...

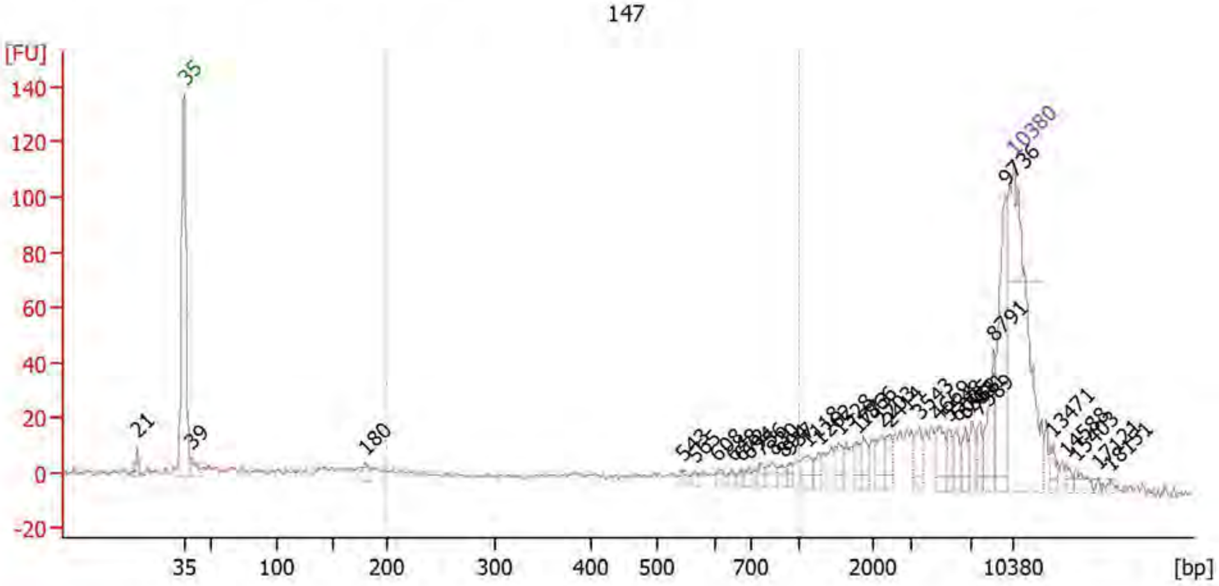

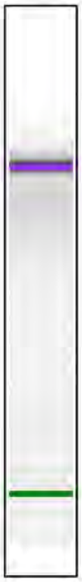

Overall Results for sample 9 : 147

Number of peaks found:	34	Corr. Area 1:	116.2
Noise:	0.9		

Peak table for sample 9 : 147

Peak		Size [bp]	Conc. [pg/µl]	Molarity [pmol/l]	Observations
1		21	0.00	0.0	
2	◀	35	125.00	5,411.3	Lower Marker
3		39	9.05	350.0	
4		180	4.88	41.2	
5		543	1.76	4.9	
6		565	1.79	4.8	
7		608	2.72	6.8	
8		648	1.83	4.3	
9		672	1.97	4.4	
10		694	2.66	5.8	
11		756	2.69	5.4	
12		830	4.45	8.1	
13		894	3.09	5.2	
14		937	2.51	4.1	
15		1,118	5.23	7.1	
16		1,262	3.85	4.6	
17		1,528	4.65	4.6	
18		1,773	4.03	3.4	
19		1,866	5.16	4.2	
20		2,203	6.01	4.1	
21		2,414	5.26	3.3	
22		3,543	5.89	2.5	
23		4,669	8.30	2.7	
24		5,548	4.73	1.3	
25		6,005	5.18	1.3	
26		6,568	4.44	1.0	

Assay Class: High Sensitivity DNA Assay
Data Path: C:\...gh Sensitivity DNA Assay_DE04105532_2014-12-17_11-51-28.xad

Created: 12/17/2014 11:52:30 AM
Modified: 12/17/2014 12:31:52 PM

Electropherogram Summary Continued ...

... Peak table for sample 9 : 147

Peak	Size [bp]	Conc. [pg/µl]	Molarity [pmol/l]	Observations
27	7,031	5.07	1.1	
28	7,589	4.98	1.0	
29	8,791	13.28	2.3	
30	9,736	29.13	4.5	
31	10,380	75.00	10.9	Upper Marker
32	13,471	0.00	0.0	
33	14,588	0.00	0.0	
34	15,403	0.00	0.0	
35	17,121	0.00	0.0	
36	18,151	0.00	0.0	

Region table for sample 9 : 147

From [bp]	To [bp]	Corr. Area	% of Total	Average Size [bp]	Size distribution in CV [%]	Conc. [pg/µl]	Molarity [pmol/l]	Co lor
200	1,000	116.2	19	572	40.1	62.39	225.2	

Assay Class: High Sensitivity DNA Assay
Data Path: C:\...gh Sensitivity DNA Assay_DE04105532_2014-12-17_11-51-28.xad

Created: 12/17/2014 11:52:30 AM
Modified: 12/17/2014 12:31:52 PM

Electropherogram Summary Continued ...

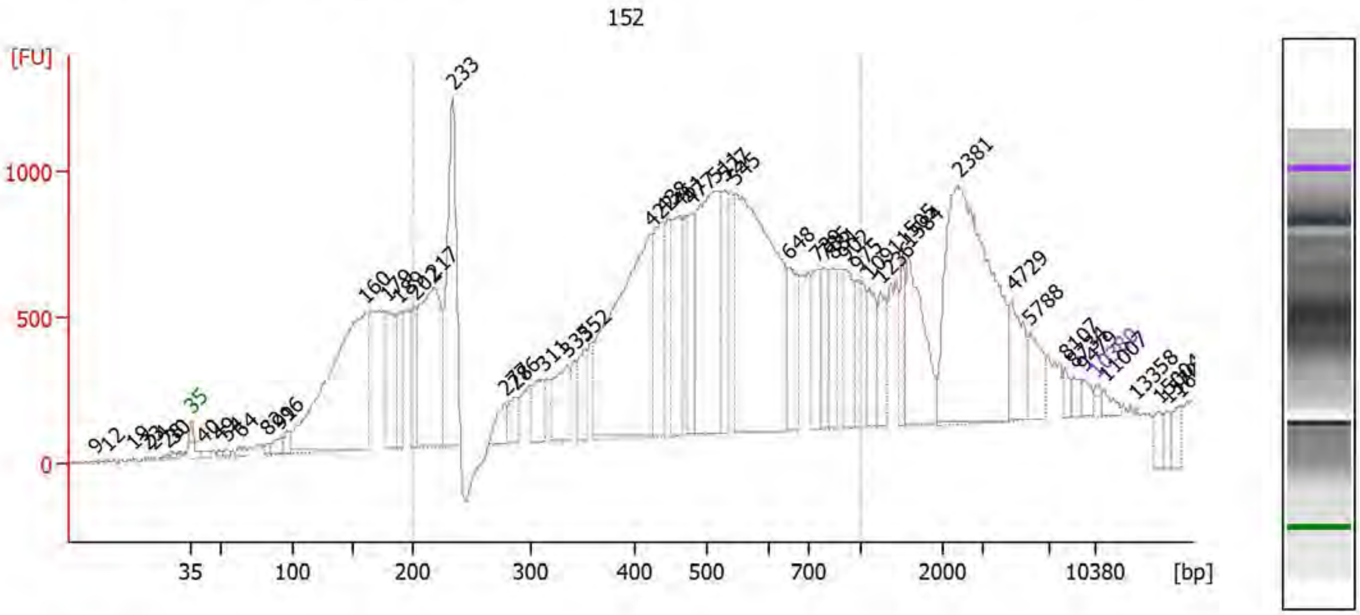

Overall Results for sample 10 : 152

Number of peaks found:	53	Corr. Area 1:	23,006.7
Noise:	7.1		

Peak table for sample 10 : 152

Peak	Size [bp]	Conc. [pg/µl]	Molarity [pmol/l]	Observations
1	9	0.00	0.0	
2	12	0.00	0.0	
3	19	0.00	0.0	
4	23	0.00	0.0	
5	24	0.00	0.0	
6	28	0.00	0.0	
7	30	0.00	0.0	
8	35	125.00	5,411.3	Lower Marker
9	40	112.24	4,207.5	
10	49	72.68	2,243.5	
11	54	70.59	1,983.3	
12	64	132.67	3,163.6	
13	82	120.09	2,208.3	
14	91	242.35	4,023.2	
15	96	244.28	3,837.7	
16	160	7,654.11	72,323.4	
17	179	1,560.19	13,242.4	
18	189	1,022.92	8,213.5	
19	202	1,009.52	7,588.4	
20	217	3,885.80	27,095.2	
21	233	3,537.38	22,956.8	
22	277	418.84	2,286.9	
23	286	444.96	2,360.7	
24	311	608.61	2,967.6	
25	335	1,020.69	4,618.4	
26	352	618.39	2,663.4	

Assay Class: High Sensitivity DNA Assay
Data Path: C:\...gh Sensitivity DNA Assay_DE04105532_2014-12-17_11-51-28.xad

Created: 12/17/2014 11:52:30 AM
Modified: 12/17/2014 12:31:52 PM

Electropherogram Summary Continued ...

... Peak table for sample 10 : 152

Peak		Size [bp]	Conc. [pg/µl]	Molarity [pmol/l]	Observations
27		422	5,529.94	19,868.9	
28		438	1,573.36	5,438.8	
29		461	1,456.59	4,782.9	
30		477	1,042.43	3,314.0	
31		511	3,640.51	10,799.5	
32		527	931.40	2,677.9	
33		545	5,959.95	16,572.2	
34		648	928.87	2,171.2	
35		739	701.96	1,438.5	
36		785	650.26	1,254.7	
37		831	512.05	933.4	
38		902	764.94	1,285.4	
39		975	785.01	1,219.7	
40		1,091	551.24	765.5	
41		1,236	523.60	642.1	
42		1,505	402.45	405.2	
43		1,584	1,300.66	1,244.4	
44		2,381	4,258.71	2,710.4	
45		4,729	641.22	205.4	
46		5,788	466.49	122.1	
47		8,107	112.75	21.1	
48		8,734	142.72	24.8	
49		9,479	118.24	18.9	
50	▶	10,380	75.00	10.9	Upper Marker
51		11,007	0.00	0.0	
52		13,358	0.00	0.0	
53		15,004	0.00	0.0	
54		15,709	0.00	0.0	
55		16,493	0.00	0.0	

Region table for sample 10 : 152

From [bp]	To [bp]	Corr. Area	% of Total	Average Size [bp]	Size distribution in CV [%]	Conc. [pg/µl]	Molarity [pmol/l]	Color
200	1,000	23,006.7	62	495	37.2	39,284.61	153,421.4	■

Assay Class: High Sensitivity DNA Assay
Data Path: C:\...gh Sensitivity DNA Assay_DE04105532_2014-12-17_11-51-28.xad

Created: 12/17/2014 11:52:30 AM
Modified: 12/17/2014 12:31:52 PM

Electropherogram Summary Continued ...

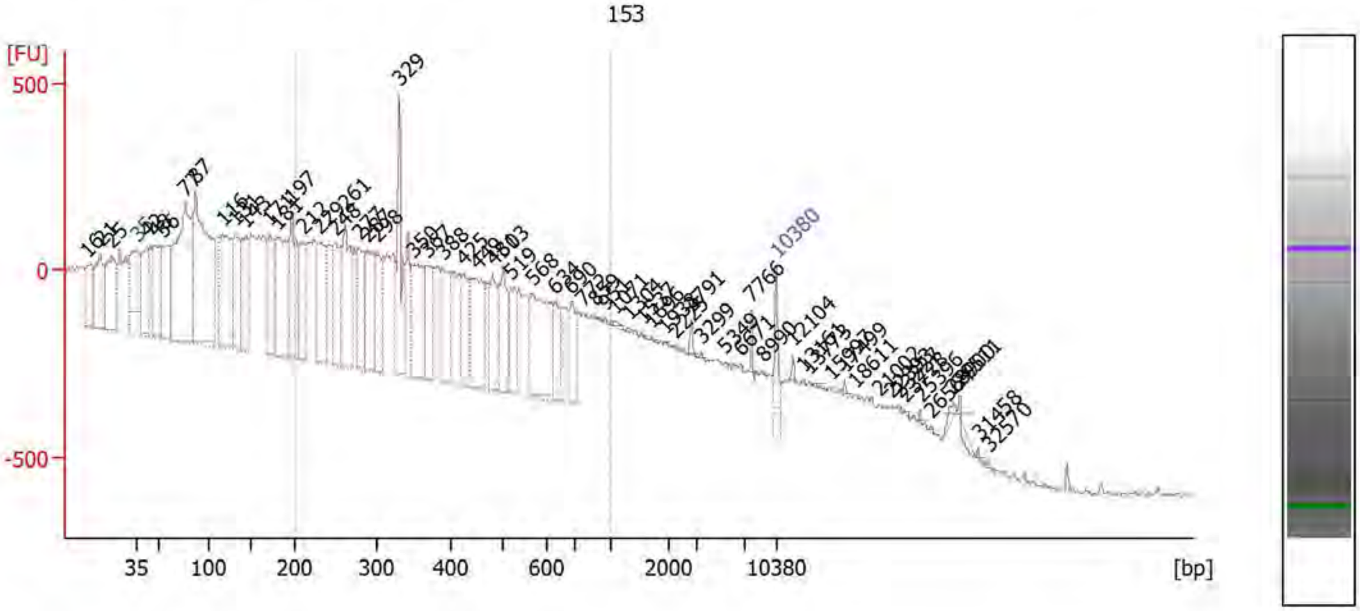

Overall Results for sample 11 : 153

Number of peaks found:	64	Corr. Area 1:	8,607.2
Noise:	6.3		

Peak table for sample 11 : 153

Peak		Size [bp]	Conc. [pg/µl]	Molarity [pmol/l]	Observations
1		16	0.00	0.0	
2		21	0.00	0.0	
3		25	0.00	0.0	
4	◀	35	125.00	5,411.3	Lower Marker
5		42	375.09	13,557.2	
6		48	405.07	12,767.2	
7		56	377.20	10,248.9	
8		77	1,124.00	22,038.0	
9		87	1,218.43	21,159.6	
10		116	562.59	7,318.7	
11		131	358.45	4,137.0	
12		143	323.35	3,425.9	
13		171	304.79	2,698.1	
14		181	571.11	4,791.6	
15		197	424.02	3,262.2	
16		212	308.70	2,210.8	
17		229	320.47	2,116.5	
18		248	324.19	1,981.3	
19		261	365.73	2,120.7	
20		277	216.63	1,183.9	
21		287	291.63	1,537.2	
22		298	234.86	1,193.0	
23		329	327.95	1,508.7	
24		350	319.07	1,382.2	
25		367	219.53	907.5	
26		388	242.96	947.9	

Assay Class: High Sensitivity DNA Assay
Data Path: C:\...gh Sensitivity DNA Assay_DE04105532_2014-12-17_11-51-28.xad

Created: 12/17/2014 11:52:30 AM
Modified: 12/17/2014 12:31:52 PM

Electropherogram Summary Continued ...

... Peak table for sample 11 : 153

Peak	Size [bp]	Conc. [pg/µl]	Molarity [pmol/l]	Observations
27	425	159.76	570.1	
28	449	263.47	888.6	
29	481	204.66	644.7	
30	503	175.91	529.9	
31	519	167.93	490.5	
32	568	391.26	1,042.9	
33	634	106.49	254.6	
34	690	114.63	251.7	
35	772	1.13	2.2	
36	859	4.19	7.4	
37	951	1.99	3.2	
38	1,071	6.31	8.9	
39	1,304	5.43	6.3	
40	1,537	1.86	1.8	
41	1,696	2.22	2.0	
42	1,938	1.04	0.8	
43	2,225	0.83	0.6	
44	2,791	11.98	6.5	
45	3,299	1.97	0.9	
46	5,349	2.26	0.6	
47	6,671	1.65	0.4	
48	7,766	15.32	3.0	
49	8,990	1.58	0.3	
50	10,380	75.00	10.9	Upper Marker
51	12,104	0.00	0.0	
52	13,161	0.00	0.0	
53	13,773	0.00	0.0	
54	15,997	0.00	0.0	
55	17,499	0.00	0.0	
56	18,611	0.00	0.0	
57	21,002	0.00	0.0	
58	22,393	0.00	0.0	
59	23,227	0.00	0.0	
60	23,728	0.00	0.0	
61	25,396	0.00	0.0	
62	26,564	0.00	0.0	
63	28,900	0.00	0.0	
64	29,511	0.00	0.0	
65	31,458	0.00	0.0	
66	32,570	0.00	0.0	

Region table for sample 11 : 153

From [bp]	To [bp]	Corr. Area	% of Total	Average Size [bp]	Size distribution in CV [%]	Conc. [pg/µl]	Molarity [pmol/l]	Co lor
200	1,000	8,607.2	49	432	42.5	4,904.07	21,555.1	

Assay Class: High Sensitivity DNA Assay
Data Path: C:\...gh Sensitivity DNA Assay_DE04105532_2014-12-17_11-51-28.xad

Created: 12/17/2014 11:52:30 AM
Modified: 12/17/2014 12:31:52 PM

Gel Image

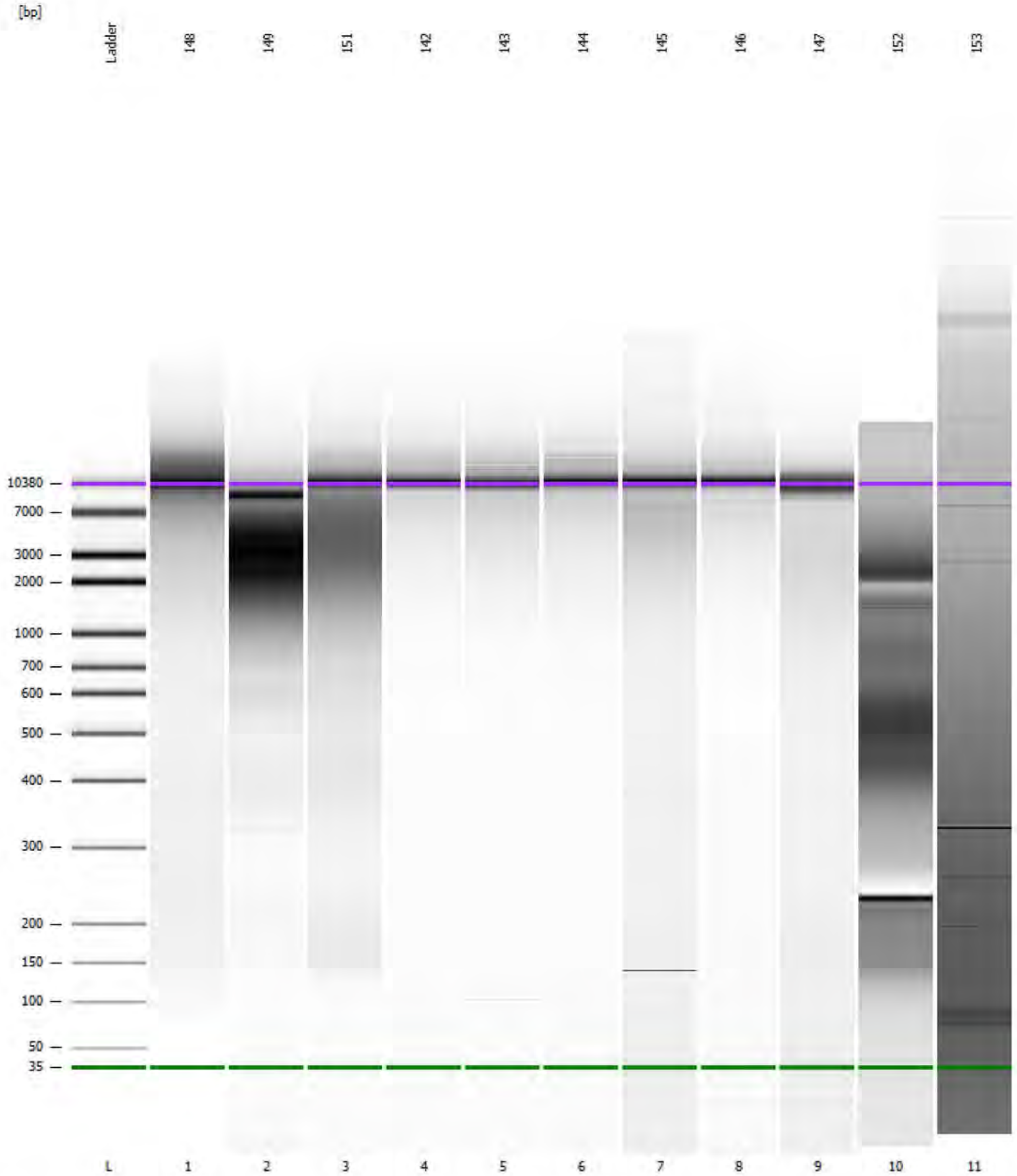

Assay Class: High Sensitivity DNA Assay
Data Path: C:\...gh Sensitivity DNA Assay_DE04105532_2014-12-17_11-51-28.xad

Created: 12/17/2014 11:52:30 AM
Modified: 12/17/2014 12:31:52 PM

Curves

Standard Curve

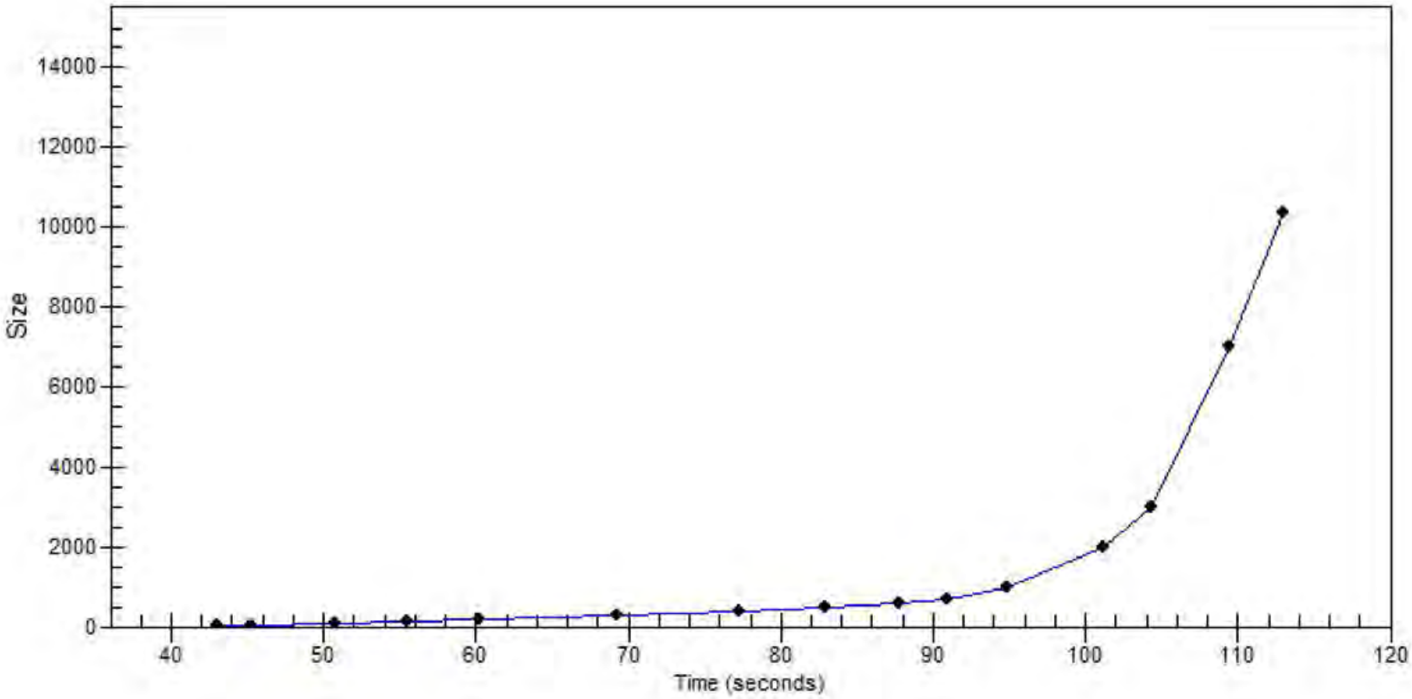

Assay Class: High Sensitivity DNA Assay
Data Path: C:\...gh Sensitivity DNA Assay_DE04105532_2014-12-17_11-51-28.xad

Created: 12/17/2014 11:52:30 AM
Modified: 12/17/2014 12:31:52 PM

Run Logbook

Description	Number	Source	Category	Sub Category	Time	Time Zone	User	Host
Run ended on port 2 (Number of wells acquired: 12)		Instrument	Run		12/17/2014 12:31:52 PM	(GMT +01:00) W. Europe Standard Time	Admin	Datasystem01
Run started on port 2 (File: C:\Program Files\Agilent\2100 bioanalyzer\2100 expert\Data\2014-12-17\2100 expert_High Sensitivity DNA Assay_DE04105532_2014-12-17_11-51-28.xad)		Instrument	Run		12/17/2014 11:52:30 AM	(GMT +01:00) W. Europe Standard Time	Admin	Datasystem01
Product Number : G2938C		Instrument	Run		12/17/2014 11:52:30 AM	(GMT +01:00) W. Europe Standard Time	Admin	Datasystem01
Name :		Instrument	Run		12/17/2014 11:52:30 AM	(GMT +01:00) W. Europe Standard Time	Admin	Datasystem01
Vendor : Agilent Technologies		Instrument	Run		12/17/2014 11:52:30 AM	(GMT +01:00) W. Europe Standard Time	Admin	Datasystem01
Serial# : DE04105532		Instrument	Run		12/17/2014 11:52:30 AM	(GMT +01:00) W. Europe Standard Time	Admin	Datasystem01
Firmware : C.01.069		Instrument	Run		12/17/2014 11:52:30 AM	(GMT +01:00) W. Europe Standard Time	Admin	Datasystem01
Cartridge : Electrode		Instrument	Run		12/17/2014 11:52:30 AM	(GMT +01:00) W. Europe Standard Time	Admin	Datasystem01

Assay Class:	High Sensitivity DNA Assay	Created:	12/18/2014 1:48:26 PM
Data Path:	C:\...gh Sensitivity DNA Assay_DE04105532_2014-12-18_13-48-26.xad	Modified:	12/18/2014 2:29:50 PM

Electrophoresis File Run Summary

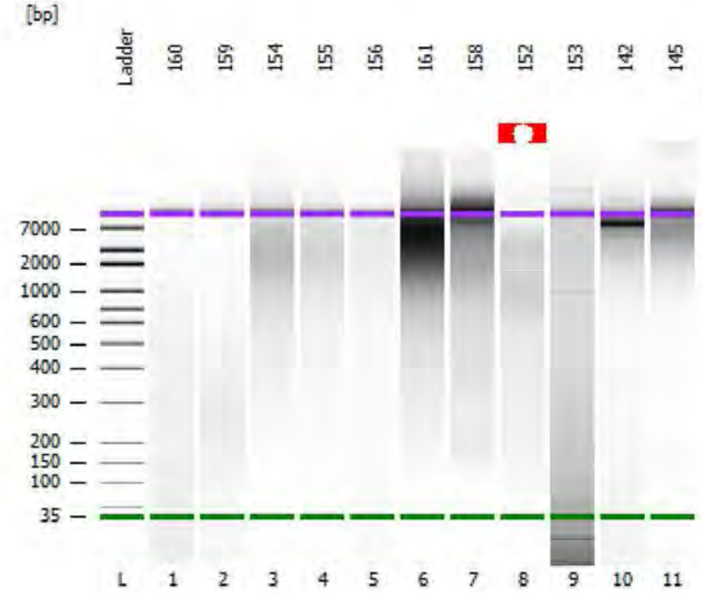

Instrument Information:

Instrument Name:	DE04105532	Firmware:	C.01.069
Serial#:	DE04105532	Type:	G2938C

Assay Information:

Assay Origin Path:	C:\Program Files\Agilent\2100 bioanalyzer\2100 expert\assays\dsDNA\High Sensitivity DNA.xsy
Assay Class:	High Sensitivity DNA Assay
Version:	1.03

160

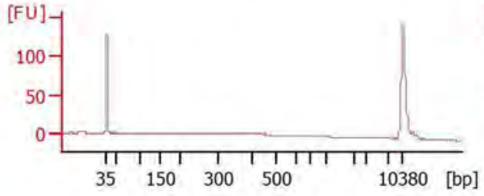

159

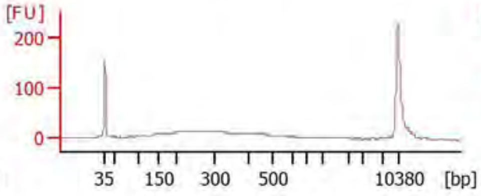

154

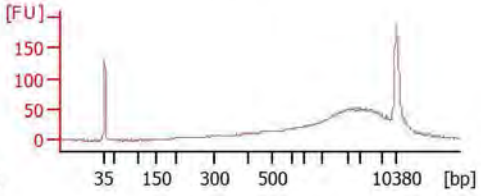

155

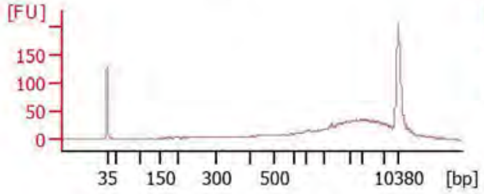

156

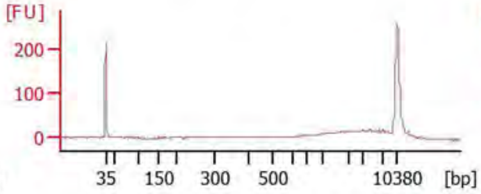

161

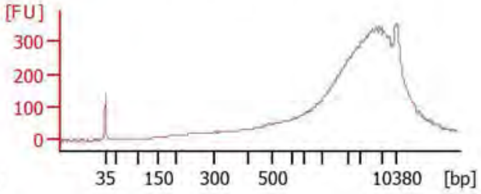

158

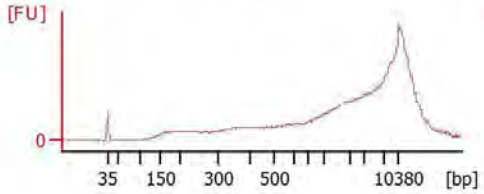

152

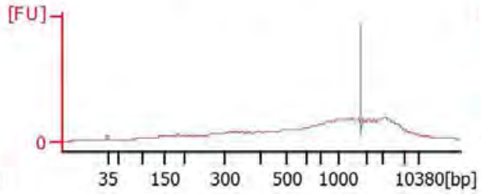

153

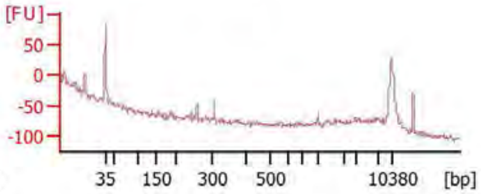

142

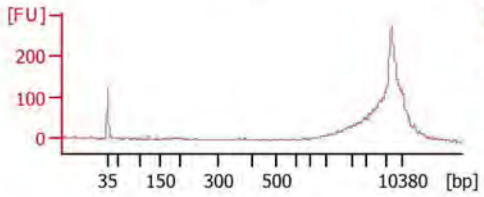

145

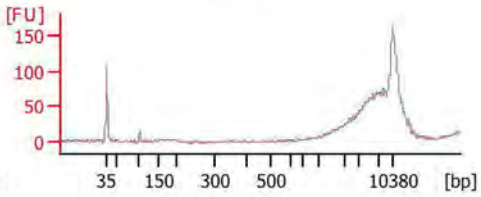

Assay Class: High Sensitivity DNA Assay
Data Path: C:\...gh Sensitivity DNA Assay_DE04105532_2014-12-18_13-48-26.xad

Created: 12/18/2014 1:48:26 PM
Modified: 12/18/2014 2:29:50 PM

Electrophoresis File Run Summary (Chip Summary)

Sample Name	Sample Comment	Rest. Digest	Status	Observation	Result Label	Result Color
160		☐	✔			
159		☐	✔			
154		☐	✔			
155		☐	✔			
156		☐	✔			
161		☐	✔			
158		☐	✔			
152		☐	⚠			
153		☐	✔			
142		☐	✔			
145		☐	✔			
Ladder		☐	✔			

Chip Lot # **Reagent Kit Lot #**

Chip Comments :

Assay Class: High Sensitivity DNA Assay
Data Path: C:\...gh Sensitivity DNA Assay_DE04105532_2014-12-18_13-48-26.xad
Created: 12/18/2014 1:48:26 PM
Modified: 12/18/2014 2:29:50 PM

Electrophoresis Assay Details

General Analysis Settings

Number of Available Sample and Ladder Wells (Max.) : 12
Minimum Visible Range [s] : 32
Maximum Visible Range [s] : 138
Start Analysis Time Range [s] : 33
End Analysis Time Range [s] : 137.5
Ladder Concentration [pg/µl] : 1950
Uses Standard Area for Ladder Fragments
Lower Marker Concentration [pg/µl] : 125
Upper Marker Concentration [pg/µl] : 75
Used Upper Marker for Quantitation
Standard Curve Fit is Point to Point
Show Data Aligned to Lower and Upper Marker

Integrator Settings

Integration Start Time [s] : 33.05
Integration End Time [s] : 137
Slope Threshold : 0.8
Height Threshold [FU] : 5
Area Threshold : 0.1
Width Threshold [s] : 0.6
Baseline Plateau [s] : 0.5

Filter Settings

Filter Width [s] : 0.5
Polynomial Order : 4

Ladder

Ladder Peak	Size	Area
1	35	160
2	50	210
3	100	208
4	150	221
5	200	242
6	300	270
7	400	305
8	500	306
9	600	336
10	700	321
11	1000	366
12	2000	413
13	3000	411
14	7000	400
15	10380	214

Assay Class: High Sensitivity DNA Assay
Data Path: C:\...gh Sensitivity DNA Assay_DE04105532_2014-12-18_13-48-26.xad
Created: 12/18/2014 1:48:26 PM
Modified: 12/18/2014 2:29:50 PM

Electropherogram Summary

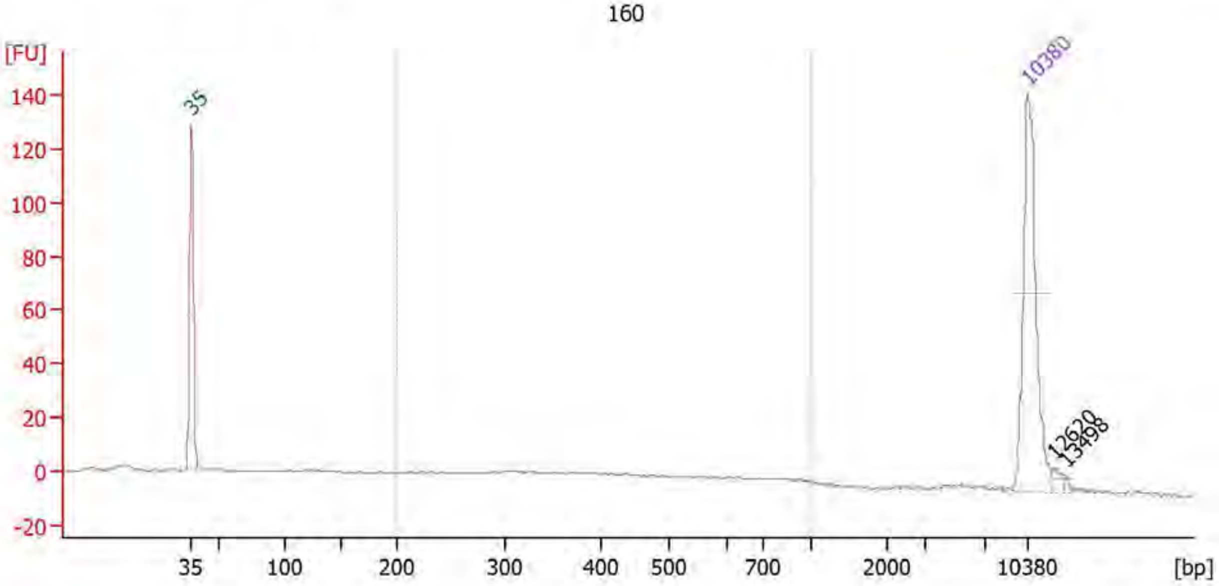

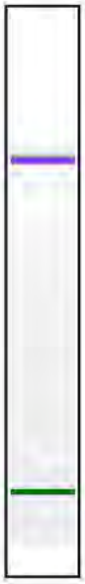

Overall Results for sample 1 : 160

Number of peaks found:	2	Corr. Area 1:	144.7
Noise:	0.3		

Peak table for sample 1 : 160

Peak		Size [bp]	Conc. [pg/µl]	Molarity [pmol/l]	Observations
1	◀	35	125.00	5,411.3	Lower Marker
2	▶	10,380	75.00	10.9	Upper Marker
3		12,620	0.00	0.0	
4		13,498	0.00	0.0	

Region table for sample 1 : 160

From [bp]	To [bp]	Corr. Area	% of Total	Average Size [bp]	Size distribution in CV [%]	Conc. [pg/µl]	Molarity [pmol/l]	Co lor
200	1,000	144.7	52	456	41.3	95.83	399.0	■

Assay Class: High Sensitivity DNA Assay
Data Path: C:\...gh Sensitivity DNA Assay_DE04105532_2014-12-18_13-48-26.xad

Created: 12/18/2014 1:48:26 PM
Modified: 12/18/2014 2:29:50 PM

Electropherogram Summary Continued ...

159

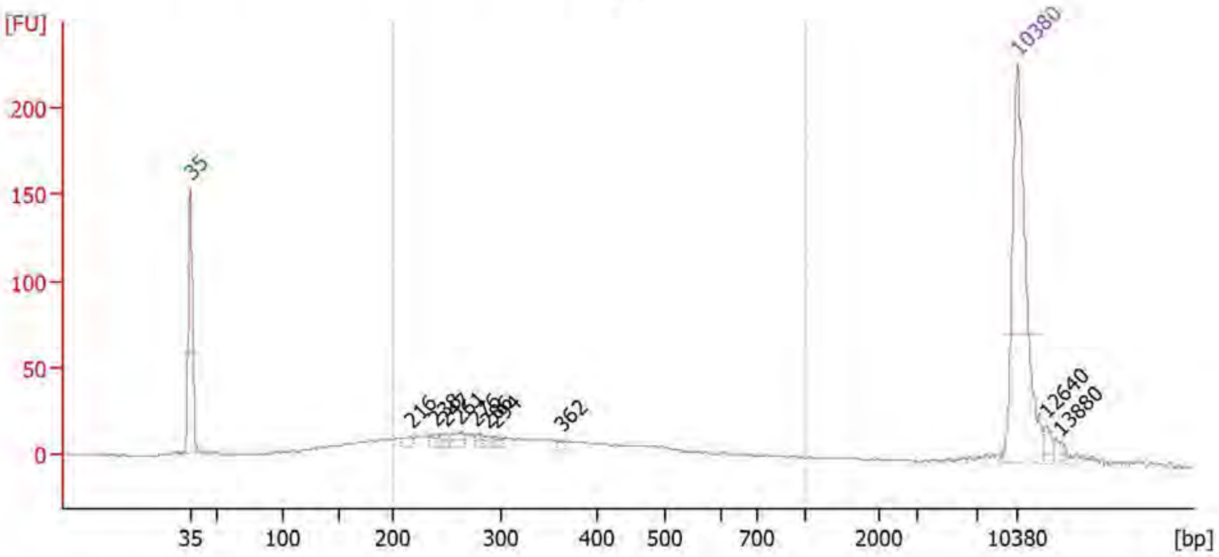

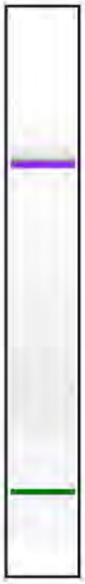

Overall Results for sample 2 : 159

Number of peaks found:	10	Corr. Area 1:	436.3
Noise:	0.4		

Peak table for sample 2 : 159

Peak		Size [bp]	Conc. [pg/µl]	Molarity [pmol/l]	Observations
1	◀	35	125.00	5,411.3	Lower Marker
2		216	4.64	32.6	
3		238	5.12	32.5	
4		247	4.62	28.3	
5		261	7.54	43.7	
6		276	4.50	24.6	
7		286	2.94	15.6	
8		294	3.53	18.2	
9		362	2.24	9.4	
10	▶	10,380	75.00	10.9	Upper Marker
11		12,640	0.00	0.0	
12		13,880	0.00	0.0	

Region table for sample 2 : 159

From [bp]	To [bp]	Corr. Area	% of Total	Average Size [bp]	Size distribution in CV [%]	Conc. [pg/µl]	Molarity [pmol/l]	Color
200	1,000	436.3	66	375	40.1	186.64	899.5	■

Assay Class: High Sensitivity DNA Assay
Data Path: C:\...gh Sensitivity DNA Assay_DE04105532_2014-12-18_13-48-26.xad

Created: 12/18/2014 1:48:26 PM
Modified: 12/18/2014 2:29:50 PM

Electropherogram Summary Continued ...

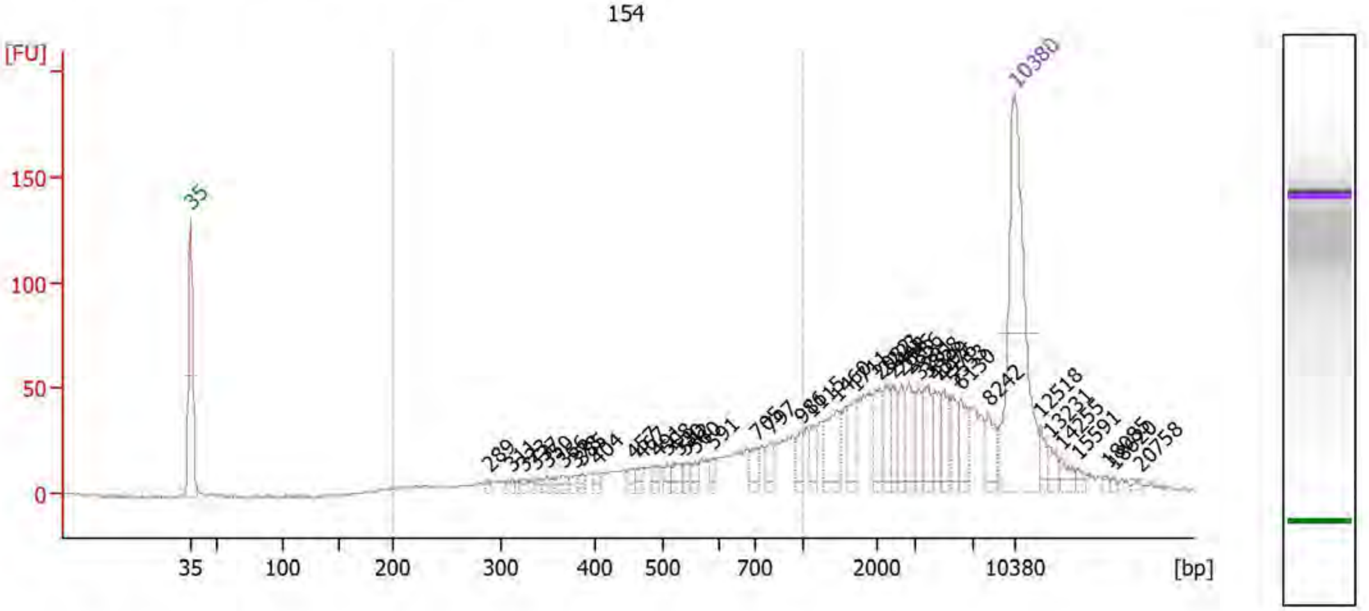

Overall Results for sample 3 : 154

Number of peaks found: 42
Corr. Area 1: 446.8
Noise: 0.4

Peak table for sample 3 : 154

Peak	Size [bp]	Conc. [pg/µl]	Molarity [pmol/l]	Observations
1	35	125.00	5,411.3	Lower Marker
2	289	2.25	11.8	
3	311	2.96	14.4	
4	323	3.24	15.2	
5	337	2.96	13.3	
6	350	3.23	14.0	
7	366	5.07	21.0	
8	378	3.41	13.6	
9	385	3.01	11.8	
10	404	3.18	11.9	
11	457	4.64	15.4	
12	467	3.76	12.2	
13	491	4.71	14.5	
14	508	3.97	11.8	
15	530	5.66	16.2	
16	542	3.95	11.1	
17	560	3.63	9.8	
18	591	3.98	10.2	
19	705	7.11	15.3	
20	797	6.55	12.5	
21	986	6.29	9.7	
22	1,115	6.79	9.2	
23	1,460	17.64	18.3	
24	1,711	9.55	8.5	
25	2,082	12.07	8.8	
26	2,223	9.81	6.7	

Assay Class: High Sensitivity DNA Assay
Data Path: C:\...gh Sensitivity DNA Assay_DE04105532_2014-12-18_13-48-26.xad

Created: 12/18/2014 1:48:26 PM
Modified: 12/18/2014 2:29:50 PM

Electropherogram Summary Continued ...

... Peak table for sample 3 : 154

Peak		Size [bp]	Conc. [pg/µl]	Molarity [pmol/l]	Observations
27		2,448	8.38	5.2	
28		2,645	8.94	5.1	
29		2,856	12.11	6.4	
30		3,281	8.07	3.7	
31		3,858	11.23	4.4	
32		4,399	10.83	3.7	
33		4,976	8.29	2.5	
34		5,553	8.42	2.3	
35		6,130	10.05	2.5	
36		8,242	9.26	1.7	
37	▶	10,380	75.00	10.9	Upper Marker
38		12,518	0.00	0.0	
39		13,231	0.00	0.0	
40		14,255	0.00	0.0	
41		15,591	0.00	0.0	
42		18,085	0.00	0.0	
43		18,620	0.00	0.0	
44		20,758	0.00	0.0	

Region table for sample 3 : 154

From [bp]	To [bp]	Corr. Area	% of Total	Average Size [bp]	Size distribution in CV [%]	Conc. [pg/µl]	Molarity [pmol/l]	Color
200	1,000	446.8	37	577	35.7	172.78	564.9	■

Assay Class: High Sensitivity DNA Assay
Data Path: C:\...gh Sensitivity DNA Assay_DE04105532_2014-12-18_13-48-26.xad

Created: 12/18/2014 1:48:26 PM
Modified: 12/18/2014 2:29:50 PM

Electropherogram Summary Continued ...

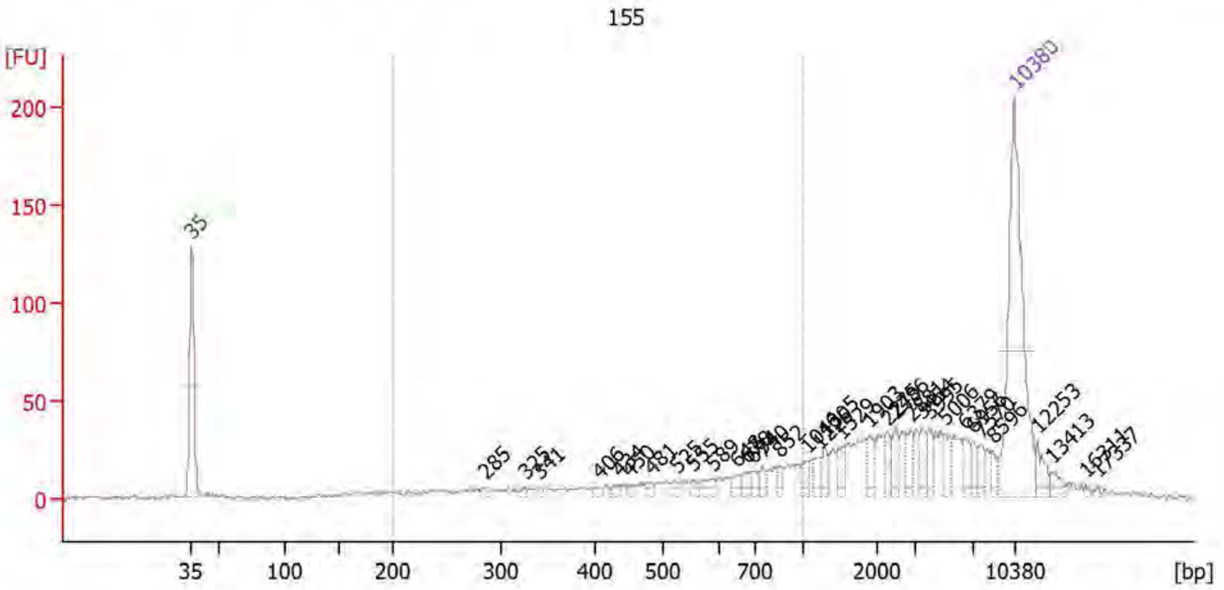

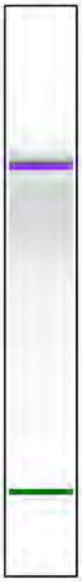

Overall Results for sample 4 : 155

Number of peaks found:	34	Corr. Area 1:	308.2
Noise:	0.8		

Peak table for sample 4 : 155

Peak	Size [bp]	Conc. [pg/µl]	Molarity [pmol/l]	Observations
1	35	125.00	5,411.3	Lower Marker
2	285	2.08	11.0	
3	325	2.18	10.2	
4	341	1.87	8.3	
5	406	2.47	9.2	
6	434	3.00	10.5	
7	450	2.05	6.9	
8	481	3.02	9.5	
9	525	3.16	9.1	
10	555	2.80	7.6	
11	589	6.00	15.4	
12	648	4.31	10.1	
13	678	4.70	10.5	
14	694	3.90	8.5	
15	740	4.08	8.3	
16	852	4.11	7.3	
17	1,046	4.63	6.7	
18	1,226	5.69	7.0	
19	1,305	5.35	6.2	
20	1,529	6.34	6.3	
21	1,903	6.30	5.0	
22	2,275	5.93	3.9	
23	2,486	7.08	4.3	
24	2,881	7.00	3.7	
25	3,454	6.21	2.7	
26	3,995	6.12	2.3	

Assay Class: High Sensitivity DNA Assay
Data Path: C:\...gh Sensitivity DNA Assay_DE04105532_2014-12-18_13-48-26.xad

Created: 12/18/2014 1:48:26 PM
Modified: 12/18/2014 2:29:50 PM

Electropherogram Summary Continued ...

... Peak table for sample 4 : 155

Peak		Size [bp]	Conc. [pg/µl]	Molarity [pmol/l]	Observations
27		5,006	6.55	2.0	
28		6,379	6.00	1.4	
29		6,956	4.91	1.1	
30		7,570	4.56	0.9	
31		8,596	4.07	0.7	
32	▶	10,380	75.00	10.9	Upper Marker
33		12,253	0.00	0.0	
34		13,413	0.00	0.0	
35		16,311	0.00	0.0	
36		17,337	0.00	0.0	

Region table for sample 4 : 155

From [bp]	To [bp]	Corr. Area	% of Total	Average Size [bp]	Size distribution in CV [%]	Conc. [pg/µl]	Molarity [pmol/l]	Color
200	1,000	308.2	35	564	37.9	129.56	447.9	

Assay Class: High Sensitivity DNA Assay
Data Path: C:\...gh Sensitivity DNA Assay_DE04105532_2014-12-18_13-48-26.xad
Created: 12/18/2014 1:48:26 PM
Modified: 12/18/2014 2:29:50 PM

Electropherogram Summary Continued ...

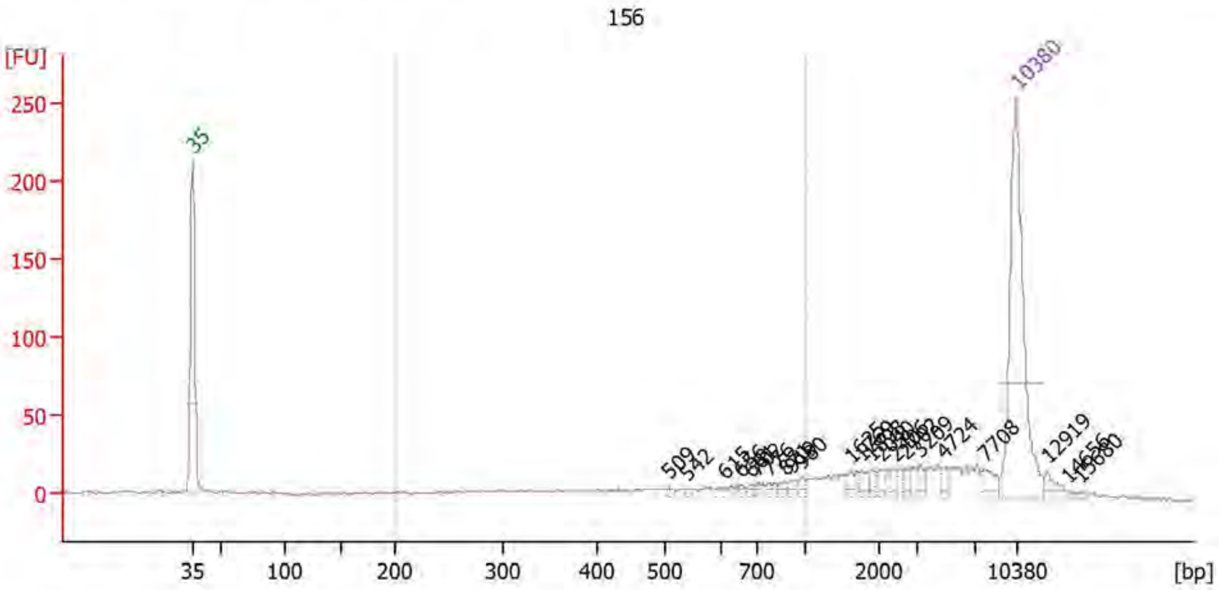

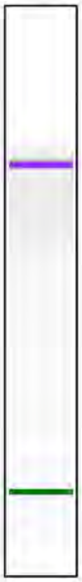

Overall Results for sample 5 : 156

Number of peaks found: 22
Noise: 0.8
Corr. Area 1: 193.3

Peak table for sample 5 : 156

Peak		Size [bp]	Conc. [pg/µl]	Molarity [pmol/l]	Observations
1	◀	35	125.00	5,411.3	Lower Marker
2		509	1.36	4.1	
3		542	1.37	3.8	
4		615	2.64	6.5	
5		656	2.24	5.2	
6		681	1.71	3.8	
7		702	1.79	3.9	
8		776	1.81	3.5	
9		871	2.27	3.9	
10		919	2.90	4.8	
11		980	2.49	3.8	
12		1,625	3.60	3.4	
13		1,769	3.49	3.0	
14		1,898	2.68	2.1	
15		2,040	2.75	2.0	
16		2,406	3.44	2.2	
17		2,702	3.71	2.1	
18		3,209	2.92	1.4	
19		4,724	3.40	1.1	
20		7,708	6.44	1.3	
21	▶	10,380	75.00	10.9	Upper Marker
22		12,919	0.00	0.0	
23		14,656	0.00	0.0	
24		15,680	0.00	0.0	

Assay Class: High Sensitivity DNA Assay
Data Path: C:\...gh Sensitivity DNA Assay_DE04105532_2014-12-18_13-48-26.xad

Created: 12/18/2014 1:48:26 PM
Modified: 12/18/2014 2:29:50 PM

Electropherogram Summary Continued ...

... Region table for sample 5 : 156

From [bp]	To [bp]	Corr. Area	% of Total	Average Size [bp]	Size distribution in CV [%]	Conc. [pg/µl]	Molarity [pmol/l]	Co lor
200	1,000	193.3	35	559	38.5	66.65	231.4	

Assay Class: High Sensitivity DNA Assay
Data Path: C:\...gh Sensitivity DNA Assay_DE04105532_2014-12-18_13-48-26.xad

Created: 12/18/2014 1:48:26 PM
Modified: 12/18/2014 2:29:50 PM

Electropherogram Summary Continued ...

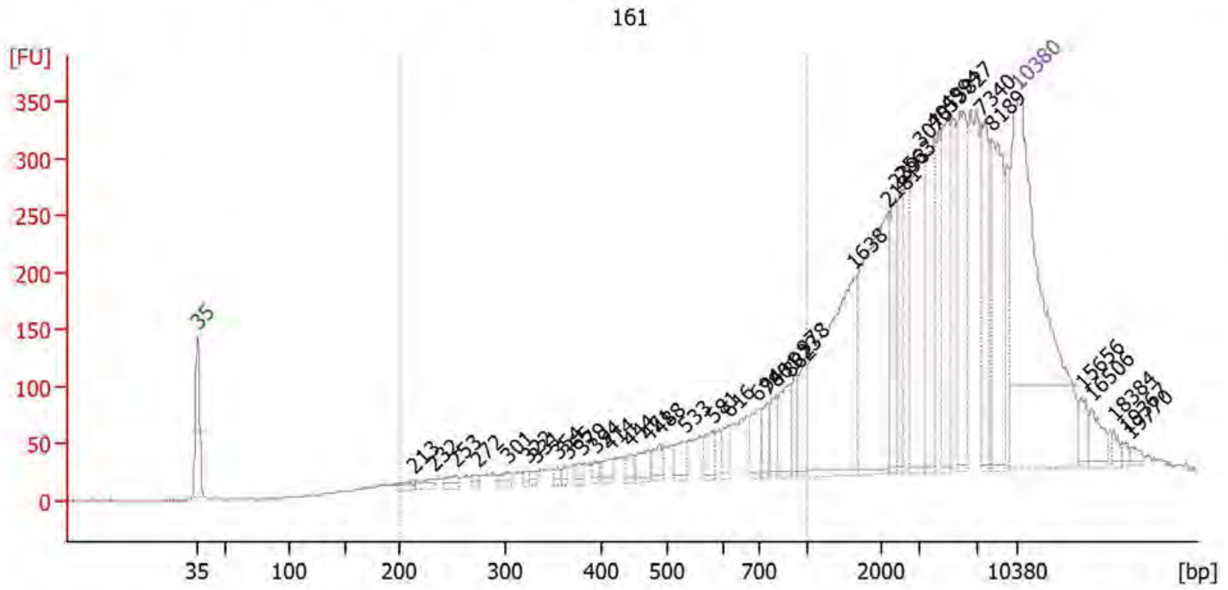

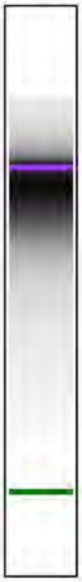

Overall Results for sample 6 : __161__

Number of peaks found:	39	Corr. Area 1:	1,318.9
Noise:	0.6		

Peak table for sample 6 : __161__

Peak	Size [bp]	Conc. [pg/µl]	Molarity [pmol/l]	Observations
1	35	125.00	5,411.3	Lower Marker
2	213	2.40	17.0	
3	232	3.28	21.5	
4	253	3.10	18.5	
5	272	1.75	9.7	
6	301	2.58	13.0	
7	322	1.41	6.6	
8	331	1.52	7.0	
9	354	1.71	7.3	
10	365	1.50	6.2	
11	379	2.06	8.2	
12	394	2.12	8.2	
13	414	3.23	11.8	
14	444	2.75	9.4	
15	471	4.99	16.0	
16	488	4.26	13.2	
17	533	5.40	15.3	
18	581	5.37	14.0	
19	616	4.35	10.7	
20	694	7.55	16.5	
21	746	5.22	10.6	
22	801	5.88	11.1	
23	882	9.39	16.1	
24	923	5.58	9.2	
25	978	7.77	12.0	
26	1,638	53.25	49.3	

Assay Class: High Sensitivity DNA Assay
Data Path: C:\...gh Sensitivity DNA Assay_DE04105532_2014-12-18_13-48-26.xad

Created: 12/18/2014 1:48:26 PM
Modified: 12/18/2014 2:29:50 PM

Electropherogram Summary Continued ...

... Peak table for sample 6 : 161

Peak	Size [bp]	Conc. [pg/µl]	Molarity [pmol/l]	Observations
27	2,181	48.76	33.9	
28	2,393	12.79	8.1	
29	2,563	12.39	7.3	
30	3,075	27.70	13.6	
31	4,053	22.61	8.5	
32	4,994	21.98	6.7	
33	5,827	24.68	6.4	
34	7,340	20.00	4.1	
35	8,189	28.43	5.3	
36	10,380	75.00	10.9	Upper Marker
37	15,656	0.00	0.0	
38	16,506	0.00	0.0	
39	18,384	0.00	0.0	
40	19,367	0.00	0.0	
41	19,770	0.00	0.0	

Region table for sample 6 : 161

From [bp]	To [bp]	Corr. Area	% of Total	Average Size [bp]	Size distribution in CV [%]	Conc. [pg/µl]	Molarity [pmol/l]	Color
200	1,000	1,318.9	24	596	36.1	149.50	483.9	

Assay Class: High Sensitivity DNA Assay
Data Path: C:\...gh Sensitivity DNA Assay_DE04105532_2014-12-18_13-48-26.xad
Created: 12/18/2014 1:48:26 PM
Modified: 12/18/2014 2:29:50 PM

Electropherogram Summary Continued ...

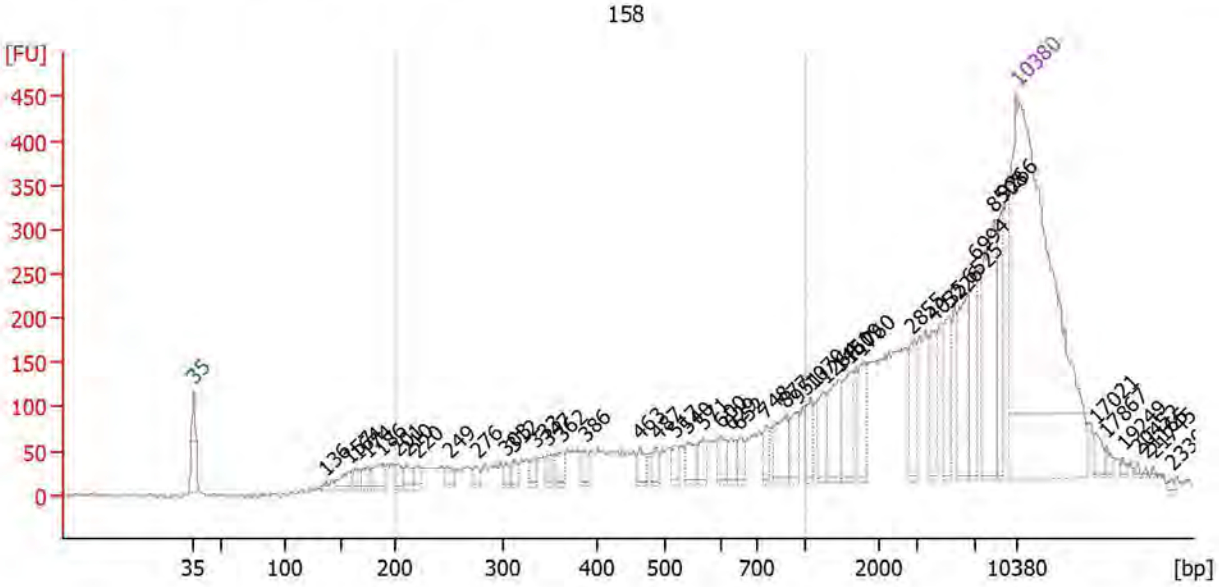

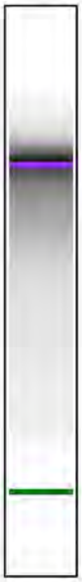

Overall Results for sample 7 : 158

Number of peaks found: 46
Corr. Area 1: 1,904.3
Noise: 2.0

Peak table for sample 7 : 158

Peak	Size [bp]	Conc. [pg/µl]	Molarity [pmol/l]	Observations
1	35	125.00	5,411.3	Lower Marker
2	136	0.99	11.1	
3	157	4.11	39.6	
4	164	2.75	25.5	
5	174	3.49	30.3	
6	186	4.75	38.8	
7	201	2.98	22.4	
8	210	3.25	23.4	
9	220	2.14	14.7	
10	249	2.68	16.3	
11	276	1.97	10.8	
12	303	1.75	8.8	
13	312	2.04	9.9	
14	333	2.40	10.9	
15	347	2.20	9.6	
16	362	3.19	13.4	
17	386	2.25	8.8	
18	463	2.88	9.4	
19	487	2.30	7.1	
20	517	2.47	7.2	
21	540	3.83	10.7	
22	571	3.41	9.1	
23	600	3.44	8.7	
24	629	2.71	6.5	
25	652	2.56	5.9	
26	748	2.65	5.4	

Assay Class: High Sensitivity DNA Assay
Data Path: C:\...gh Sensitivity DNA Assay_DE04105532_2014-12-18_13-48-26.xad

Created: 12/18/2014 1:48:26 PM
Modified: 12/18/2014 2:29:50 PM

Electropherogram Summary Continued ...

... Peak table for sample 7 : 158

Peak	Size [bp]	Conc. [pg/µl]	Molarity [pmol/l]	Observations
27	877	6.28	10.8	
28	951	4.33	6.9	
29	1,070	4.32	6.1	
30	1,264	5.35	6.4	
31	1,451	7.76	8.1	
32	1,609	6.40	6.0	
33	1,760	5.56	4.8	
34	2,855	5.76	3.1	
35	4,035	5.74	2.2	
36	5,226	6.64	1.9	
37	6,525	10.06	2.3	
38	6,994	8.19	1.8	
39	8,508	16.90	3.0	
40	9,266	8.23	1.3	
41	10,380	75.00	10.9	Upper Marker
42	17,021	0.00	0.0	
43	17,867	0.00	0.0	
44	19,249	0.00	0.0	
45	20,452	0.00	0.0	
46	21,165	0.00	0.0	
47	21,745	0.00	0.0	
48	23,305	0.00	0.0	

Region table for sample 7 : 158

From [bp]	To [bp]	Corr. Area	% of Total	Average Size [bp]	Size distribution in CV [%]	Conc. [pg/µl]	Molarity [pmol/l]	Co lor
200	1,000	1,904.3	36	518	41.2	135.40	516.3	

Assay Class: High Sensitivity DNA Assay
Data Path: C:\...gh Sensitivity DNA Assay_DE04105532_2014-12-18_13-48-26.xad

Created: 12/18/2014 1:48:26 PM
Modified: 12/18/2014 2:29:50 PM

Electropherogram Summary Continued ...

152

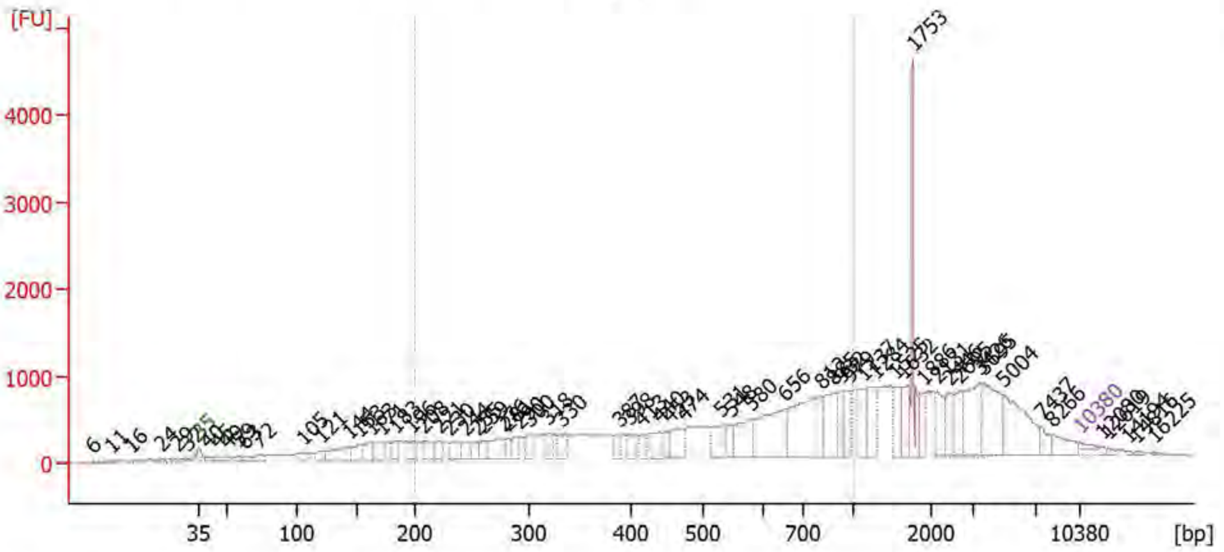

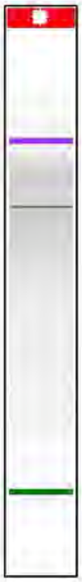

Overall Results for sample 8 : 152

Number of peaks found:	65	Corr. Area 1:	17,016.9
Noise:	7.4		

Peak table for sample 8 : 152

Peak	Size [bp]	Conc. [pg/µl]	Molarity [pmol/l]	Observations
1	6	0.00	0.0	
2	11	0.00	0.0	
3	16	0.00	0.0	
4	24	0.00	0.0	
5	29	0.00	0.0	
6	32	0.00	0.0	
7	35	125.00	5,411.3	Lower Marker
8	40	68.95	2,632.2	
9	45	81.75	2,756.5	
10	49	70.91	2,177.0	
11	59	65.64	1,697.2	
12	63	84.45	2,038.5	
13	72	107.38	2,251.4	
14	105	123.14	1,778.9	
15	121	128.38	1,613.9	
16	144	423.61	4,464.1	
17	153	220.98	2,191.7	
18	163	216.49	2,014.9	
19	171	270.59	2,391.4	
20	183	164.40	1,359.0	
21	196	176.44	1,363.7	
22	208	253.18	1,848.3	
23	221	133.97	918.7	
24	230	233.78	1,541.9	
25	244	155.35	966.3	
26	255	131.46	782.1	

Assay Class: High Sensitivity DNA Assay
Data Path: C:\...gh Sensitivity DNA Assay_DE04105532_2014-12-18_13-48-26.xad
Created: 12/18/2014 1:48:26 PM
Modified: 12/18/2014 2:29:50 PM

Electropherogram Summary Continued ...

... Peak table for sample 8 : 152

Peak		Size [bp]	Conc. [pg/µl]	Molarity [pmol/l]	Observations
27		259	129.73	758.1	
28		276	314.88	1,729.0	
29		281	131.26	707.7	
30		290	143.79	750.6	
31		300	152.26	769.1	
32		318	154.50	736.7	
33		330	190.37	873.3	
34		387	126.87	496.4	
35		398	149.81	570.0	
36		413	112.65	413.2	
37		440	189.40	652.5	
38		453	121.03	404.7	
39		474	282.75	904.0	
40		531	231.09	659.6	
41		548	141.45	391.4	
42		580	428.02	1,117.3	
43		656	756.35	1,747.9	
44		812	1,018.52	1,899.7	
45		885	416.74	713.6	
46		932	210.68	342.4	
47		979	224.91	347.9	
48		1,137	287.70	383.3	
49		1,284	349.21	412.0	
50		1,525	199.00	197.7	
51		1,632	208.84	193.9	
52		1,753	513.24	443.7	
53		1,886	167.05	134.2	
54		2,131	221.33	157.3	
55		2,446	195.57	121.2	
56		2,695	224.22	126.1	
57		3,426	431.96	191.0	
58		3,695	545.42	223.7	
59		5,004	578.93	175.3	
60		7,437	102.49	20.9	
61		8,266	146.59	26.9	
62	▶	10,380	75.00	10.9	Upper Marker
63		12,080	0.00	0.0	
64		12,619	0.00	0.0	
65		14,194	0.00	0.0	
66		14,816	0.00	0.0	
67		16,225	0.00	0.0	

Region table for sample 8 : 152

From [bp]	To [bp]	Corr. Area	% of Total	Average Size [bp]	Size distribution in CV [%]	Conc. [pg/µl]	Molarity [pmol/l]	Co lor
200	1,000	17,016.9	49	535	40.7	9,182.99	34,194.6	■

Assay Class: High Sensitivity DNA Assay
Data Path: C:\...gh Sensitivity DNA Assay_DE04105532_2014-12-18_13-48-26.xad

Created: 12/18/2014 1:48:26 PM
Modified: 12/18/2014 2:29:50 PM

Electropherogram Summary Continued ...

153

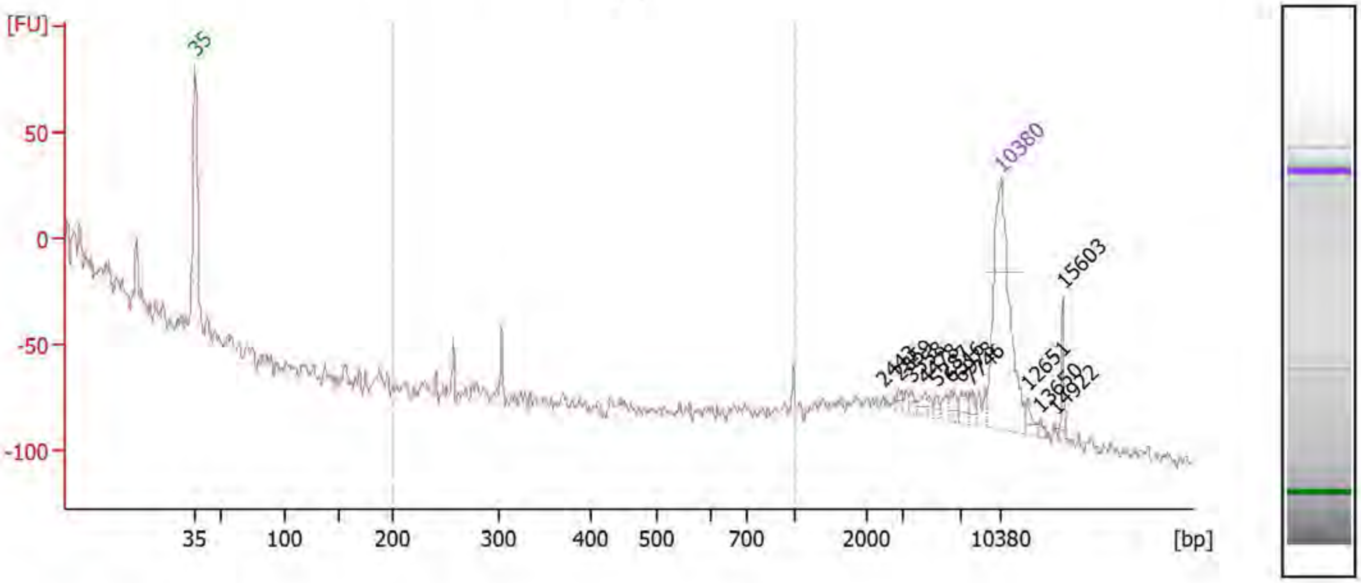

Overall Results for sample 9 : 153

Number of peaks found:	12	Corr. Area 1:	0.6
Noise:	3.5		

Peak table for sample 9 : 153

Peak		Size [bp]	Conc. [pg/µl]	Molarity [pmol/l]	Observations
1	◀	35	125.00	5,411.3	Lower Marker
2		2,443	0.75	0.5	
3		2,859	2.51	1.3	
4		3,558	2.49	1.1	
5		4,478	3.44	1.2	
6		5,287	2.46	0.7	
7		6,316	4.31	1.0	
8		6,978	4.81	1.0	
9		7,746	4.04	0.8	
10	▶	10,380	75.00	10.9	Upper Marker
11		12,651	0.00	0.0	
12		13,650	0.00	0.0	
13		14,922	0.00	0.0	
14		15,603	0.00	0.0	

Region table for sample 9 : 153

From [bp]	To [bp]	Corr. Area	% of Total	Average Size [bp]	Size distribution in CV [%]	Conc. [pg/µl]	Molarity [pmol/l]	Color
200	1,000	0.6	1	995	0.2	0.28	0.4	■

Assay Class: High Sensitivity DNA Assay
Data Path: C:\...gh Sensitivity DNA Assay_DE04105532_2014-12-18_13-48-26.xad

Created: 12/18/2014 1:48:26 PM
Modified: 12/18/2014 2:29:50 PM

Electropherogram Summary Continued ...

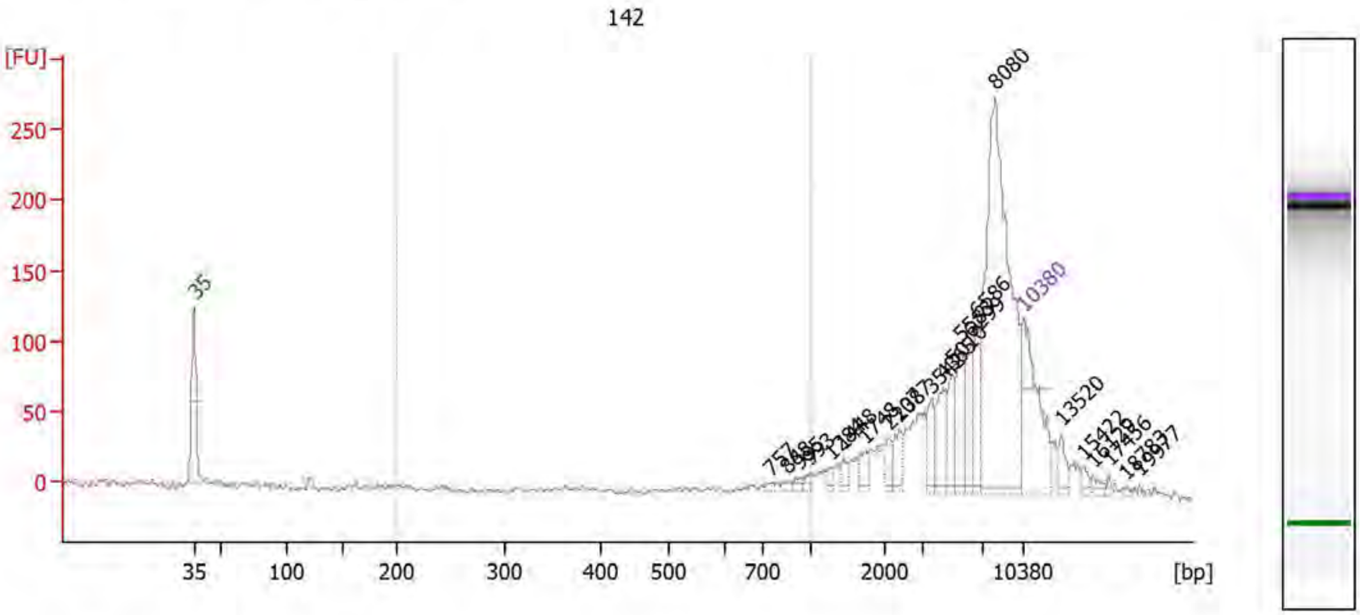

Overall Results for sample 10 : 142

Number of peaks found:	22	Corr. Area 1:	46.9
Noise:	2.3		

Peak table for sample 10 : 142

Peak		Size [bp]	Conc. [pg/µl]	Molarity [pmol/l]	Observations
1	◀	35	125.00	5,411.3	Lower Marker
2		757	2.51	5.0	
3		848	3.93	7.0	
4		935	4.30	7.0	
5		993	3.83	5.8	
6		1,284	5.09	6.0	
7		1,448	7.14	7.5	
8		1,748	10.45	9.1	
9		2,107	9.23	6.6	
10		2,387	14.76	9.4	
11		3,542	19.68	8.4	
12		4,365	28.68	10.0	
13		5,010	23.91	7.2	
14		5,583	29.81	8.1	
15		6,299	27.92	6.7	
16		6,586	27.55	6.3	
17		8,080	255.79	48.0	
18	▶	10,380	75.00	10.9	Upper Marker
19		13,520	0.00	0.0	
20		15,422	0.00	0.0	
21		16,129	0.00	0.0	
22		17,456	0.00	0.0	
23		18,783	0.00	0.0	
24		19,977	0.00	0.0	

Assay Class: High Sensitivity DNA Assay
Data Path: C:\...gh Sensitivity DNA Assay_DE04105532_2014-12-18_13-48-26.xad

Created: 12/18/2014 1:48:26 PM
Modified: 12/18/2014 2:29:50 PM

Electropherogram Summary Continued ...

... Region table for sample 10 : 142

From [bp]	To [bp]	Corr. Area	% of Total	Average Size [bp]	Size distribution in CV [%]	Conc. [pg/µl]	Molarity [pmol/l]	Color
200	1,000	46.9	3	782	22.2	24.48	53.1	

Assay Class: High Sensitivity DNA Assay
Data Path: C:\...gh Sensitivity DNA Assay_DE04105532_2014-12-18_13-48-26.xad

Created: 12/18/2014 1:48:26 PM
Modified: 12/18/2014 2:29:50 PM

Electropherogram Summary Continued ...

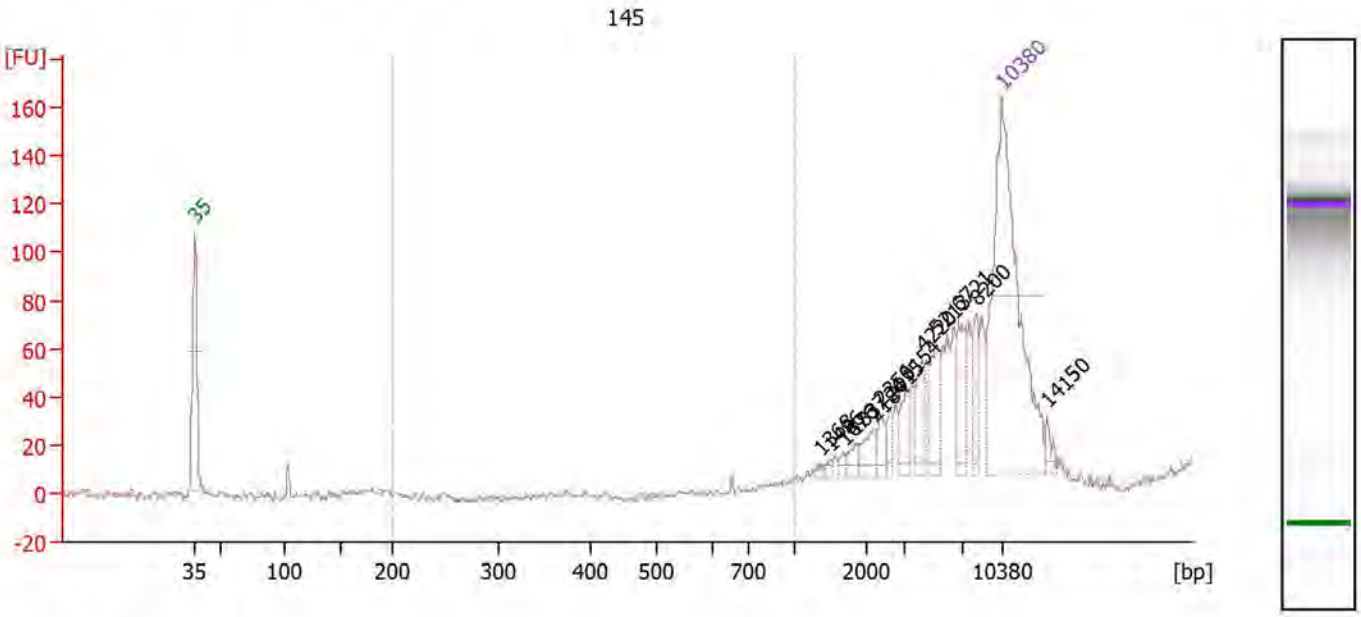

Overall Results for sample 11 : __145__

Number of peaks found:	13	Corr. Area 1:	0.0
Noise:	1.2		

Peak table for sample 11 : __145__

Peak		Size [bp]	Conc. [pg/µl]	Molarity [pmol/l]	Observations
1	◀	35	125.00	5,411.3	Lower Marker
2		1,368	0.89	1.0	
3		1,486	1.07	1.1	
4		1,676	1.58	1.4	
5		1,837	2.80	2.3	
6		2,184	5.67	3.9	
7		2,356	3.99	2.6	
8		2,615	3.30	1.9	
9		3,154	7.20	3.5	
10		4,220	8.15	2.9	
11		5,213	11.12	3.2	
12		6,721	10.82	2.4	
13		8,200	7.84	1.4	
14	▶	10,380	75.00	10.9	Upper Marker
15		14,150	0.00	0.0	

Region table for sample 11 : __145__

From [bp]	To [bp]	Corr. Area	% of Total	Average Size [bp]	Size distribution in CV [%]	Conc. [pg/µl]	Molarity [pmol/l]	Color
200	1,000	0.0	0	655	0.1	0.00	0.0	

Assay Class: High Sensitivity DNA Assay
Data Path: C:\...gh Sensitivity DNA Assay_DE04105532_2014-12-18_13-48-26.xad
Created: 12/18/2014 1:48:26 PM
Modified: 12/18/2014 2:29:50 PM

Gel Image

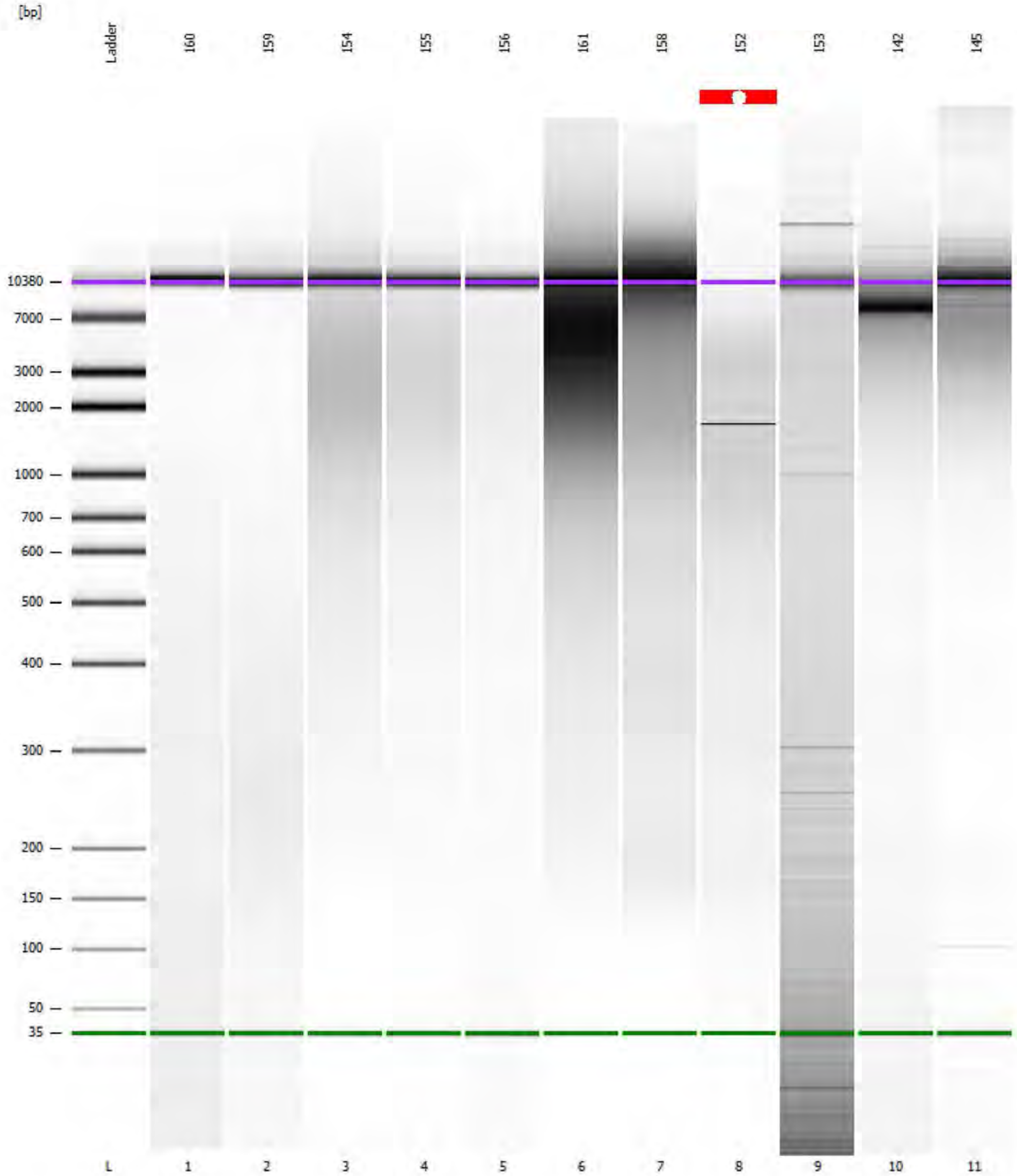

Assay Class: High Sensitivity DNA Assay
Data Path: C:\...gh Sensitivity DNA Assay_DE04105532_2014-12-18_13-48-26.xad

Created: 12/18/2014 1:48:26 PM
Modified: 12/18/2014 2:29:50 PM

Curves

Standard Curve

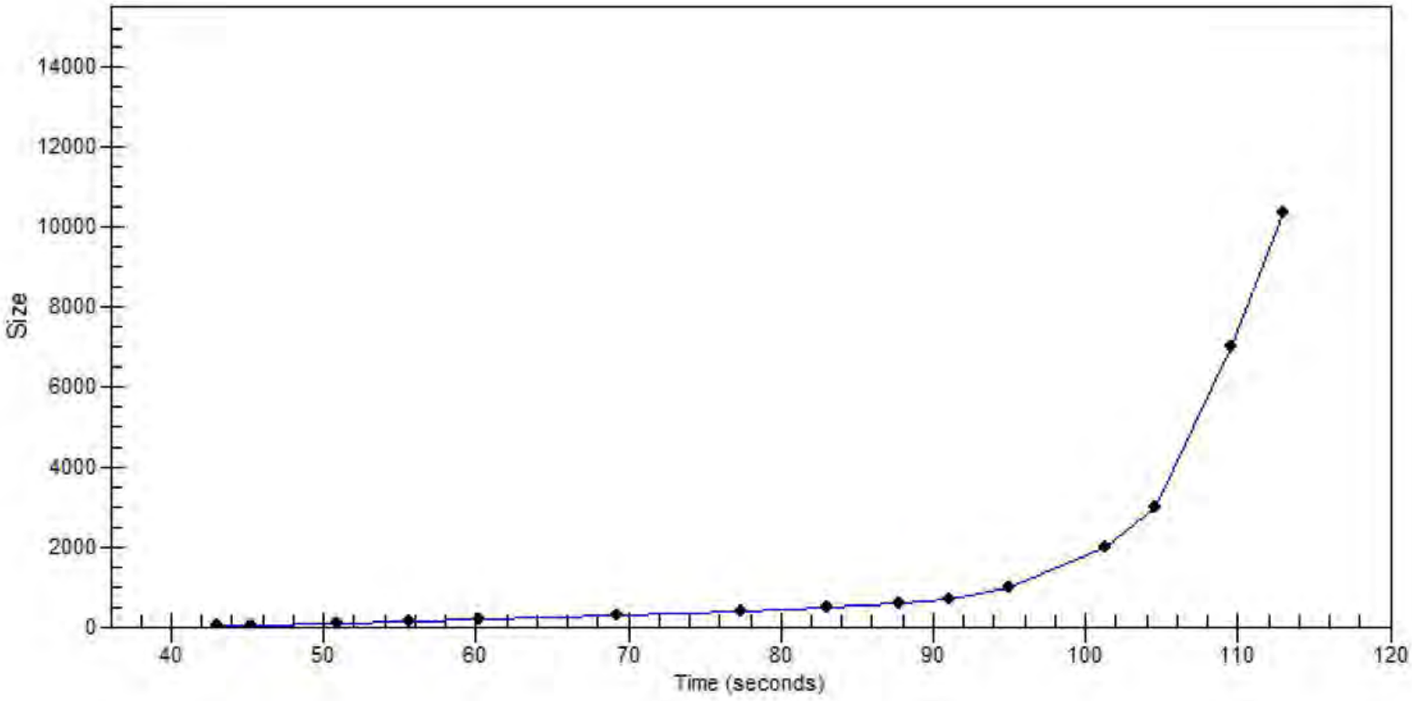

Assay Class: High Sensitivity DNA Assay
Data Path: C:\...gh Sensitivity DNA Assay_DE04105532_2014-12-18_13-48-26.xad

Created: 12/18/2014 1:48:26 PM
Modified: 12/18/2014 2:29:50 PM

Run Logbook

Description	Number	Source	Category	Sub Category	Time	Time Zone	User	Host
Run ended on port 2 (Number of wells acquired: 12)		Instrument	Run		12/18/2014 2:29:46 PM	(GMT +01:00) W. Europe Standard Time	Admin	Datasystem01
Instrument error occurred on port 2, Optical signal too high (1605h)	559	Instrument	Run	Sample 8	12/18/2014 2:20:41 PM	(GMT +01:00) W. Europe Standard Time	Admin	Datasystem01
Run started on port 2 (File: C:\Program Files\Agilent\2100 bioanalyzer\2100 expert\Data\2014-12-18\2100 expert_High Sensitivity DNA Assay_DE04105532_2014-12-18_13-48-26.xad)		Instrument	Run		12/18/2014 1:48:31 PM	(GMT +01:00) W. Europe Standard Time	Admin	Datasystem01
Product Number : G2938C		Instrument	Run		12/18/2014 1:48:31 PM	(GMT +01:00) W. Europe Standard Time	Admin	Datasystem01
Name :		Instrument	Run		12/18/2014 1:48:31 PM	(GMT +01:00) W. Europe Standard Time	Admin	Datasystem01
Vendor : Agilent Technologies		Instrument	Run		12/18/2014 1:48:31 PM	(GMT +01:00) W. Europe Standard Time	Admin	Datasystem01
Serial# : DE04105532		Instrument	Run		12/18/2014 1:48:31 PM	(GMT +01:00) W. Europe Standard Time	Admin	Datasystem01
Firmware : C.01.069		Instrument	Run		12/18/2014 1:48:31 PM	(GMT +01:00) W. Europe Standard Time	Admin	Datasystem01
Cartridge : Electrode		Instrument	Run		12/18/2014 1:48:31 PM	(GMT +01:00) W. Europe Standard Time	Admin	Datasystem01

Assay Class: High Sensitivity DNA Assay
Data Path: C:\...gh Sensitivity DNA Assay_DE04105532_2014-12-22_11-45-10.xad

Created: 12/22/2014 11:45:09 AM
Modified: 12/22/2014 12:26:42 PM

Electrophoresis File Run Summary

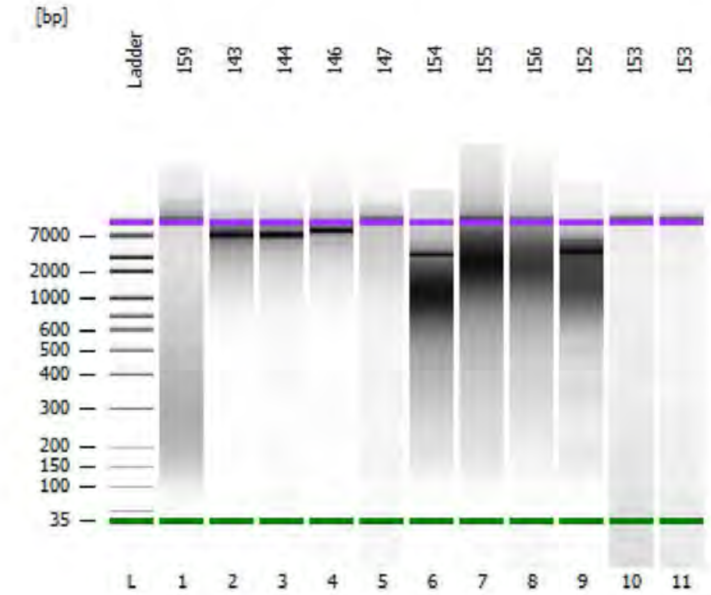

Instrument Information:

Instrument Name:	DE04105532	Firmware:	C.01.069
Serial#:	DE04105532	Type:	G2938C

Assay Information:

Assay Origin Path:	C:\Program Files\Agilent\2100 bioanalyzer\2100 expert\assays\dsDNA\High Sensitivity DNA.xsy
Assay Class:	High Sensitivity DNA Assay
Version:	1.03

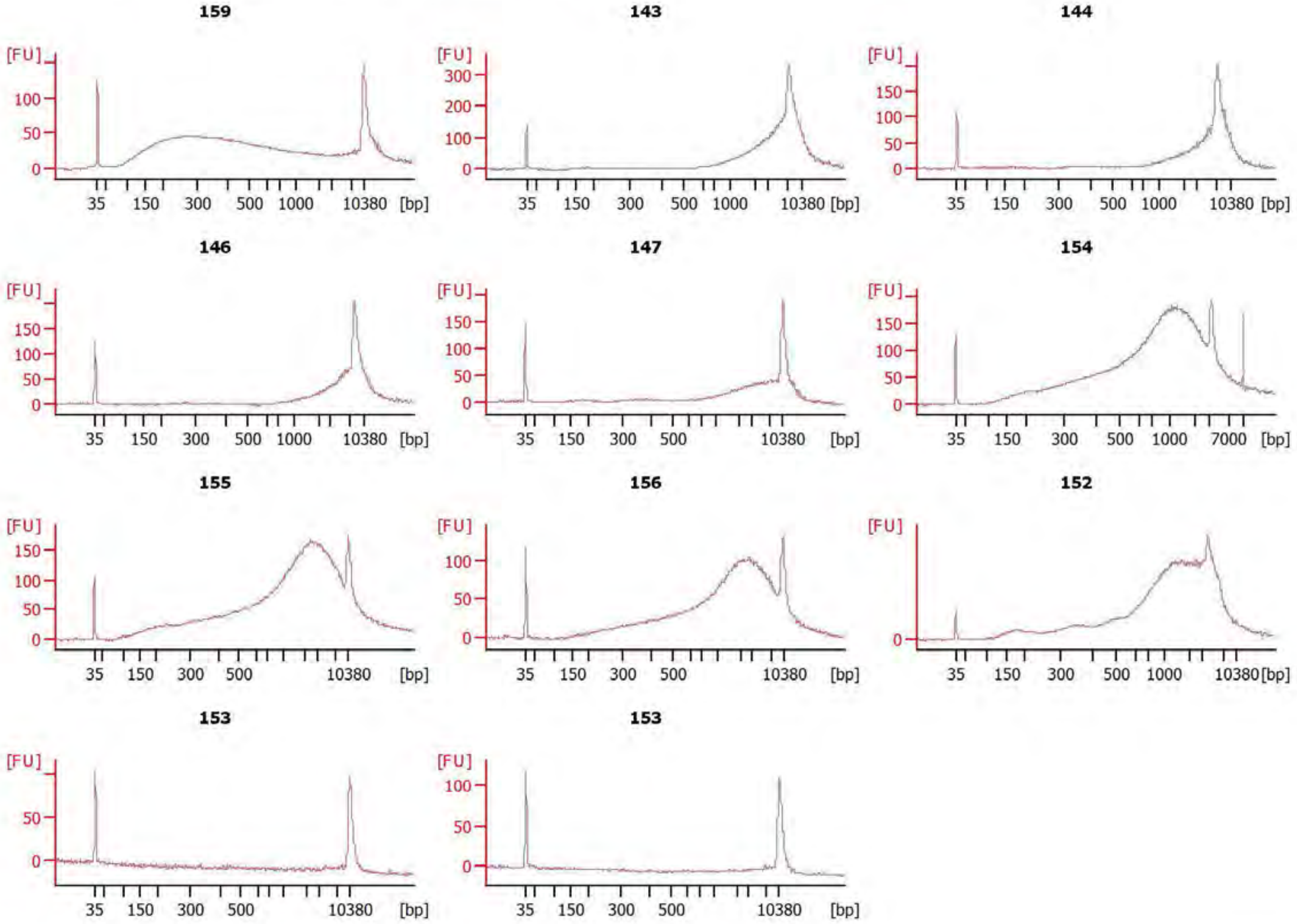

Assay Class: High Sensitivity DNA Assay
Data Path: C:\...gh Sensitivity DNA Assay_DE04105532_2014-12-22_11-45-10.xad

Created: 12/22/2014 11:45:09 AM
Modified: 12/22/2014 12:26:42 PM

Electrophoresis File Run Summary (Chip Summary)

Sample Name	Sample Comment	Rest. Digest	Status	Observation	Result Label	Result Color
159		☐	✔			
143		☐	✔			
144		☐	✔			
146		☐	✔			
147		☐	✔			
154		☐	✔			
155		☐	✔			
156		☐	✔			
152		☐	✔			
153		☐	✔			
153		☐	✔			
Ladder		☐	✔			

Chip Lot **Reagent Kit Lot**

Chip Comments :

Assay Class: High Sensitivity DNA Assay
Data Path: C:\...gh Sensitivity DNA Assay_DE04105532_2014-12-22_11-45-10.xad

Created: 12/22/2014 11:45:09 AM
Modified: 12/22/2014 12:26:42 PM

Electrophoresis Assay Details

General Analysis Settings

Number of Available Sample and Ladder Wells (Max.) : 12
Minimum Visible Range [s] : 32
Maximum Visible Range [s] : 138
Start Analysis Time Range [s] : 33
End Analysis Time Range [s] : 137.5
Ladder Concentration [pg/µl] : 1950
Uses Standard Area for Ladder Fragments
Lower Marker Concentration [pg/µl] : 125
Upper Marker Concentration [pg/µl] : 75
Used Upper Marker for Quantitation
Standard Curve Fit is Point to Point
Show Data Aligned to Lower and Upper Marker

Integrator Settings

Integration Start Time [s] : 33.05
Integration End Time [s] : 137
Slope Threshold : 0.8
Height Threshold [FU] : 5
Area Threshold : 0.1
Width Threshold [s] : 0.6
Baseline Plateau [s] : 0.5

Filter Settings

Filter Width [s] : 0.5
Polynomial Order : 4

Ladder

Ladder Peak	Size	Area
1	35	160
2	50	210
3	100	208
4	150	221
5	200	242
6	300	270
7	400	305
8	500	306
9	600	336
10	700	321
11	1000	366
12	2000	413
13	3000	411
14	7000	400
15	10380	214

Assay Class: High Sensitivity DNA Assay
Data Path: C:\...gh Sensitivity DNA Assay_DE04105532_2014-12-22_11-45-10.xad

Created: 12/22/2014 11:45:09 AM
Modified: 12/22/2014 12:26:42 PM

Electropherogram Summary

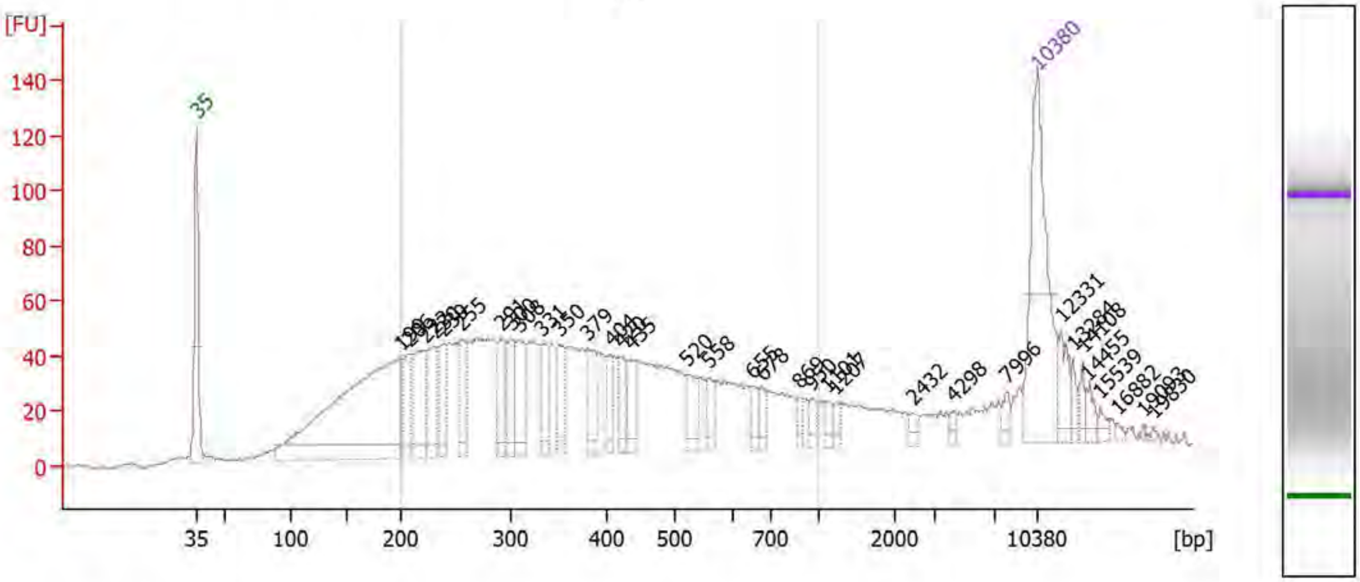

Overall Results for sample 1 : 159

Number of peaks found:	34	Corr. Area 1:	1,615.7
Noise:	0.6		

Peak table for sample 1 : 159

Peak	Size [bp]	Conc. [pg/µl]	Molarity [pmol/l]	Observations
1	35	125.00	5,411.3	Lower Marker
2	199	272.37	2,078.3	
3	206	27.06	198.7	
4	222	47.87	326.7	
5	230	33.00	217.5	
6	239	31.13	197.7	
7	255	28.89	171.8	
8	291	24.52	127.5	
9	300	23.91	120.7	
10	308	31.43	154.7	
11	331	24.25	111.0	
12	350	23.99	103.7	
13	379	26.49	105.9	
14	404	15.87	59.5	
15	420	17.44	62.8	
16	435	19.93	69.5	
17	520	17.36	50.6	
18	558	12.71	34.5	
19	655	8.79	20.3	
20	678	9.08	20.3	
21	869	5.62	9.8	
22	950	6.57	10.5	
23	1,101	4.95	6.8	
24	1,207	5.38	6.7	
25	2,432	3.90	2.4	
26	4,298	2.86	1.0	

Assay Class: High Sensitivity DNA Assay
Data Path: C:\...gh Sensitivity DNA Assay_DE04105532_2014-12-22_11-45-10.xad

Created: 12/22/2014 11:45:09 AM
Modified: 12/22/2014 12:26:42 PM

Electropherogram Summary Continued ...

... Peak table for sample 1 : 159

Peak	Size [bp]	Conc. [pg/µl]	Molarity [pmol/l]	Observations
27	7,996	4.90	0.9	
28	10,380	75.00	10.9	Upper Marker
29	12,331	0.00	0.0	
30	13,284	0.00	0.0	
31	14,108	0.00	0.0	
32	14,455	0.00	0.0	
33	15,539	0.00	0.0	
34	16,882	0.00	0.0	
35	19,093	0.00	0.0	
36	19,830	0.00	0.0	

Region table for sample 1 : 159

From [bp]	To [bp]	Corr. Area	% of Total	Average Size [bp]	Size distribution in CV [%]	Conc. [pg/µl]	Molarity [pmol/l]	Color
200	1,000	1,615.7	67	406	41.9	930.47	4,265.4	

Assay Class: High Sensitivity DNA Assay
Data Path: C:\...gh Sensitivity DNA Assay_DE04105532_2014-12-22_11-45-10.xad

Created: 12/22/2014 11:45:09 AM
Modified: 12/22/2014 12:26:42 PM

Electropherogram Summary Continued ...

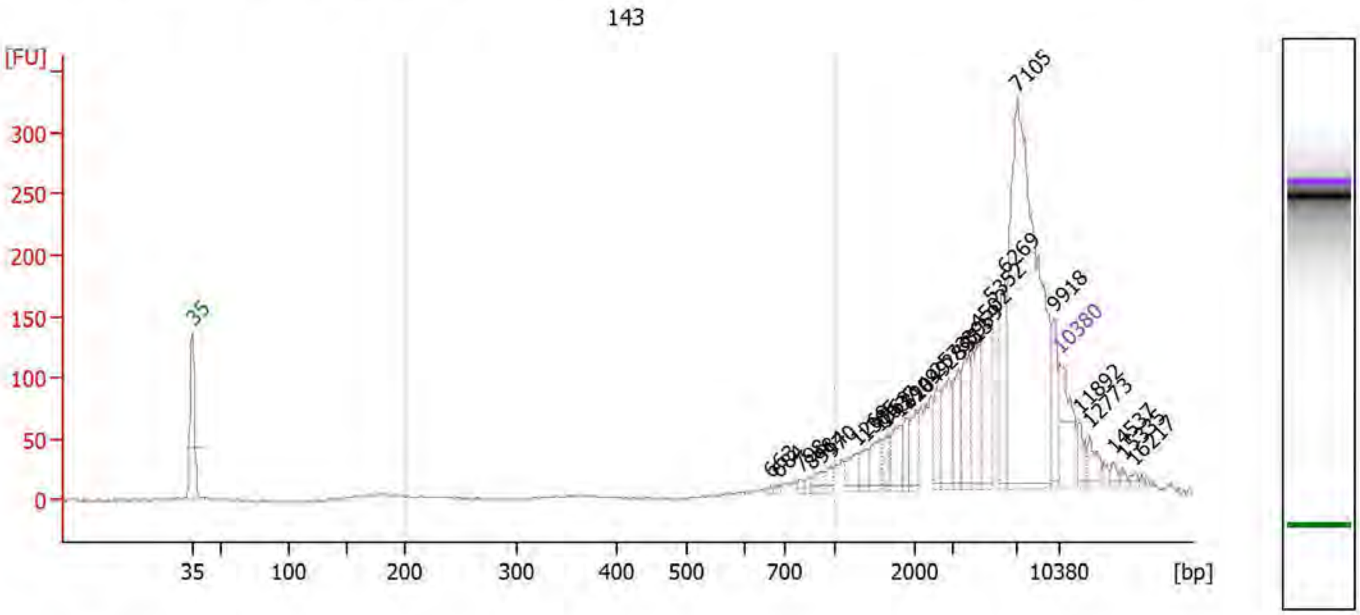

Overall Results for sample 2 : 143

Number of peaks found:	27	Corr. Area 1:	113.9
Noise:	0.6		

Peak table for sample 2 : 143

Peak		Size [bp]	Conc. [pg/µl]	Molarity [pmol/l]	Observations
1	◀	35	125.00	5,411.3	Lower Marker
2		663	3.46	7.9	
3		684	4.21	9.3	
4		798	6.65	12.6	
5		848	6.86	12.3	
6		914	15.90	26.4	
7		970	13.44	21.0	
8		1,269	28.44	33.9	
9		1,385	21.13	23.1	
10		1,562	30.78	29.9	
11		1,637	22.15	20.5	
12		1,814	38.19	31.9	
13		1,909	27.03	21.5	
14		2,049	34.58	25.6	
15		2,573	36.72	21.6	
16		2,895	58.09	30.4	
17		3,313	51.14	23.4	
18		3,959	57.66	22.1	
19		4,502	69.08	23.2	
20		5,352	101.51	28.7	
21		6,269	74.90	18.1	
22		7,105	575.99	122.8	
23		9,918	49.53	7.6	
24	▶	10,380	75.00	10.9	Upper Marker
25		11,892	0.00	0.0	
26		12,773	0.00	0.0	

Assay Class: High Sensitivity DNA Assay
Data Path: C:\...gh Sensitivity DNA Assay_DE04105532_2014-12-22_11-45-10.xad

Created: 12/22/2014 11:45:09 AM
Modified: 12/22/2014 12:26:42 PM

Electropherogram Summary Continued ...

... Peak table for sample 2 : 143

Peak	Size [bp]	Conc. [pg/µl]	Molarity [pmol/l]	Observations
27	14,537	0.00	0.0	
28	15,335	0.00	0.0	
29	16,217	0.00	0.0	

Region table for sample 2 : 143

From [bp]	To [bp]	Corr. Area	% of Total	Average Size [bp]	Size distribution in CV [%]	Conc. [pg/µl]	Molarity [pmol/l]	Color
200	1,000	113.9	5	779	21.9	104.38	238.2	

Assay Class: High Sensitivity DNA Assay
Data Path: C:\...gh Sensitivity DNA Assay_DE04105532_2014-12-22_11-45-10.xad

Created: 12/22/2014 11:45:09 AM
Modified: 12/22/2014 12:26:42 PM

Electropherogram Summary Continued ...

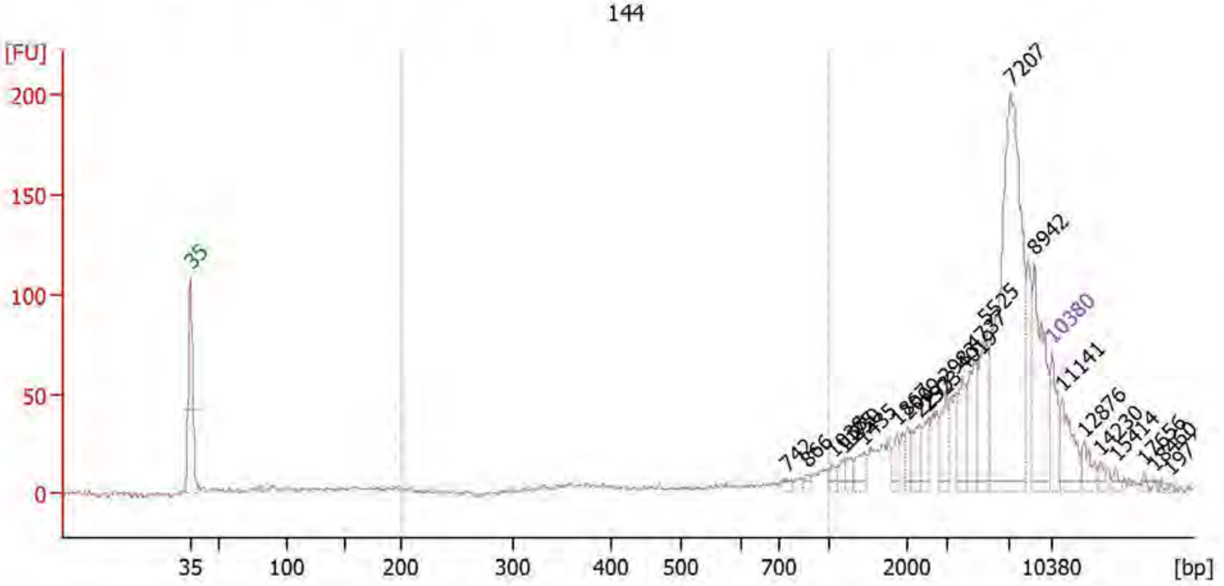

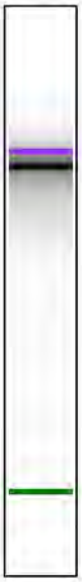

Overall Results for sample 3 : 144

Number of peaks found:	23	Corr. Area 1:	84.1
Noise:	1.0		

Peak table for sample 3 : 144

Peak		Size [bp]	Conc. [pg/µl]	Molarity [pmol/l]	Observations
1	◀	35	125.00	5,411.3	Lower Marker
2		742	16.80	34.3	
3		866	10.88	19.1	
4		1,038	17.43	25.5	
5		1,175	16.90	21.8	
6		1,230	25.18	31.0	
7		1,435	39.37	41.6	
8		1,867	30.88	25.1	
9		2,009	31.04	23.4	
10		2,293	47.00	31.1	
11		2,523	37.64	22.6	
12		2,983	58.55	29.7	
13		4,019	68.13	25.7	
14		4,737	96.04	30.7	
15		5,525	123.18	33.8	
16		7,207	693.44	145.8	
17		8,942	189.71	32.1	
18	▶	10,380	75.00	10.9	Upper Marker
19		11,141	0.00	0.0	
20		12,876	0.00	0.0	
21		14,230	0.00	0.0	
22		15,414	0.00	0.0	
23		17,656	0.00	0.0	
24		18,460	0.00	0.0	
25		19,771	0.00	0.0	

Assay Class: High Sensitivity DNA Assay
Data Path: C:\...gh Sensitivity DNA Assay_DE04105532_2014-12-22_11-45-10.xad

Created: 12/22/2014 11:45:09 AM
Modified: 12/22/2014 12:26:42 PM

Electropherogram Summary Continued ...

... Region table for sample 3 : 144

From [bp]	To [bp]	Corr. Area	% of Total	Average Size [bp]	Size distribution in CV [%]	Conc. [pg/µl]	Molarity [pmol/l]	Co lor
200	1,000	84.1	7	640	35.5	190.49	562.0	

Assay Class: High Sensitivity DNA Assay
Data Path: C:\...gh Sensitivity DNA Assay_DE04105532_2014-12-22_11-45-10.xad

Created: 12/22/2014 11:45:09 AM
Modified: 12/22/2014 12:26:42 PM

Electropherogram Summary Continued ...

146

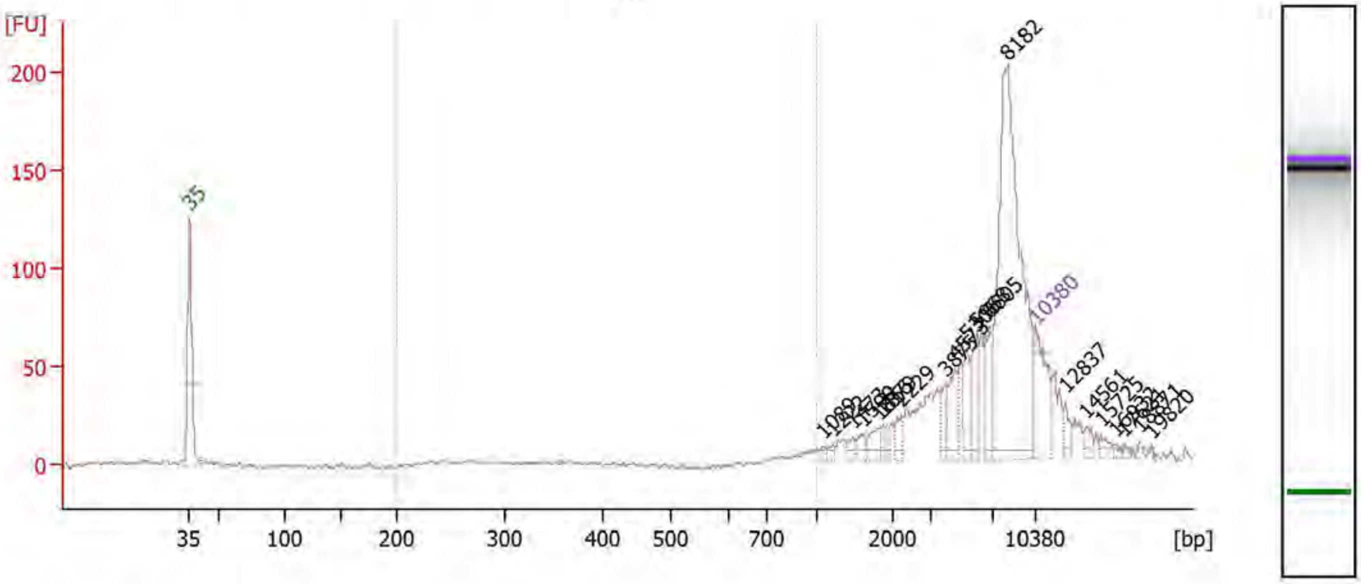

Overall Results for sample 4 : **146**

Number of peaks found:	20	Corr. Area 1:	1.0
Noise:	1.0		

Peak table for sample 4 : **146**

Peak		Size [bp]	Conc. [pg/µl]	Molarity [pmol/l]	Observations
1	◀	35	125.00	5,411.3	Lower Marker
2		1,089	5.85	8.1	
3		1,222	5.66	7.0	
4		1,473	9.63	9.9	
5		1,599	12.45	11.8	
6		1,816	17.43	14.5	
7		1,879	12.84	10.4	
8		2,229	16.90	11.5	
9		3,875	22.01	8.6	
10		4,573	36.89	12.2	
11		5,306	31.27	8.9	
12		5,968	42.22	10.7	
13		6,805	43.38	9.7	
14		8,182	412.45	76.4	
15	▶	10,380	75.00	10.9	Upper Marker
16		12,837	0.00	0.0	
17		14,561	0.00	0.0	
18		15,725	0.00	0.0	
19		16,932	0.00	0.0	
20		17,621	0.00	0.0	
21		18,871	0.00	0.0	
22		19,820	0.00	0.0	

Region table for sample 4 : **146**

From [bp]	To [bp]	Corr. Area	% of Total	Average Size [bp]	Size distribution in CV [%]	Conc. [pg/µl]	Molarity [pmol/l]	Color
200	1,000	1.0	0	963	2.6	1.21	1.9	■

Assay Class: High Sensitivity DNA Assay
Data Path: C:\...gh Sensitivity DNA Assay_DE04105532_2014-12-22_11-45-10.xad

Created: 12/22/2014 11:45:09 AM
Modified: 12/22/2014 12:26:42 PM

Electropherogram Summary Continued ...

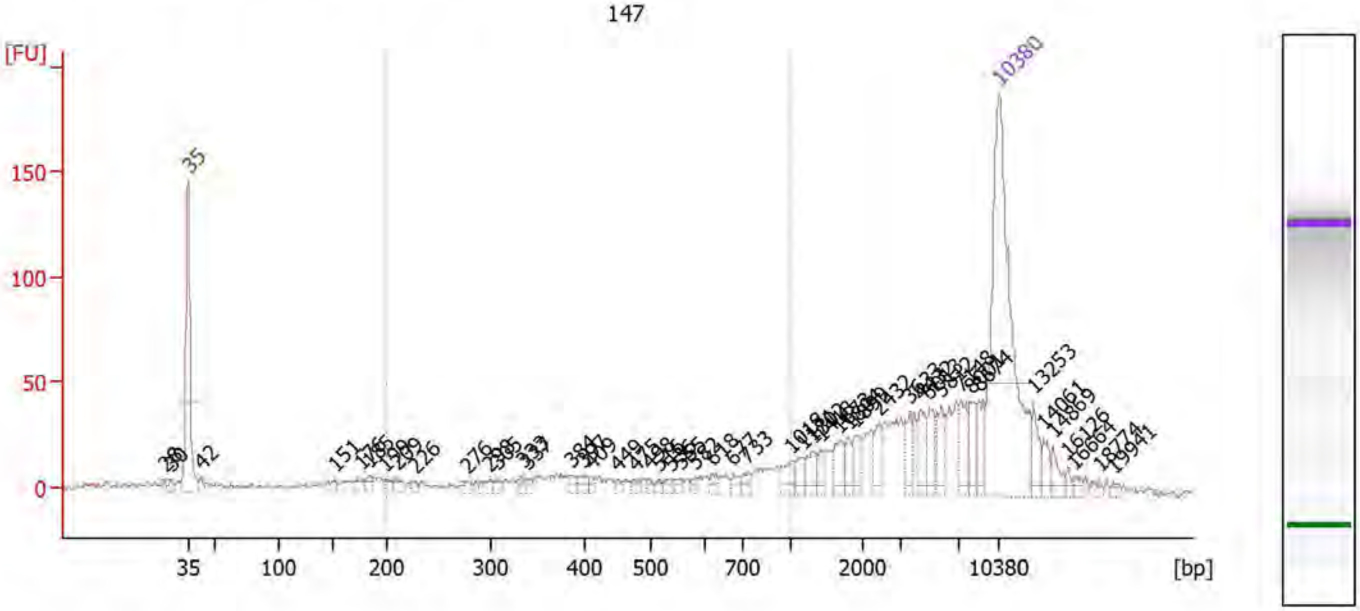

Overall Results for sample 5 : **147**

Number of peaks found:	49	Corr. Area 1:	241.3
Noise:	1.0		

Peak table for sample 5 : **147**

Peak		Size [bp]	Conc. [pg/µl]	Molarity [pmol/l]	Observations
1		28	0.00	0.0	
2		30	0.00	0.0	
3	◀	35	125.00	5,411.3	Lower Marker
4		42	3.49	124.9	
5		151	2.33	23.3	
6		176	4.46	38.4	
7		185	3.42	28.1	
8		199	2.82	21.4	
9		209	3.76	27.2	
10		226	1.95	13.1	
11		276	2.78	15.2	
12		298	3.35	17.0	
13		305	2.63	13.0	
14		333	2.86	13.0	
15		337	1.99	8.9	
16		384	2.34	9.2	
17		397	3.43	13.1	
18		409	1.94	7.2	
19		449	1.83	6.2	
20		475	2.04	6.5	
21		498	1.76	5.4	
22		516	1.54	4.5	
23		536	2.30	6.5	
24		555	2.10	5.7	
25		582	1.51	3.9	
26		618	2.48	6.1	

Assay Class: High Sensitivity DNA Assay
Data Path: C:\...gh Sensitivity DNA Assay_DE04105532_2014-12-22_11-45-10.xad
Created: 12/22/2014 11:45:09 AM
Modified: 12/22/2014 12:26:42 PM

Electropherogram Summary Continued ...

... Peak table for sample 5 : 147

Peak	Size [bp]	Conc. [pg/µl]	Molarity [pmol/l]	Observations
27	677	2.35	5.3	
28	733	3.19	6.6	
29	1,018	5.17	7.7	
30	1,171	3.98	5.2	
31	1,302	5.34	6.2	
32	1,418	3.83	4.1	
33	1,673	6.52	5.9	
34	1,804	3.88	3.3	
35	1,899	5.29	4.2	
36	2,432	5.70	3.6	
37	3,633	5.53	2.3	
38	4,432	7.41	2.5	
39	5,013	7.19	2.2	
40	5,812	7.54	2.0	
41	7,148	8.18	1.7	
42	8,001	6.36	1.2	
43	8,674	7.55	1.3	
44	10,380	75.00	10.9	Upper Marker
45	13,253	0.00	0.0	
46	14,061	0.00	0.0	
47	14,869	0.00	0.0	
48	16,126	0.00	0.0	
49	16,664	0.00	0.0	
50	18,774	0.00	0.0	
51	19,941	0.00	0.0	

Region table for sample 5 : 147

From [bp]	To [bp]	Corr. Area	% of Total	Average Size [bp]	Size distribution in CV [%]	Conc. [pg/µl]	Molarity [pmol/l]	Color
200	1,000	241.3	25	521	41.9	78.67	298.5	

Assay Class: High Sensitivity DNA Assay
Data Path: C:\...gh Sensitivity DNA Assay_DE04105532_2014-12-22_11-45-10.xad

Created: 12/22/2014 11:45:09 AM
Modified: 12/22/2014 12:26:42 PM

Electropherogram Summary Continued ...

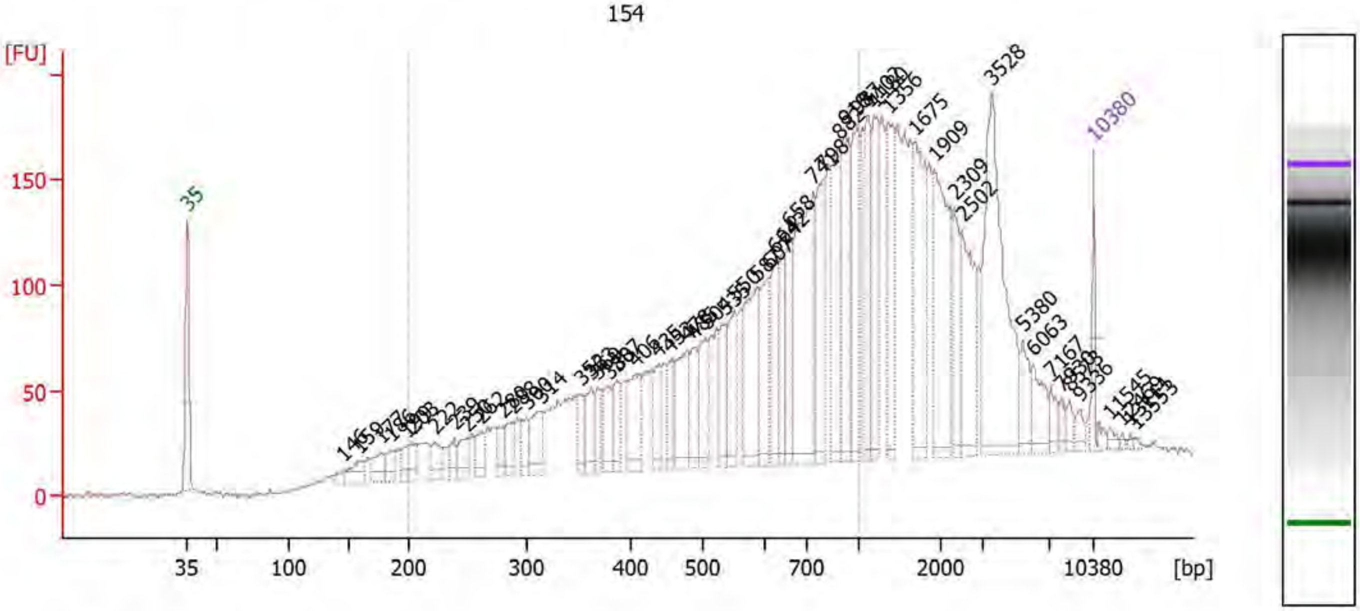

Overall Results for sample 6 : **154**

Number of peaks found:	55	Corr. Area 1:	2,710.0
Noise:	1.0		

Peak table for sample 6 : **154**

Peak	Size [bp]	Conc. [pg/µl]	Molarity [pmol/l]	Observations
1	35	125.00	5,411.3	Lower Marker
2	146	33.65	349.2	
3	159	93.43	890.9	
4	177	91.32	781.2	
5	186	71.63	584.9	
6	199	60.55	461.3	
7	203	64.78	483.7	
8	222	82.50	562.1	
9	239	72.89	462.7	
10	250	84.48	512.0	
11	262	85.74	496.2	
12	280	87.04	470.4	
13	288	86.17	453.3	
14	300	83.35	421.2	
15	314	157.33	759.0	
16	352	87.49	376.1	
17	363	128.31	536.1	
18	369	89.45	367.5	
19	380	129.30	516.2	
20	387	105.69	413.6	
21	406	180.69	674.7	
22	435	99.69	346.9	
23	453	103.57	346.5	
24	478	220.72	700.3	
25	485	137.26	429.0	
26	504	160.01	481.5	

Assay Class: High Sensitivity DNA Assay
Data Path: C:\...gh Sensitivity DNA Assay_DE04105532_2014-12-22_11-45-10.xad

Created: 12/22/2014 11:45:09 AM
Modified: 12/22/2014 12:26:42 PM

Electropherogram Summary Continued ...

... Peak table for sample 6 : 154

Peak	Size [bp]	Conc. [pg/µl]	Molarity [pmol/l]	Observations
27	535	124.29	352.2	
28	550	178.05	490.3	
29	587	334.85	865.0	
30	607	200.00	499.2	
31	624	180.84	439.1	
32	642	164.96	389.2	
33	658	190.27	438.3	
34	741	651.41	1,332.3	
35	798	272.79	518.0	
36	882	305.11	524.2	
37	918	293.93	485.2	
38	987	321.97	494.3	
39	1,102	221.52	304.6	
40	1,180	269.62	346.2	
41	1,356	271.95	303.9	
42	1,675	383.36	346.8	
43	1,909	402.00	319.0	
44	2,309	129.20	84.8	
45	2,502	280.97	170.1	
46	3,528	638.63	274.3	
47	5,380	80.22	22.6	
48	6,063	97.00	24.2	
49	7,167	40.32	8.5	
50	7,930	24.86	4.7	
51	8,573	25.54	4.5	
52	9,336	27.01	4.4	
53	10,380	75.00	10.9	Upper Marker
54	11,545	0.00	0.0	
55	12,469	0.00	0.0	
56	13,111	0.00	0.0	
57	13,553	0.00	0.0	

Region table for sample 6 : 154

From [bp]	To [bp]	Corr. Area	% of Total	Average Size [bp]	Size distribution in CV [%]	Conc. [pg/µl]	Molarity [pmol/l]	Co lor
200	1,000	2,710.0	57	583	36.4	7,408.63	24,520.2	

Assay Class: High Sensitivity DNA Assay
Data Path: C:\...gh Sensitivity DNA Assay_DE04105532_2014-12-22_11-45-10.xad

Created: 12/22/2014 11:45:09 AM
Modified: 12/22/2014 12:26:42 PM

Electropherogram Summary Continued ...

155

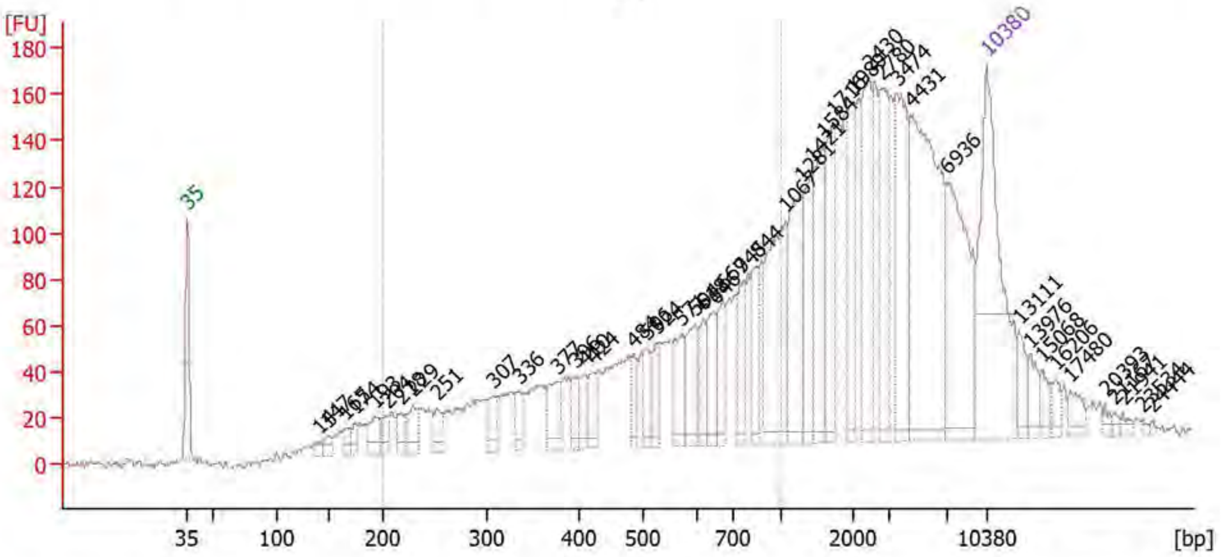

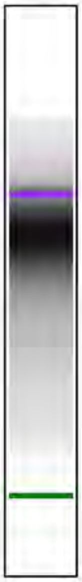

Overall Results for sample 7 : 155

Number of peaks found: 46

Noise: 1.5

Corr. Area 1: 1,687.2

Peak table for sample 7 : 155

Peak	Size [bp]	Conc. [pg/µl]	Molarity [pmol/l]	Observations
1	35	125.00	5,411.3	Lower Marker
2	141	4.29	46.2	
3	147	5.51	56.6	
4	165	6.71	61.5	
5	174	6.32	55.1	
6	193	13.08	102.9	
7	204	7.87	58.3	
8	218	10.37	72.0	
9	229	15.38	101.8	
10	251	10.04	60.5	
11	307	12.00	59.3	
12	336	10.83	48.8	
13	377	17.64	71.0	
14	396	8.86	33.9	
15	410	11.57	42.8	
16	424	11.13	39.8	
17	484	11.24	35.2	
18	506	10.85	32.5	
19	524	12.52	36.2	
20	571	19.56	51.9	
21	594	21.21	54.1	
22	618	13.85	34.0	
23	646	17.73	41.6	
24	669	18.90	42.8	
25	745	16.41	33.4	
26	844	18.27	32.8	

Assay Class: High Sensitivity DNA Assay
Data Path: C:\...gh Sensitivity DNA Assay_DE04105532_2014-12-22_11-45-10.xad
Created: 12/22/2014 11:45:09 AM
Modified: 12/22/2014 12:26:42 PM

Electropherogram Summary Continued ...

... Peak table for sample 7 : 155

Peak	Size [bp]	Conc. [pg/µl]	Molarity [pmol/l]	Observations
27	1,067	57.51	81.7	
28	1,281	44.89	53.1	
29	1,421	26.11	27.8	
30	1,584	35.44	33.9	
31	1,716	32.30	28.5	
32	1,989	24.94	19.0	
33	2,430	38.81	24.2	
34	2,780	39.66	21.6	
35	3,474	48.35	21.1	
36	4,431	101.86	34.8	
37	6,936	61.22	13.4	
38	10,380	75.00	10.9	Upper Marker
39	13,111	0.00	0.0	
40	13,976	0.00	0.0	
41	15,068	0.00	0.0	
42	16,206	0.00	0.0	
43	17,480	0.00	0.0	
44	20,393	0.00	0.0	
45	21,167	0.00	0.0	
46	21,941	0.00	0.0	
47	23,534	0.00	0.0	
48	24,444	0.00	0.0	

Region table for sample 7 : 155

From [bp]	To [bp]	Corr. Area	% of Total	Average Size [bp]	Size distribution in CV [%]	Conc. [pg/µl]	Molarity [pmol/l]	Color
200	1,000	1,687.2	40	550	39.2	592.00	2,113.3	

Assay Class: High Sensitivity DNA Assay
Data Path: C:\...gh Sensitivity DNA Assay_DE04105532_2014-12-22_11-45-10.xad

Created: 12/22/2014 11:45:09 AM
Modified: 12/22/2014 12:26:42 PM

Electropherogram Summary Continued ...

156

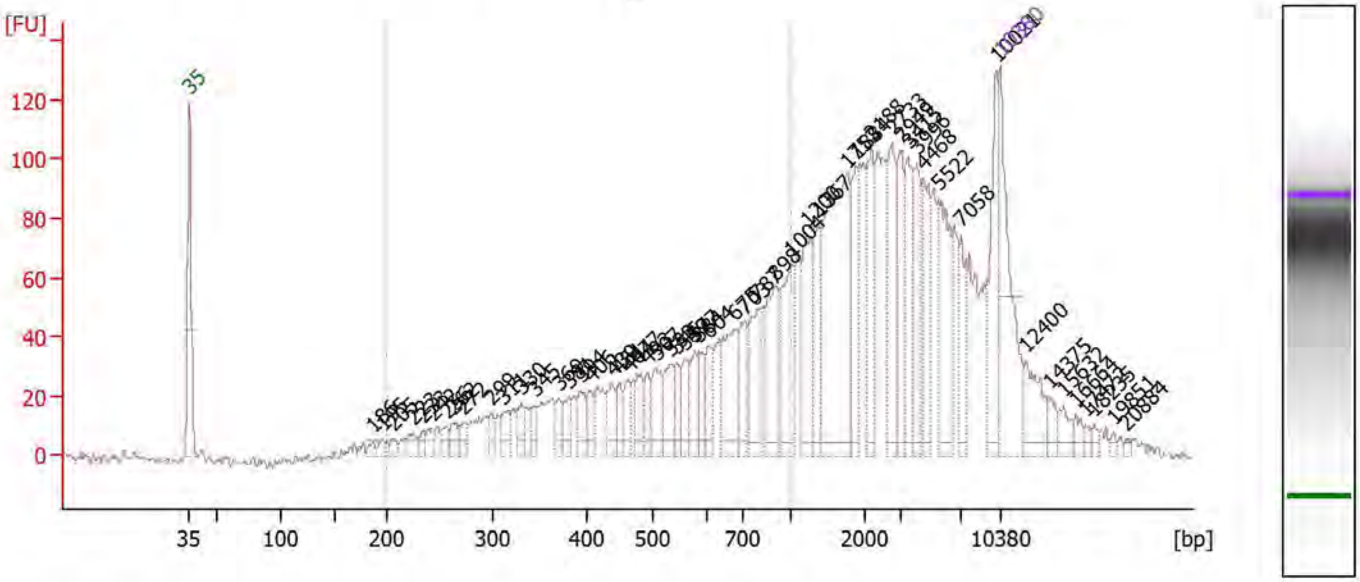

Overall Results for sample 8 : __156__

Number of peaks found:	55	Corr. Area 1:	1,066.2
Noise:	1.0		

Peak table for sample 8 : __156__

Peak		Size [bp]	Conc. [pg/µl]	Molarity [pmol/l]	Observations
1	◀	35	125.00	5,411.3	Lower Marker
2		186	5.38	43.8	
3		195	5.65	43.9	
4		205	6.37	47.1	
5		223	13.24	89.8	
6		233	7.13	46.3	
7		242	9.74	61.0	
8		256	10.86	64.3	
9		262	11.71	67.8	
10		272	11.44	63.8	
11		299	10.69	54.2	
12		311	16.38	79.8	
13		330	13.23	60.7	
14		345	11.73	51.5	
15		369	13.27	54.4	
16		381	14.22	56.5	
17		394	17.40	66.9	
18		406	14.21	53.0	
19		439	19.02	65.6	
20		451	15.13	50.9	
21		464	14.54	47.5	
22		477	20.36	64.6	
23		493	15.67	48.2	
24		507	27.18	81.2	
25		533	25.08	71.3	
26		545	16.53	46.0	

Assay Class: High Sensitivity DNA Assay
Data Path: C:\...gh Sensitivity DNA Assay_DE04105532_2014-12-22_11-45-10.xad

Created: 12/22/2014 11:45:09 AM
Modified: 12/22/2014 12:26:42 PM

Electropherogram Summary Continued ...

... Peak table for sample 8 : 156

Peak		Size [bp]	Conc. [pg/µl]	Molarity [pmol/l]	Observations
27		559	18.15	49.2	
28		577	22.48	59.0	
29		584	17.65	45.8	
30		604	20.67	51.9	
31		675	51.83	116.3	
32		703	27.26	58.8	
33		787	39.93	76.9	
34		898	48.66	82.1	
35		1,004	54.61	82.4	
36		1,200	46.77	59.0	
37		1,367	34.41	38.1	
38		1,753	136.82	118.2	
39		1,884	34.11	27.4	
40		2,188	42.72	29.6	
41		2,733	45.35	25.1	
42		2,949	39.16	20.1	
43		3,415	43.25	19.2	
44		3,996	37.20	14.1	
45		4,468	39.82	13.5	
46		5,522	54.15	14.9	
47		7,058	24.75	5.3	
48		10,021	47.34	7.2	
49	▶	10,380	75.00	10.9	Upper Marker
50		12,400	0.00	0.0	
51		14,375	0.00	0.0	
52		15,632	0.00	0.0	
53		16,664	0.00	0.0	
54		17,472	0.00	0.0	
55		18,235	0.00	0.0	
56		19,851	0.00	0.0	
57		20,884	0.00	0.0	

Region table for sample 8 : 156

From [bp]	To [bp]	Corr. Area	% of Total	Average Size [bp]	Size distribution in CV [%]	Conc. [pg/µl]	Molarity [pmol/l]	Co lor
200	1,000	1,066.2	39	565	37.0	820.17	2,769.1	■

Assay Class: High Sensitivity DNA Assay
Data Path: C:\...gh Sensitivity DNA Assay_DE04105532_2014-12-22_11-45-10.xad

Created: 12/22/2014 11:45:09 AM
Modified: 12/22/2014 12:26:42 PM

Electropherogram Summary Continued ...

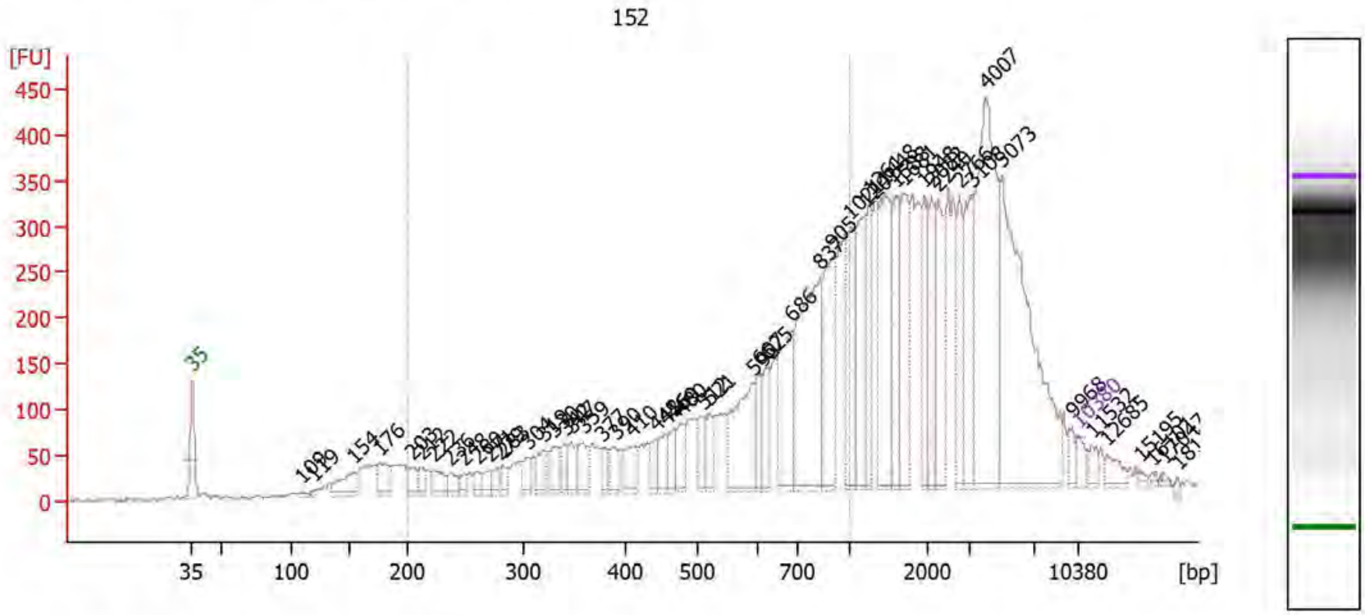

Overall Results for sample 9 : 152

Number of peaks found:	54	Corr. Area 1:	3,631.1
Noise:	2.0		

Peak table for sample 9 : 152

Peak	Size [bp]	Conc. [pg/µl]	Molarity [pmol/l]	Observations
1	35	125.00	5,411.3	Lower Marker
2	109	27.14	376.6	
3	119	25.35	322.2	
4	154	280.31	2,761.5	
5	176	260.49	2,247.1	
6	203	142.98	1,067.1	
7	212	92.25	658.4	
8	222	177.98	1,217.1	
9	236	120.98	776.9	
10	248	63.20	385.4	
11	260	83.10	484.2	
12	271	99.36	556.1	
13	279	85.24	463.6	
14	283	84.20	451.1	
15	304	131.93	658.4	
16	319	155.38	737.2	
17	330	180.87	830.9	
18	342	129.31	573.1	
19	347	147.07	642.0	
20	359	177.47	748.7	
21	377	100.56	403.6	
22	390	142.00	551.6	
23	410	199.57	738.1	
24	442	139.45	477.7	
25	456	168.67	559.8	
26	469	143.91	464.8	

Assay Class: High Sensitivity DNA Assay
Data Path: C:\...gh Sensitivity DNA Assay_DE04105532_2014-12-22_11-45-10.xad

Created: 12/22/2014 11:45:09 AM
Modified: 12/22/2014 12:26:42 PM

Electropherogram Summary Continued ...

... Peak table for sample 9 : 152

Peak	Size [bp]	Conc. [pg/µl]	Molarity [pmol/l]	Observations
27	480	187.19	590.5	
28	512	188.26	557.2	
29	521	145.36	422.9	
30	593	617.08	1,575.6	
31	607	209.47	522.5	
32	625	238.17	577.6	
33	686	581.58	1,284.2	
34	837	1,212.14	2,193.6	
35	905	680.15	1,138.8	
36	1,021	514.13	763.3	
37	1,201	514.07	648.7	
38	1,261	382.32	459.4	
39	1,448	761.41	796.9	
40	1,588	428.78	409.2	
41	1,681	480.81	433.3	
42	1,948	343.42	267.1	
43	2,082	364.55	265.3	
44	2,240	431.68	292.0	
45	2,766	326.24	178.7	
46	3,108	506.89	247.1	
47	4,007	1,409.51	533.0	
48	5,073	1,772.98	529.6	
49	9,968	65.94	10.0	
50	10,380	75.00	10.9	Upper Marker
51	11,532	0.00	0.0	
52	12,685	0.00	0.0	
53	15,195	0.00	0.0	
54	16,101	0.00	0.0	
55	17,047	0.00	0.0	
56	18,117	0.00	0.0	

Region table for sample 9 : 152

From [bp]	To [bp]	Corr. Area	% of Total	Average Size [bp]	Size distribution in CV [%]	Conc. [pg/µl]	Molarity [pmol/l]	Color
200	1,000	3,631.1	40	609	36.0	9,027.86	28,936.2	

Assay Class: High Sensitivity DNA Assay
Data Path: C:\...gh Sensitivity DNA Assay_DE04105532_2014-12-22_11-45-10.xad

Created: 12/22/2014 11:45:09 AM
Modified: 12/22/2014 12:26:42 PM

Electropherogram Summary Continued ...

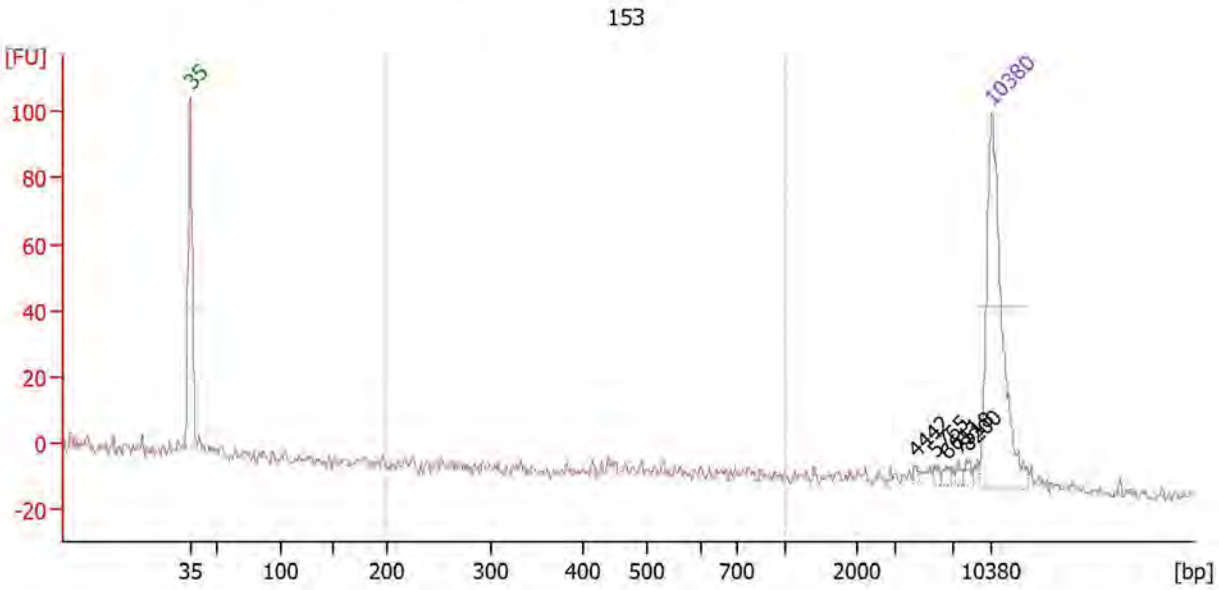

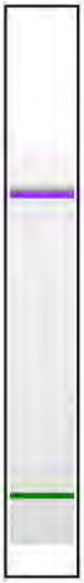

Overall Results for sample 10 : 153

Number of peaks found:	5	Corr. Area 1:	16.0
Noise:	1.9		

Peak table for sample 10 : 153

Peak		Size [bp]	Conc. [pg/µl]	Molarity [pmol/l]	Observations
1	◀	35	125.00	5,411.3	Lower Marker
2		4,442	1.29	0.4	
3		5,765	1.50	0.4	
4		6,611	2.08	0.5	
5		7,518	1.72	0.3	
6		8,200	2.37	0.4	
7	▶	10,380	75.00	10.9	Upper Marker

Region table for sample 10 : 153

From [bp]	To [bp]	Corr. Area	% of Total	Average Size [bp]	Size distribution in CV [%]	Conc. [pg/µl]	Molarity [pmol/l]	Color
200	1,000	16.0	18	568	29.4	10.40	32.0	■

Assay Class: High Sensitivity DNA Assay
Data Path: C:\...gh Sensitivity DNA Assay_DE04105532_2014-12-22_11-45-10.xad

Created: 12/22/2014 11:45:09 AM
Modified: 12/22/2014 12:26:42 PM

Electropherogram Summary Continued ...

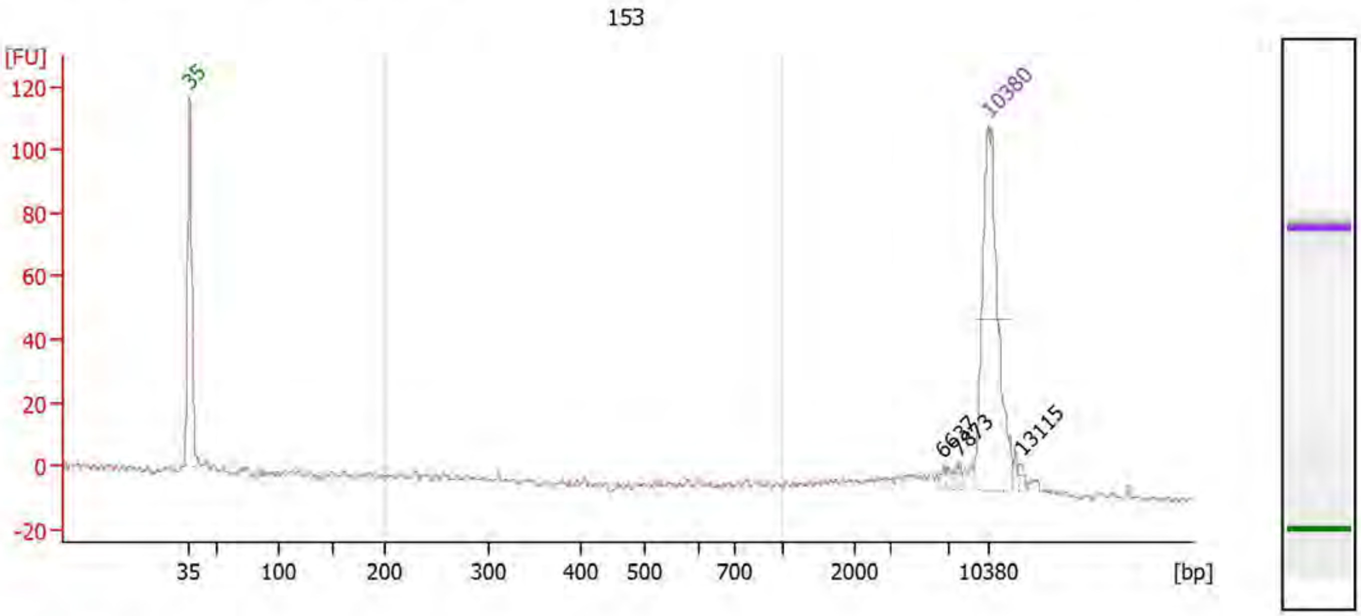

Overall Results for sample 11 : 153

Number of peaks found:	3	Corr. Area 1:	16.9
Noise:	0.9		

Peak table for sample 11 : 153

Peak		Size [bp]	Conc. [pg/µl]	Molarity [pmol/l]	Observations
1	◀	35	125.00	5,411.3	Lower Marker
2		6,637	1.53	0.3	
3		7,873	2.55	0.5	
4	▶	10,380	75.00	10.9	Upper Marker
5		13,115	0.00	0.0	

Region table for sample 11 : 153

From [bp]	To [bp]	Corr. Area	% of Total	Average Size [bp]	Size distribution in CV [%]	Conc. [pg/µl]	Molarity [pmol/l]	Co lor
200	1,000	16.9	13	575	43.5	10.99	42.5	■

Assay Class: High Sensitivity DNA Assay
Data Path: C:\...gh Sensitivity DNA Assay_DE04105532_2014-12-22_11-45-10.xad

Created: 12/22/2014 11:45:09 AM
Modified: 12/22/2014 12:26:42 PM

Gel Image

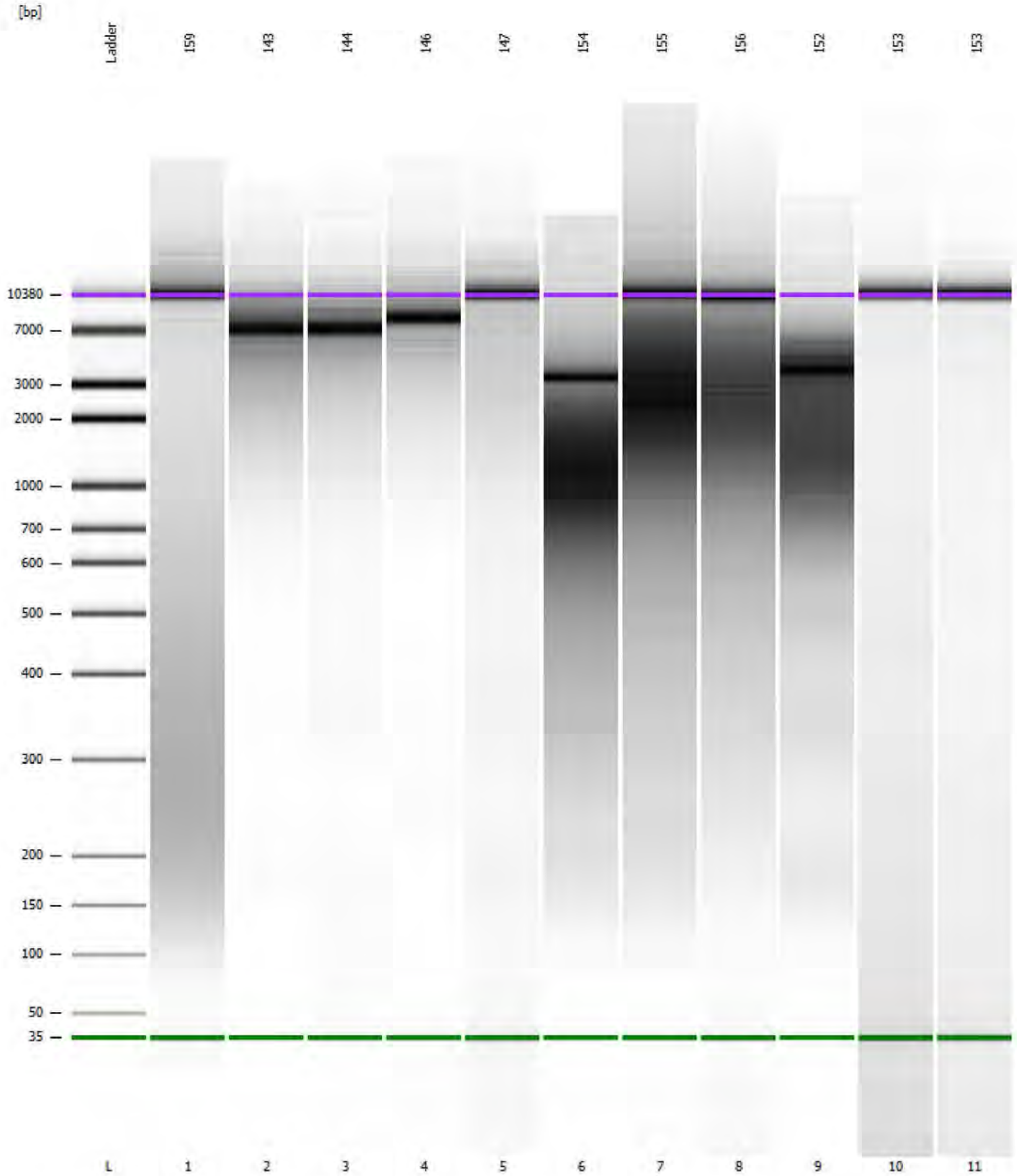

Assay Class: High Sensitivity DNA Assay
Data Path: C:\...gh Sensitivity DNA Assay_DE04105532_2014-12-22_11-45-10.xad

Created: 12/22/2014 11:45:09 AM
Modified: 12/22/2014 12:26:42 PM

Curves

Standard Curve

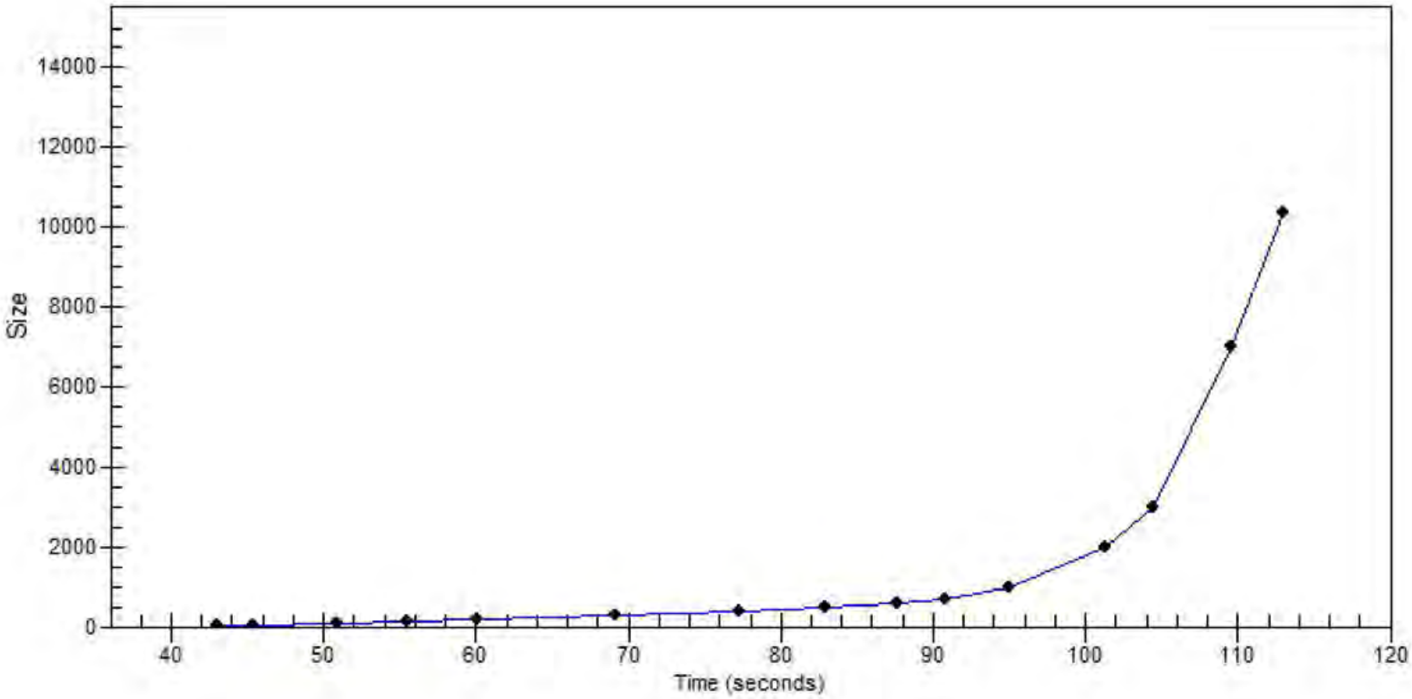

Assay Class: High Sensitivity DNA Assay
Data Path: C:\...gh Sensitivity DNA Assay_DE04105532_2014-12-22_11-45-10.xad

Created: 12/22/2014 11:45:09 AM
Modified: 12/22/2014 12:26:42 PM

Run Logbook

Description	Number	Source	Category	Sub Category	Time	Time Zone	User	Host
Run ended on port 2 (Number of wells acquired: 12)		Instrument	Run		12/22/2014 12:26:30 PM	(GMT +01:00) W. Europe Standard Time	Admin	Datasystem01
Run started on port 2 (File: C:\Program Files\Agilent\2100 bioanalyzer\2100 expert\Data\2014-12-22\2100 expert_High Sensitivity DNA Assay_DE04105532_2014-12-22_11-45-10.xad)		Instrument	Run		12/22/2014 11:45:15 AM	(GMT +01:00) W. Europe Standard Time	Admin	Datasystem01
Product Number : G2938C		Instrument	Run		12/22/2014 11:45:15 AM	(GMT +01:00) W. Europe Standard Time	Admin	Datasystem01
Name :		Instrument	Run		12/22/2014 11:45:15 AM	(GMT +01:00) W. Europe Standard Time	Admin	Datasystem01
		Instrument	Run		12/22/2014 11:45:15 AM	(GMT +01:00) W. Europe Standard Time	Admin	Datasystem01
Serial# : DE04105532		Instrument	Run		12/22/2014 11:45:15 AM	(GMT +01:00) W. Europe Standard Time	Admin	Datasystem01
Firmware : C.01.069		Instrument	Run		12/22/2014 11:45:15 AM	(GMT +01:00) W. Europe Standard Time	Admin	Datasystem01
Cartridge : Electrode		Instrument	Run		12/22/2014 11:45:15 AM	(GMT +01:00) W. Europe Standard Time	Admin	Datasystem01
